# Anionic Olefin Metathesis Catalysts Enable Modification of Unprotected Biomolecules in Water

**DOI:** 10.1021/acscatal.4c02811

**Published:** 2024-07-11

**Authors:** Christian O. Blanco, Richard Ramos Castellanos, Deryn E. Fogg

**Affiliations:** † Center for Catalysis Research & Innovation, and Department of Chemistry and Biomolecular Sciences, University of Ottawa, Ottawa, Ontario, K1N 6N5, Canada; ‡ Department of Chemistry, University of Bergen, Allégaten 41, N-5007 Bergen, Norway

**Keywords:** olefin metathesis, ruthenium, aqueous metathesis, chemical biology, sulfonates, anionic ligand, isomerization

## Abstract

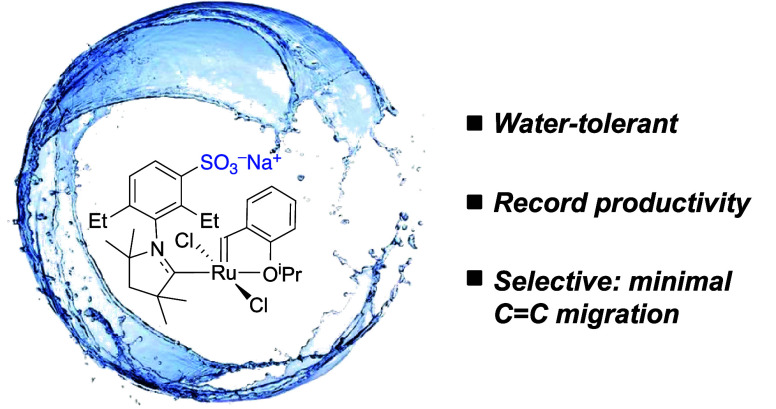

Stability problems have limited the uptake of cationic olefin metathesis catalysts in chemical biology. Described herein are anionic catalysts that improve water-solubility, robustness, and compatibility with biomolecules such as DNA. A sulfonate tag is installed on the cyclic (alkyl)(amino) carbene (CAAC) ligand platform, chosen for resistance to degradation by nucleophiles, base, water, and β-elimination. Hoveyda–Grubbs catalysts bearing the sulfonated CAAC ligands deliver record productivity in metathesis of unprotected carbohydrates and nucleosides at neutral pH. Decomposed catalyst has negligible impact on metathesis selectivity, whereas N-heterocyclic carbene (NHC) catalysts degrade rapidly in water and cause extensive C=C migration.

Olefin metathesis catalysts capable of modifying unprotected biomolecules hold great potential as a tool that bridges chemistry and biology.^1−4^ Modification of peptides or proteins (Figure 1) has seen much study.^4c,5^ Other, emerging applications include drug discovery via DNA-encoded libraries (DEL),^6,7^ and in vivo metathesis in blood^8^ or living cells.^9−11^ Most of these applications require metathesis in water. Surprising, therefore, is the widespread preference for neutral catalysts such as **HII** (the second-generation Hoveyda–Grubbs catalyst; Chart 1a),^4−7^ despite the availability of water-soluble analogues (Chart 1b).^12−15^ In several comparative studies, water-soluble catalysts (e.g., Aquamet, **AM**; **Ru-1**) proved less effective than **HII** in mixed organic-aqueous media,^5,9,6,16^ notwithstanding superior phase homogeneity.^6^

**Figure 1:**
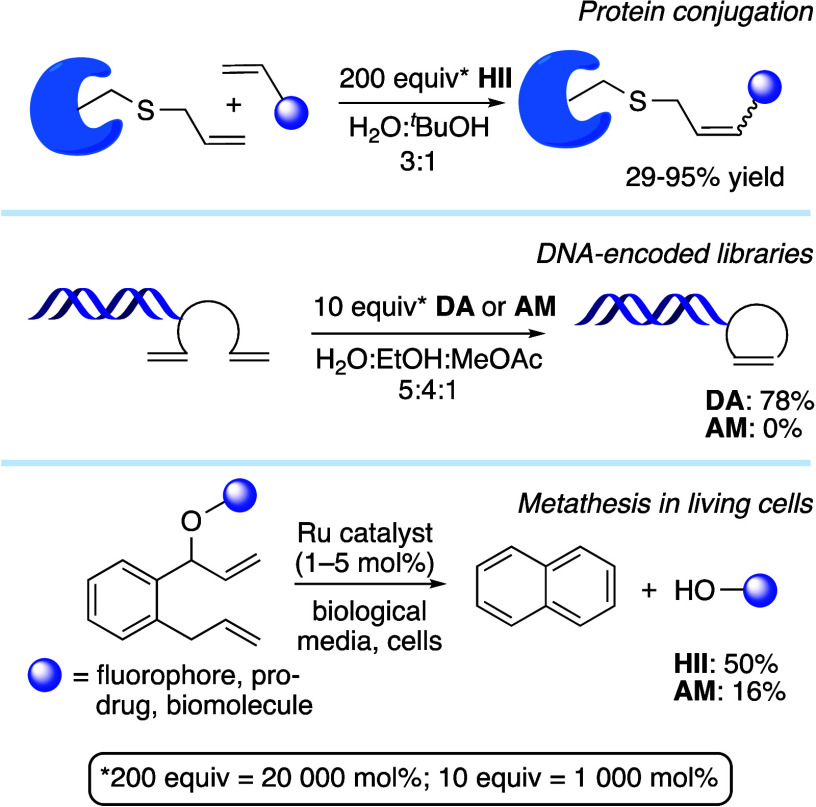
Selected applications of olefin metathesis in chemical biology. For catalysts, see Chart 1.

**Chart 1:**
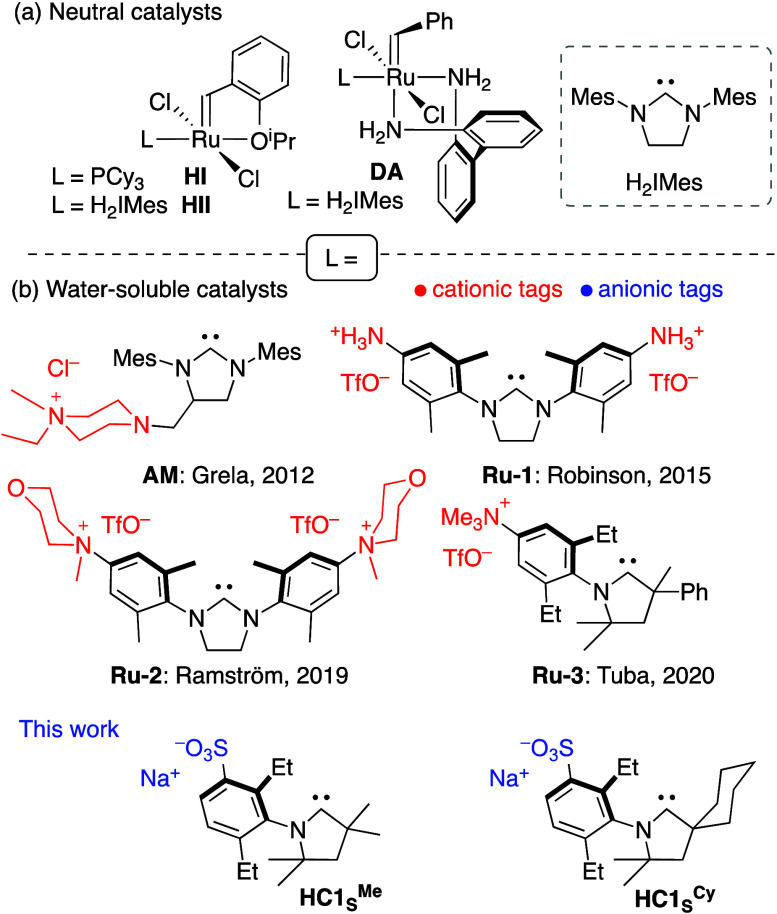
(a) Neutral Metathesis Catalysts Cited in Figure 1; (b) Charged, Water-Soluble Catalysts That Enable Metathesis in Water

We questioned whether the ubiquitous reliance on cationic tags for water-solubility^17,18^ (see Chart 1b) might have unforeseen negative impacts. The failure of cationic **AM** in DEL applications, accompanied by DNA degradation, has been attributed to electrostatic attraction between the ammonium group and the negatively charged phosphate backbone of DNA.^6^ As a more general hazard, cationic ligands may increase the acidity of ligated water in the [Ru]–OH_2_ (aqua complexes) formed in water,^19−22^ accelerating decomposition into metathesis-inactive^23^ Ru-hydroxides. Anionic tags offer a compelling alternative, reinforced by their potential to enhance water-solubility via participation in extended H-bonding networks.^24,25^ Maximizing catalyst concentrations in water is crucial in chemical biology, which has been characterized as a race between metathesis and decomposition.^4c^

In undertaking catalyst redesign, we therefore prioritized anionic charge. We chose weakly basic sulfonate tags,^26^ which confer high solubility, remain anionic over a wide pH range,^27^ and show low affinity with ruthenium unless driven by chelation^26a,26c^ or silver salts.^26b^ Two recent studies of Suzuki–Miyaura coupling in water describe the compatibility of sulfonate-tagged phosphines with DNA.^28,29^ A further criterion was installation on a cyclic alkyl amino carbene (CAAC) ligand. CAACs are privileged ligands in metathesis relative to their phosphine and N-heterocyclic carbene (NHC) predecessors. Within Hoveyda-class catalysts, CAACs improve resistance to degradation by (inter alia) water, nucleophiles, Brønsted base, and β-elimination.^30^ Ammonium-functionalized **Ru-3** (Chart 1b) is the sole charged CAAC catalyst reported to date.^31^ Here we describe anionic CAAC catalysts that deliver record performance in the metathesis of terminal olefins in water, including unprotected nucleoside and carbohydrate derivatives.

Direct *m*-sulfonation of the known CAAC•HBF_4_ salts with fuming sulfuric acid, via protocols established in NHC chemistry,^32^ offers the most direct entry to the anionic carbenes (Scheme 1a, top). Monosulfonated products were obtained in ca. 80% yield for proligands bearing a single aromatic ring. Salts bearing a second aromatic group yielded a mixture of polysulfonated products, and were not pursued.

**Scheme 1:**
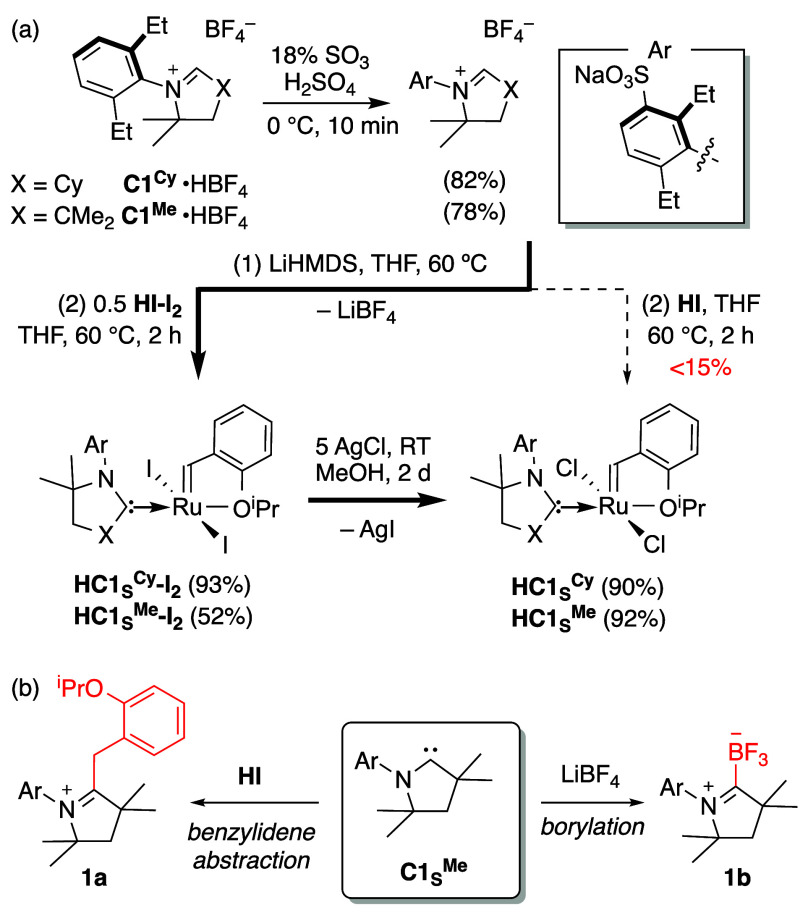
(a) Synthesis of Sulfonate-Tagged CAAC Ligands and Catalysts; (b) Side-Reactions Resulting in Consumption of **C1**_S_^Me^

The major electronic and solubility properties that differentiate the anionic carbenes from their predecessors have important synthetic consequences. First, transmetalation cannot be used to install these ligands. Strong Ag–CAAC binding impedes carbene transfer even for neutral CAAC ligands,^33^ a problem exacerbated for anionic carbenes.^34^ Instead, we attempted a modified version of the original route to neutral CAAC catalysts, in which the iminium salt is deprotonated with strong base, and the resulting CAAC is added to **HI** (Scheme 1a, dashed arrow).^35,36^ Both steps were carried out at 60 °C, to maximize the THF-solubility of the CAAC-sulfonates. Extensive decomposition resulted, however, and the target catalysts were isolated in <15% yield. The low yields may be due in part to abstraction of the benzylidene ligand by the carbene (Scheme 1b, left),^37^ as suggested by NMR and mass spectrometric evidence for **1a**.

To impede benzylidene abstraction, we turned to a “back-door” synthetic strategy developed in parallel work.^38^ This involves installation of the CAAC ligand on the di-iodide analogue of **HI**,^39^ in which the bulky iodide ligands limit access of the free carbene to the [Ru]=CHAr carbon.^38^ Following CAAC ligation, the desired chloride complex can be safely generated via anion exchange. The success of this approach is shown in Scheme 1a (bold arrow). Installation of the sulfonated CAAC ligands on **HI-I**_**2**_ was complete within 2 h, affording the cyclohexyl **C1**_**S**_^**Cy**^ derivative in 93% yield after chromatography. The smaller **C1**_**S**_^**Me**^ analogue was isolated in lower yields (52%), consistent with more facile participation in side-reactions. In addition to attack on the benzylidene functionality, these include the recently uncovered borylation by the BF_4_^–^ counteranion (Scheme 1b, right).^40^ Formation of **1b** (Figure S9) accounts for the initially puzzling requirement for excess CAAC.

The final synthetic step, exchange of the iodide ligands for chloride, was carried out in methanol, in which the iodide complexes are fully soluble. Chlorination by AgCl proved more efficient than NaCl, a reflection of the very small dissociation constant for the AgI product.^41^ The limited solubility of AgCl in methanol retards exchange, but reaction was complete after 2 days (Tables S2, S3), affording the chloride complexes in >90% isolated yield. Somewhat unexpectedly, the new catalysts are soluble in CH_2_Cl_2_, as well as methanol, acetone, water and to some extent THF (see Table S4). As anticipated, the smaller catalyst shows higher water-solubility.

With the sulfonated CAAC catalysts in hand, our first priority was to examine their water-tolerance relative to **AM**. The stability of metathesis complexes is conventionally assessed by NMR analysis, via integration of the alkylidene signal against an internal standard over time. In D_2_O, such experiments are precluded by rapid exchange-averaging, which causes the benzylidene signals to broaden into the baseline. Electronic spectroscopy offers an invaluable alternative, particularly given the diagnostic colors of the precatalysts relative to their Ru(II) decomposition products. Prior spectrophotometric studies revealed the rapid degradation of **AM** and related catalysts in water,^19−22^ and the role of chloride ion in retarding decomposition. We examined the stability of the small CAAC complex **HC1**_**S**_^**Me**^ relative to **AM**, in the expectation that reduced size would correlate with increased vulnerability.^30c^

Consistent with the literature reports,^19,20^**AM-H**_**2**_**O** forms immediately on dissolving **AM** in water in the absence of NaCl. The aqua complex decays over 2 h at RT (Figure 2, left). Aquation of **HC1**_**S**_^**Me**^ under these conditions is likewise immediate, as judged from the observation of a single absorption band at 370 nm (Figure 2, right; cf. λ_max_ = 380 nm in 2 M NaCl_(aq)_; Figure S18). The aqua species is significantly more water-stable than its **AM** analogue, however, undergoing only 20% loss over 24 h at RT. The capacity of the anionic CAAC ligand to retard the decomposition cascade holds promise for aqueous metathesis, to which we now turn.

**Figure 2:**
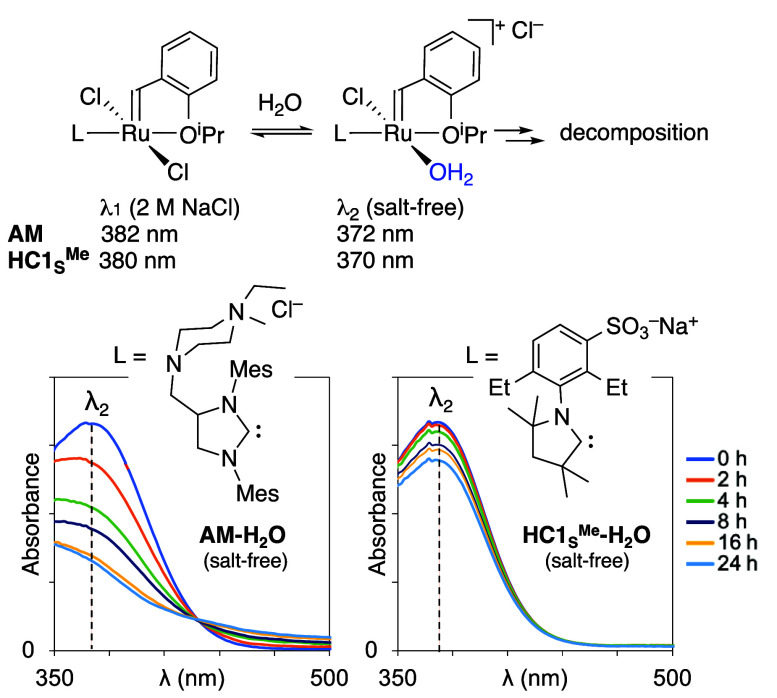
Aquation of **AM** and **HC1**_**S**_^**Me**^ in degassed water, and rapid ensuing degradation of **AM**.

To benchmark the performance of the new catalysts in water, we examined the ring-closing metathesis (RCM) of diol **2** (Figure 3a). The maximum turnover number (TON) reported for RCM of **2** in water is 650 ± 35 for an **HII** derivative embedded in a lipophilic streptavidin pocket,^42^ or 210 for **AM** in buffered water,^43^ at ca. 40 °C. In experiments with **HC1**_**S**_^**Me**^ and **AM**, we observed TONs of 640 and 420, respectively, albeit at a higher temperature (70 °C). The iodide catalyst **HC1**_**S**_^**Me**^-I_2_ and cyclohexyl catalyst **HC1**_**S**_^**Cy**^ were much less productive (TON 60 and 40, respectively; Figure 3a), probably due to their steric bulk, as well as poorer solvation. Of note, related NHC-iodide catalysts exhibit outstanding tolerance for trace water, delivering very high TONs despite reacting more slowly than the chloride analogues.^30d^ The much poorer performance seen for **HC1**_**S**_^**Me**^-I_2_ in bulk water reinforces the point that any factors that retard metathesis have a major negative impact in aqueous metathesis, where decomposition rates are significantly faster.

**Figure 3:**
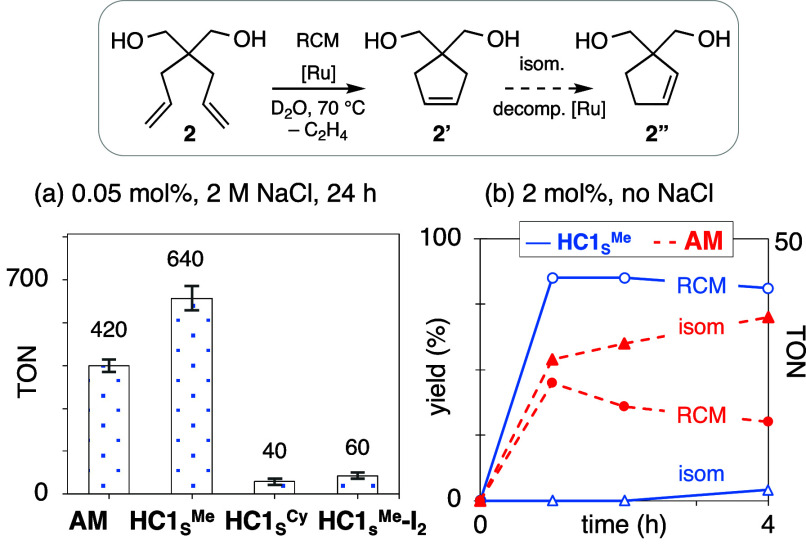
(a) RCM productivity of water-soluble catalysts (Table S5): TONs based on yield of **2′** at 24 h. No **2″** observed. (b) Reaction profile in the absence of NaCl, showing extensive isomerization for **AM**.

In the absence of NaCl, metathesis productivity and selectivity decline sharply, and catalyst loadings must be increased 40-fold to achieve high conversions of **2**. As shown in Figure 3b, **AM** is particularly affected. At 4 h, the yield of **2′** is only 30% (TON 15), the balance being isomerization product **2″**. Olefin isomerization is well established as *the* major side-reaction for metathesis of terminal olefins in organic solvents.^44^ It clearly remains a major problem in bulk water for NHC catalysts, notwithstanding the different decomposition pathways and Ru speciation.^23^ In striking contrast, the CAAC catalyst induces negligible isomerization in water.^45^ This has important implications for metathesis in chemical biology, where high excesses of the ruthenium species are required to drive metathesis.

While the TON of 640 for **HC1**_**S**_^**Me**^ in the presence of NaCl (Figure 3a) is excellent for metathesis in water, it is dramatically lower than the TON of >11,000 achieved by the same catalyst in RCM of diethyl diallylmalonate **3** in CH_2_Cl_2_ even at RT (Table 1, entry 1). Competitive binding of water and olefin may be an intrinsic limitation to metathesis productivity in water, by analogy to the competitive inhibition suggested for THF.^44^ Consistent with this hypothesis, yields in RCM of **2** are slightly higher in 1:1 D_2_O-^*t*^BuOH than neat D_2_O (Table S6).

**Table 1:**
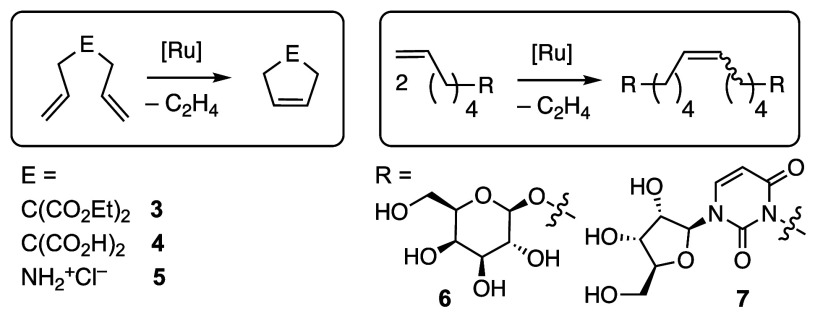
Performance of Sulfonate Catalysts in Metathesis^a^

entry	substrate	Ru cat	mol %	t (h)	yield (%)	TON
1	**3**^a^	**HC1**_**S**_^**Me**^	0.005	24	56	11,200
2	**4**	**HC1**_**S**_^**Me**^	0.5	24	35	70
			0.5	24	49^b^	98^b^
3	**5**	**HC1**_**S**_^**Me**^	0.1	24	8	80
			1	24	100	100
4	**6**	**HC1**_**S**_^**Me**^	1	24	100	100^c^
			0.1	24	51	510
5	**7**	**HC1**_**S**_^**Cy**^	0.1	4	70	700
			0.05	4	42	840
			1	4	100	100
		**HC1**_**S**_^**Me**^	0.1	4	100	1,000^d^
			0.05	4	76	1,520

a In CH_2_Cl_2_ (substrate **3**; RT); or D_2_O with 2 M NaCl (all others); at 70 °C except where noted. Yields within ±2% in replicate runs. Optimal performance requires inert atmosphere: see Table S5.

b At pH 3.

c Cf. 2% yield at RT.

d Cf. 9% yield after 24 h at RT with 1 mol % **HC1**_**S**_^**Me**^.

RCM of diallylmalonic acid **4** in D_2_O was also attempted (Table 1, entry 2). Yields are limited to ca. 35% at pH 7, perhaps owing to chelation of anionic carboxylate: they increase to 50% at pH 3.

The efficacy of the CAAC–NaCl combination in suppressing isomerization was examined more closely in experiments with diallylamine hydrochloride **5** (Table 1, entry 3). The latter substrate constitutes a highly sensitive probe of C=C migration, owing to the metathesis-inactivity of its N-vinylamine isomer. Cyclization of **5** was quantitative at 1 mol % **HC1**_**S**_^**M**^^e^, a catalyst loading 5-fold lower than that routinely required.^19^ At 0.1 mol %, RCM yields dropped to ca. 10%, but again, no isomerization was evident even after 24 h. The CAAC complex thus stands out in enabling *selective* metathesis in water.

A final set of experiments focused on metathesis of unprotected biomolecules, an area of tremendous opportunity, with few reports to date.^4−7,15,16^ Metathesis of protected carbohydrate substrates has enabled advances in applications ranging from antimicrobial therapeutics to tissue engineering.^46,47^ The Ramström group recently reported metathetical coupling of unprotected carbohydrates in neat water, without cosolvents or privileged coupling partners.^15^ Up to 81% dimerization of β-d-galactopyranoside **6** was described on use of 5 mol % of NHC catalyst **Ru-2**, when acid was used to suppress C=C migration.^48^ In comparison, **HC1**_**S**_^**Me**^ enables quantitative dimerization at neutral pH with 1 mol % Ru, or ca. 50% yield at 0.1 mol % (Table 1, entry 4). Given the susceptibility of these long-chain substrates to isomerization, and the limitations of NMR detection, we reanalyzed the higher-loading experiment by mass spectrometry. A small M–14 peak was evident, indicating ca. 5% isomerization prior to metathesis. In comparison, RCM of related mannosides by **Ru-2** in the absence of acid caused nearly 90% isomerization at 60 °C, with the desired dimer being formed in just 5% yield.^15^

Finally, motivated by the explosion in interest in oligonucleotide therapeutics,^49^ and the enhanced activity demonstrated for nucleoside dimers accessed via click chemistry,^50^ we examined metathetical coupling of uridine-tagged **7** (Table 1, entry 5). Prior examples of nucleoside metathesis employ protected glycosylamines in organic solvent, and proceed in low yields.^51^ Coupling of **7** is the first reported example of the metathesis of an unprotected nucleoside. Both **HC1**_**S**_^**Me**^ and **HC1**_**S**_^**Cy**^ dimerized **7** quantitatively, at loadings of 0.1 or 1 mol %, respectively; at 0.05 mol % **HC1**_**S**_^**Me**^, yields reached 76%. A limitation, however, is the need for elevated temperatures. At RT, <10% dimer is observed even at 1 mol % Ru, perhaps because the electron-withdrawing sulfonate ligand exacerbates the slow propagation characteristic of CAAC catalysts.^30b,52^ Nevertheless, the TON of 1,520 for nucleoside substrate **7** is the highest yet reported for metathesis of terminal olefins in water. The improvement over RCM of the simpler substrates **2** and **5** may reflect the extended “tether length” to the terminal olefin (the incipient site of Ru installation), which is beneficial in related contexts.^14,15^

Water-soluble ammonium catalysts for olefin metathesis, although long established, have seen little use for challenging reactions in chemical biology. The surprising preference for neutral, organic-soluble catalysts is driven by higher metathesis productivity, which outweighs the challenges in achieving phase homogeneity. The foregoing describes novel, negatively charged catalysts that are set to change this picture. A CAAC ligand bearing an anionic sulfonate tag improves water-tolerance, solubility, and metathesis performance. The small CAAC catalyst **HC1**_**S**_^**Me**^ delivers record productivity, at neutral pH, for coupling of unprotected carbohydrates and nucleosides. The TON of 1,520 for nucleoside dimerization is the highest yet reported for metathesis of terminal olefins in water. Importantly, isomerization is also negligible. In contrast, decomposed **AM** causes extensive isomerization, indicating that this unwanted side-reaction remains a threat for NHC catalysts even in aqueous media. The selectivity of the CAAC catalyst for metathesis in water thus represents a key additional asset. Redesign to enable ambient-temperature operation would expand opportunities in chemical biology. Such “next-generation” catalysts are now being pursued in our laboratories.

## Supporting Information Available

Experimental details, NMR and UV–vis spectra (PDF)
