# Supplementary material for: Anionic Olefin Metathesis Catalysts Enable Modification of Unprotected Biomolecules in Water
Source: ACS Catal. 2024 Jul 11;14(15):11147–52. doi: 10.1021/acscatal.4c02811 (PMC11301623; doi:10.1021/acscatal.4c02811)
Supplement: Supplementary file 1 — cs4c02811_si_001.pdf [file cs4c02811_si_001.pdf]

Supporting Information

Anionic Olefin Metathesis Catalysts Enable Modification of Unprotected Biomolecules in Water

Christian O. Blanco,[a] Richard Ramos Castellanos,[a] and Deryn E. Fogg*[a,b]

[a]Center for Catalysis Research & Innovation, and Department of Chemistry and Biomolecular Sciences, University of Ottawa, ON, Canada, K1N 6N5. [b]Department of Chemistry, University of Bergen, Allégaten 41, N-5007 Bergen, Norway

*Corresponding author: dfogg@uottawa.ca, deryn.fogg@uib.no

TABLE OF CONTENTS

S1. Experimental.......... S2

S1.1. General Procedures.......... S2

S1.2. Synthesis of Ligands and Catalysts.......... S3

S1.3. Synthesis of Novel Uridine Substrate and Metathesis Dimer.......... S8

S1.4. Catalytic Performance of Sulfonated Catalysts.......... S9

S1.5. Monitoring Catalyst Stability by UV-Vis Spectroscopy.......... S10

S2. NMR Spectra.......... S11

S3. References.......... S45

S1. Experimental.

S1.1. General Procedures. All reactions were carried out in an N_2-filled glovebox unless otherwise noted. HPLC-grade hexanes, THF, and CH_2Cl_2 were dried and degassed with a Glass Contour solvent purification system and stored under N_2 over 4 Å molecular sieves for at least 24 h prior to use. MeOH was distilled from CaH_2 under N_2 and stored as above. Metathesis catalysts **HI**[1] and **HI-I₂**,[2] CAAC salts[3] **C1^{Me}**•HBF_4, and **C1^{Ph}**•HBF_4, 2,2-diallylpropane-1,3-diol **2**,[4] 2,2-diallylmalonic acid **4**,[5] diallyl ammonium chloride **5**,[6] and olefin **6**[7] were prepared by literature methods. CD_3OD (Cambridge Isotopes, 99.5%), ^{t}BuOH (Sigma, 99.5%), MilliQ H_2O, and D_2O (Cambridge Isotopes, 99.5%) were freeze-pump-thaw degassed (4×) and stored under N_2 in the glovebox. Dimethyl sulfone (Me_2SO_2, 98%; internal standard for NMR analysis), diethyl diallylmalonate **3** (TCI, 98%), potassium trispyrazolyl borate (KTp; quenching agent;[8] Sigma, 98%), **C1^{Cy}**•HBF_4 and **AM** (the last two kindly provided as a gift by Apeiron Synthesis) were used as received. LiHMDS (Sigma, 97%) was recrystallized from hexanes and stored under N_2 in the glovebox at –35 °C. The purity of all catalysts was confirmed by ^{1}H NMR analysis prior to use. For accuracy in metathesis experiments, solid catalysts were weighed outside the glovebox using a microanalytical balance.

NMR spectra were recorded on Bruker Avance 300, 400, and 600 MHz NMR spectrometers at 25 ±0.5 °C. Chemical shifts (ppm) are referenced to the residual proton of the deuterated solvent for ^{1}H NMR spectra (CD_3OD: 3.31 ppm; $CDCl_3$: 7.26 ppm; DMSO-d_6: 2.50 ppm), for $^{13}C\{^1H\}$ NMR to the carbon atom of the deuterated solvent (CD_3OD: 49.00 ppm; $CDCl_3$: 77.16 ppm; DMSO-d_6: 39.52 ppm), for ^{19}F NMR spectra to external fluorobenzene at –164.9 ppm, and for ^{11}B NMR spectra to external BF_3•Et_2O at 0.00 ppm. Quantitative NMR experiments were used to quantify catalysis, using a standard delay time (D1) of 30 seconds. UV-vis spectra were measured with a Mettler-Toledo Easy UV spectrophotometer, pH with a Mettler-Toledo FiveEasy glass pH electrode (3.0 M KCl reference pH probe, calibrated using a set of 3 standardized buffer solutions: pH 4.00, 7.00, 11.00). Electrospray (ESI) mass spectra were acquired with a Micromass Q-TOF I Mass Spectrometer (Waters) on 30 µg/mL MeCN or MeOH solutions prepared under N_2, via injection of a 1 mL volume at 50 µL/min and nebulization with N_2 (70 psi) at 200 ºC, using capillary and cone voltages of 3.5 and 40 kV, respectively, and a source temperature of 100 ºC.

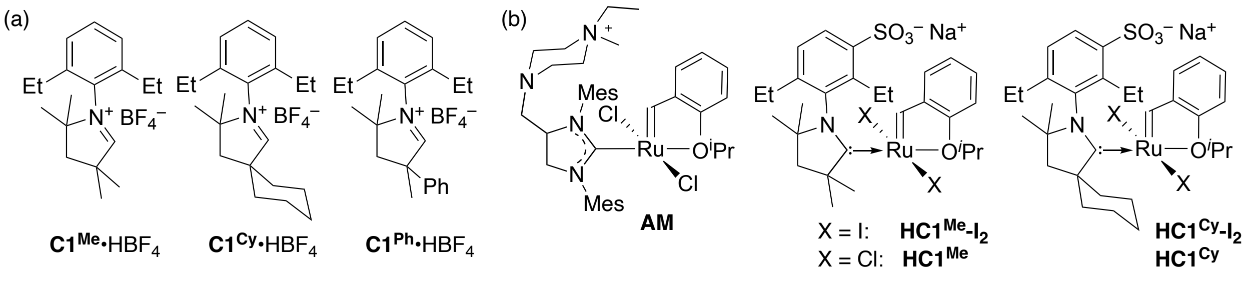

Chart 1. (a) CAAC iminium salts employed as precursors to sulfonated CAACS. (b) Catalysts employed.

S1.2. Synthesis of Ligands and Catalysts.

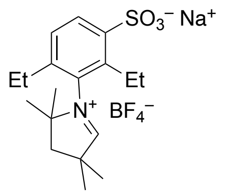

C1s^{Me}•HBF$_4$

S1.2.1. Synthesis of C1s^{Me}•HBF$_4$. In a well-ventilated fumehood, a 50 mL round-bottom flask was charged with 18% fuming sulfuric acid (4 mL) and concentrated sulfuric acid (1 mL) in an ice bath. The mixture was stirred for 5 min, after which white solid **C1^{Me}**•HBF$_4$ (1.00 g, 2.90 mmol) was added in small portions over 20 min, turning into a red solution after the first 2 min. The red solution was allowed to warm to RT, stirred for 10 min, then slowly poured over ice in a 500 mL round-bottom flask bedded in an ice-bath. The resulting white suspension was neutralized with saturated NaOH to pH 7. The water was evaporated under vacuum, and the white residue was taken up in dry methanol and filtered to remove Na salts. Evaporation of the filtrate afforded a white solid, which was dried in vacuo for 2 days. Yield of **C1s^{Me}**•HBF$_4$: 1.01 g, 2.25 mmol (78%).

^{1}H NMR (600 MHz, D_2O): δ 9.40 (s, 1H, C*H*N), 8.11 (d, $^3J_{HH}$ = 8.4 Hz, 1H, NAr), 7.58 (d, $^3J_{HH}$ = 8.4 Hz, 1H, NAr), 3.57 (m, 1H, C*H*HMe; diastereotopic), 2.73 (m, 1H, C*H*HMe; diastereotopic), 2.53 (s, 2H, CH_2; overlaps with C*H*HMe), 2.50 (m, 3H, C*H*HMe; diastereotopic, overlaps with CH_2), 1.66 (s, 3H, CH_3), 1.65 (s, 3H, CH_3), 1.58 (s, 3H, CH_3), 1.54 (s, 3H, CH_3), 1.24 (t, $^3J_{HH}$ = 7.8 Hz, 3H, CH_2CH_3), 1.09 (t, $^3J_{HH}$ = 7.1 Hz, 3H, CH_2CH_3). For fully-assigned ^{1}H NMR spectrum, see Figure S1. ^{13}C{^{1}H} NMR (150 MHz, D_2O): δ 192.0 143.9, 140.6, 138.7, 132.0, 130.4, 128.0, 85.9, 48.9 47.9, 47.8, 27.8, 27.0, 25.6, 24.9, 23.1, 15.4, 14.3. ^{19}F{^{1}H} NMR (150 MHz, D_2O): δ –150.6 (s).
ESI-MS (MeOH): Calc'd for $C_{18}H_{27}NO_3SH^+$ ([M–BF$_4$]$^+$), *m/z* 338.1790. Found: *m/z* 338.1740.

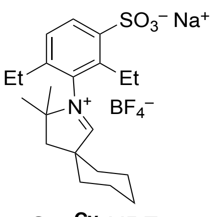

C1s^{Cy}•HBF$_4$

S1.2.2. Synthesis of C1s^{Cy}•HBF$_4$. As above, with **C1^{Cy}**•HBF$_4$ (1.01 g, 2.62 mmol). Yield of white crystalline **C1s^{Cy}**•HBF$_4$: 1.04 g, 2.13 mmol (82%).
^{1}H NMR (600 MHz, D_2O): δ 9.49 (s, 1H, C*H*N$^+$), 8.13 (d, $^3J_{HH}$ = 8.2 Hz, 1H, NAr), 7.59 (d, $^3J_{HH}$ = 8.2 Hz, 1H, NAr), 3.57 (m, 1H, C*H*HMe; diastereotopic), 2.73 (m, 1H, C*H*HMe; diastereotopic), 2.61(m, 1H, C*H*HMe; diastereotopic), 2.55 (d, $^3J_{HH}$ = 7.7 Hz, 2H, CH_2; overlaps with C*H*HMe), 2.52 (m, 1H, C*H*HMe; diastereotopic, overlaps with CH_2), 2.11–1.48 (m, 10H, Cy), 1.60 (s, 3H, CH_3), 1.54 (s, 3H, CH_3), 1.24 (t, $^3J_{HH}$ = 7.2 Hz, 3H, CH_2CH_3), 1.09 (t, $^3J_{HH}$ = 7.2 Hz, 3H, CH_2CH_3). For fully-assigned ^{1}H NMR spectrum, see Figure S2. ^{13}C{^{1}H} NMR (150 MHz, D_2O): δ 191.4, 143.9, 140.6, 138.7, 132.2, 130.4, 128.0, 85.1, 53.1, 48.9, 45.3, 34.5, 33.6, 28.3, 27.4, 25.0, 24.3, 23.1, 21.2(5), 25.1(8) 15.4, 14.3. ^{19}F{^{1}H} NMR (150 MHz, D_2O): δ –150.5 (s).
ESI-MS (MeOH): Calc'd for $C_{21}H_{31}NO_3SNa^+$ ([M–BF$_4$]$^+$), *m/z* 400.1917. Found: *m/z* 400.1919.

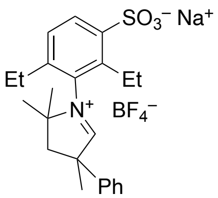

C1s^{Ph}•HBF$_4$

S1.2.3. Attempted synthesis of monosulfonated C1s^{Ph}•HBF$_4$. Carrying out this reaction as for **C1s^{Me}**•HBF$_4$, with **C1^{Ph}**•HBF$_4$ (1.00 g, 2.46 mmol) resulted in formation of a 1:1 mixture of two polysulfonated products, and was therefore not pursued further. A ^{1}H NMR spectrum showing the product mixture is provided in Figure S3.

S1.2.4. Failed synthesis of RuCl$_2$(H$_2$IMes-SO$_3$Na)(=CHAr) (HII-SO$_3^-$ Na$^+$) via direct sulfonation. In a well-ventilated fumehood, a 50 mL Schlenk flask connected to the Schlenk line was charged with 18% fuming sulfuric acid (1.00 mL) and concentrated sulfuric acid (0.25 mL) in an ice bath. The mixture was stirred for 5 min, after which green solid **HII** (50 mg, 0.080 mmol)

was added all at once, turning immediately into a black solution. The solution was allowed to warm to RT and was slowly poured into another Schlenk flask under N_2 bedded in an ice-bath. The resulting dark-brown suspension was neutralized with saturated NaOH to pH 5. The water was evaporated under vacuum, and the brown and white residue was taken up in dry methanol inside of a glovebox and filtered to remove Na salts. Evaporation of the filtrate afforded a brown solid. No product signals were observed by ^{1}H NMR analysis: Figure S4.

S1.2.5. Synthesis of RuI_2($C1_S^{Me}$)(=CHAr), $HC1_S^{Me}$-I_2. A white suspension of **$C1_S^{Me}$•HBF_4** (800 mg, 1.78 mmol, 2 equiv) and LiHMDS (297 mg, 1.78 mmol, 2.0 equiv) in 10 mL THF was transferred to a thermostatted oil bath set at 60 °C, and stirred for 10 min. The solution turned yellow within 5 min, and a homogeneous solution formed within 10 min. (In comparison, a heterogeneous mixture was present even after 4 h at RT). Dropwise addition to a green solution of **HI-I_2** (700 mg, 0.893 mmol) in THF (10 mL) caused immediate formation of a green-yellow suspension. The reaction was stirred at 60 °C, with periodic removal of aliquots for ^{31}P NMR analysis (THF). Once no signal for **HI-I_2** remained (2 h), the solvent was evaporated under reduced pressure to give a dark green oil. Chromatography on silica gel in air (99:1 CH_2Cl_2:MeOH), isolation of the green band, and evaporation of solvent gave a green solid, which was washed with benzene (3×2 mL) to remove a yellow impurity and dried. Yield of green **$HC1_S^{Me}$-I_2**: 502 mg, 0.463 mmol (52%).

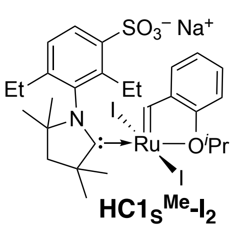

$HC1_S^{Me}$-I_2

^{1}H NMR (300 MHz, CD_3OD): δ 15.64 (s, 1H, [Ru]=C*H*), 8.26 (d, $^3J_{HH}$ = 9 Hz, 1H, NAr), 7.65 (t, $^3J_{HH}$ = 8 Hz, 1H, Ar C*H*), 7.53 (d, $^3J_{HH}$ = 9 Hz, 1H, NAr), 7.16 (d, $^3J_{HH}$ = 8 Hz, 1H, Ar C*H*), 7.00 (m, 1H, Ar C*H*), 6.86 (m, 1H, Ar C*H*), 5.35 (sept, $^3J_{HH}$ = 5 Hz, 1H, C*H*Me_2), 3.31 (m, C*H*HMe + CAAC backbone CH_2; overlaps with solvent peak), 3.07 (m, 2H, C*H*HMe; diastereotopic), 2.61 (m, 1H, C*H*HMe; diastereotopic), 2.20 (s, 3H, CH_3), 1.90 (d, $^3J_{HH}$ = 5 Hz, 3H, ^{i}Pr CH_3), 1.32 (s, 3H, CH_3), 1.16 (s, 3H, CH_3), 1.06 (t, $^3J_{HH}$ = 8 Hz, 3H, CH_2CH_3), 0.90 (t, $^3J_{HH}$ = 8 Hz, 3H, CH_2CH_3). For fully-assigned ^{1}H NMR spectrum, see Figure S5.

^{13}C{^{1}H} NMR (125 MHz, CD_3OD): δ 290.3 ([Ru]=*C*H; not observed: detected by ^{1}H-^{13}C HSQC), 271.3 (CAAC *C*:), 153.4, 145.8, 143.7, 143.2, 142.7, 138.5, 131.0, 129.7, 125.7, 123.8, 121.4, 113.7, 79.6, 75.6, 55.0, 50.6, 48.2, 32.0, 31.9, 28.4, 27.4, 26.1, 24.4, 21.8, 17.0, 13.8.

ESI-MS (MeCN): Calc'd for $C_{28}H_{38}I_2NO_4SRu^-$ ([M–Na]$^-$), *m/z* 839.9660. Found: *m/z* 839.9662.

S1.2.6. Synthesis of RuI_2($C1_S^{Cy}$)(=CHAr), $HC1_S^{Cy}$-I_2. As for **$HC1_S^{Me}$-I_2**, using **$C1_S^{Cy}$•HBF_4** (311 mg, 0.638 mmol, 2 equiv), LiHMDS (106 mg, 0.638 mmol, 2.0 equiv) and **HI-I_2** (250 mg, 0.310 mmol). Yield of green **$HC1_S^{Cy}$-I_2**: 271 mg, 0.294 mmol (93%).

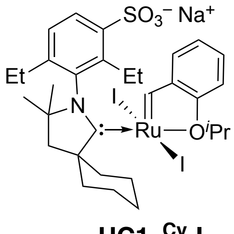

$HC1_S^{Cy}$-I_2

^{1}H NMR (300 MHz, CD_3OD): δ 15.82 (s, 1H, [Ru]=C*H*), 8.26 (d, $^3J_{HH}$ = 8 Hz, 1H, NAr), 7.65 (m, 1H, Ar C*H*), 7.51 (d, $^3J_{HH}$ = 8 Hz, 1H, NAr), 7.16 (d, $^3J_{HH}$ = 9 Hz, 1H, Ar C*H*), 7.00 (m, 1H, Ar C*H*), 6.86 (m, 1H, Ar C*H*), 5.32 (sept, $^3J_{HH}$ = 6 Hz, 1H, C*H*Me_2), 3.35 (s, 2H, CAAC backbone CH_2), 3.13 (m, 3H, 3 diastereotopic C*H*HMe protons overlap), 2.55 (m, 2H, Cy + C*H*HMe; diastereotopic), 2.76–1.42 (m, 9H, Cy), 1.88 (br s, 6H, ^{i}Pr CH_3), 1.31 (s, 4H, CH_3 + Cy), 1.14 (s, 3H, CH_3), 1.05 (t, $^3J_{HH}$ = 8 Hz, 3H, CH_2CH_3), 0.98 (t, $^3J_{HH}$ = 8 Hz, 3H, CH_2CH_3), 0.89 (m, 1H, Cy). For fully-assigned ^{1}H NMR spectrum, see Figure S6.

$^{13}C\{^1H\}$ NMR (150 MHz, CD_3OD): δ 293.0 ([Ru]=*C*H), 273.3 (CAAC *C*:), 154.8, 147.2, 145.1, 144.8, 144.2, 140.0, 132.5, 131.0, 127.0, 125.3, 122.7, 115.1, 80.9, 76.9, 63.0, 54.8, 44.5, 39.8, 39.0, 30.6, 29.3, 27.4, 26.7, 25.9, 24.5, 24.4, 23.3, 18.4, 15.2.

ESI-MS (MeCN): Calc'd for $C_{31}H_{42}I_2NO_4SRu^-$ ([M–Na]$^{-ß}$), *m/z* 879.9973. Found: *m/z* 879.9983.

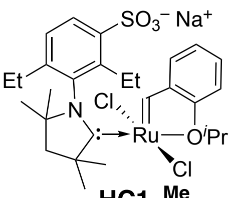

HC1s^{Me}

S1.2.7. Failed synthesis of RuCl$_2$(C1s^{Me})(=CHAr) (HC1s^{Me}) via attemped ligand exchange with HI. As for the successful synthesis of **HC1s^{Me}-I$_2$** above, but using **C1s^{Me}•**HBF_4 (134 mg, 0.28 mmol, 2 equiv), LiHMDS (48 mg, 0.28 mmol, 2.0 equiv) and **HI** (100 mg, 0.166 mmol). Yield of isolated impure green-yellow **HC1s^{Me}**: 8 mg, 0.01 mmol (7%). For ^1H NMR spectrum of this material, see Figure S7. NMR analysis of the crude reaction mixture (Figure S8) indicated the presence of benzyl derivative **1a** and trifluoroborane adduct **C1s^{Me}**-BF_3 (**1b**).

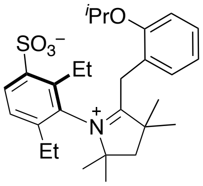

1a

Key signals for zwitterionic **1a**, observed in situ: ^1H-^{13}C HMBC (CD_3OD): 3.81 (s, CH_2). Correlations: 131.4 (aromatic *C*H), 140.1 (4° aromatic *C*), 95.3 (4° aliphatic *C*), 154.5 ppm (4° iminium *C*). ESI-MS (MeOH): Calc'd for $C_{28}H_{39}NO_4SNa^+$ ($[M+Na]^+$), *m/z* 508.2492. Found: *m/z* 508.2471.

Key signals for **C1s^{Me}**-BF_3 **(1b)**, observed in situ: see Table S1 and Figure S9.

ESI-MS (MeOH): Calc'd for $C_{18}H_{23}BF_3O_3NS^-$ ($[M–Na]^-$), *m/z* 404.1684. Found: *m/z* 404.1670.

Table S1. Key NMR signals for **1b** (**C1s^{Me}**-BF_3): comparison with known[9] values for **C1^{Me}**-BF_3.

**Compound**	**Solvent**	**^{19}F (282 MHz)**	**$^{11}B\{^1H\}$ (96 MHz)**
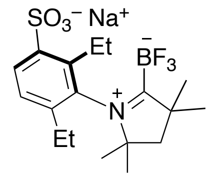 **C1s^{Me}**-BF_3 (**1b**)	CD_3OD	–139.5 ppm (q, $^1J_{F\text{-}B}$ = 39 Hz)	–0.52 ppm (q, $^1J_{B\text{-}F}$ = 41 Hz) (overlaps with BF_4^-)
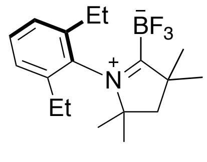 **C1^{Me}**-BF_3	C_6D_6	–139.7 (q, $^1J_{F\text{-}B}$ = 36 Hz)	0.06 (q, $^1J_{B\text{-}F}$ = 36 Hz) (overlaps with BF_4^-)

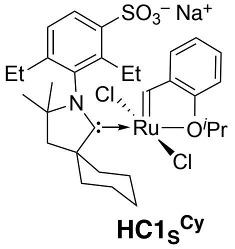

HC1s^{Cy}

S1.2.8. Failed synthesis of RuCl$_2$(C1s^{Cy})(=CHAr) (HC1s^{Cy}) via reaction with HI. The reaction was carried out as for **HC1s^{Me}-I$_2$**, but using **C1s^{Cy}•**HBF_4 (179 mg, 0.366 mmol, 2 equiv) and LiHMDS (61 mg, 0.366 mmol, 2.0 equiv) and adding the mixture to **HI** (110 mg, 0.183 mmol). Yield of impure brown-yellow solid containing **HC1s^{Cy}**: 13 mg, 0.017 mmol (13%). For ^1H NMR spectrum, see Figure S10.

S1.2.9. Synthesis of RuCl$_2$(C1s^{Me})(=CHAr), HC1s ^{Me}. Solid AgCl (82 mg, 0.57 mmol, 5 equiv) was added to a yellow-green solution of **HC1s ^{Me}-I$_2$** (100 mg, 0.115 mmol) in MeOH. ^{1}H NMR analysis after stirring at RT for 24 h revealed 10% of the mixed-halide species **HC1s^{Me}-I** (Table S2), which disappeared over a further 24 h. The green suspension was filtered through Celite to remove Ag salts. The product was washed through with CH_2Cl_2 (3 × 5 mL), the combined filtrate was concentrated to a minimum volume, and hexanes was added. The precipitate was filtered off, washed with cold hexanes (3×1 mL), and dried under vacuum. Yield of green **HC1s^{Me}**: 76 mg, 0.11 mmol (92%).

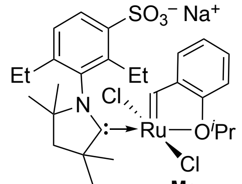

HC1s^{Me}

^{1}H NMR (300 MHz, CD_3OD): δ 16.99 (s, 1H, [Ru]=C*H*), 8.31 (d, $^3J_{HH}$ = 8 Hz, 1H, NAr), 7.61 (dd, $^3J_{HH}$ = 8 Hz, $^4J_{HH}$ = 2.1 Hz, 1H, Ar C*H*), 7.52 (d, $^3J_{HH}$ = 8 Hz, 1H, NAr), 7.16 (d, $^3J_{HH}$ = 8 Hz, 1H, Ar C*H*), 6.93 (m, 2H, Ar C*H*), 5.25 (sept, $^3J_{HH}$ = 5 Hz, 1H, C*H*Me$_2$), 3.45 (m, 1H, C*H*HMe; diastereotopic), 3.31 (detected by ^{1}H-^{13}C HMBC, CAAC backbone C*H*$_2$, overlaps with residual CHD_2OD), 2.86 (m, 1H, C*H*HMe; diastereotopic), 2.69 (m, 1H, C*H*HMe; diastereotopic), 2.47 (m, 1H, C*H*HMe; diastereotopic), 2.19 (s, 3H, C*H*$_3$), 1.97 (s, 3H, C*H*$_3$), 1.74 (d, $^3J_{HH}$ = 5 Hz, 6H, ^{i}Pr C*H*$_3$), 1.36 (s, 3H, C*H*$_3$), 1.13 (s, 3H, C*H*$_3$), 0.94 (t, $^3J_{HH}$ = 7 Hz, 3H, CH$_2$C*H*$_3$), 0.82 (t, $^3J_{HH}$ = 7 Hz, 3H, CH$_2$C*H*$_3$). For fully-assigned ^{1}H NMR spectrum, see Figure S11.

^{13}C{^{1}H} NMR (125 MHz, CD_3OD): δ 295.2 ([Ru]=*C*H; not observed: detected by ^{1}H-^{13}C HSQC) 268.3 (CAAC *C*:), 154.3, 147.1, 145.0, 144.6, 143.8, 140.0, 132.1, 130.9, 127.2, 124.6, 123.1, 114.5, 80.8, 76.3, 57.6, 52.5, 30.1, 29.8, 29.2, 28.4, 26.7, 25.2, 22.4, 22.4, 16.1, 14.7

ESI-MS (MeCN): Calc'd for $C_{28}H_{38}Cl_2NO_4SRu^-$ ([M–Na]$^-$), *m/z* 656.0944. Found: *m/z* 656.0889.

Table S2. Chemical shifts and product distribution in halide exchange with **HC1s^{Me}**.

Complex	δ (ppm)	Proportion	
		At 24 h	At 48 h
**HC1s^{Me}**	16.99	90%	100%
**HC1s^{Me}-I**	16.37, 16.33 (rotamers)	10%	0%
**HC1s^{Me} -I$_2$**	15.63	0%	0%

S1.2.10. Attempted synthesis of RuCl$_2$(C1s^{Me})(=CHAr), HC1s^{Me} via salt exchange with NaCl. To a yellow-green solution of **HC1s^{Me}-I$_2$** (50 mg, 0.058 mmol) in MeOH (5 mL) was added NaCl (678 mg, 11.6 mmol, 200 equiv) an let stir at RT. After 24 h, ^{1}H NMR analysis (see Figure S12) showed 6% starting material and 31% of the mixed-halide species **HC1s^{Me}-I**. The suspension was stirred for an additional 24 h, after which 20% **HC1s^{Me}-I** and 3% **HC1s^{Me}-I$_2$** remained. The suspension was filtered off and subjected to a second round of NaCl treatment for 48 h. ^{1}H NMR analysis showed 7% **HC1s^{Me}-I** remaining.

S1.2.11. Synthesis of RuCl$_2$(C1s^{Cy})(=CHAr), HC1s^{Cy}. As for **HC1s^{Me}**, using **HC1s^{Cy}**-I$_2$ (100 mg, 0.111 mmol), AgCl (79 mg, 0.55 mmol, 5.0 equiv) in MeOH (5 mL). Yield of green **HC1s^{Cy}**: 72 mg, 0.10 mmol (90%). Table S3 shows chemical shifts and yields of relevant species.

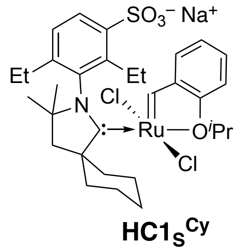

HC1s^{Cy}

^{1}H NMR (300 MHz, CD_3OD): δ 17.12 (s, 1H, [Ru]=C*H*), 8.32 (d, $^3J_{HH}$ = 8 Hz, 1H, NAr), 7.62 (dd, $^3J_{HH}$ = 8 Hz, $^4J_{HH}$ = 2 Hz, 1H, Ar C*H*), 7.53 (d, $^3J_{HH}$ = 8 Hz, 1H, NAr), 7.15 (d, $^3J_{HH}$ = 8 Hz, 1H, Ar C*H*), 6.96 (m, 2H, Ar C*H*), 5.24 (sept, $^3J_{HH}$ = 6 Hz, 1H, C*H*Me$_2$), 3.44, (m, 1H, C*H*HMe; diastereotopic), 3.31 (detected by ^{1}H-^{13}C HMBC, CAAC backbone C*H*$_2$, overlaps with CHD_2OD), 2.86 (m, 1H, C*H*HMe; diastereotopic),

2.69 (m, 1H, C*H*HMe; diastereotopic), 2.47 (m, 1H, C*H*HMe; diastereotopic), 2.22–1.24 (m, 9H, Cy), 1.76 (d, $^3J_{HH}$ = 6 Hz, 6H, , ^{i}Pr CH_3; overlaps with Cy), 1.35 (s, 3H, CH_3; overlaps with Cy), 1.15 (s, 3H, CH_3), 0.95 (t, $^3J_{HH}$ = 7 Hz, 3H, CH_2CH_3), 0.82 (t, $^3J_{HH}$ = 7 Hz, 3H, CH_2CH_3). For fully-assigned ^{1}H NMR spectrum, see Figure S13.

^{13}C{^{1}H} NMR (150 MHz, CD_3OD): δ 297.6 ([Ru]=*C*H), 268.1 (CAAC carbene), 154.3, 147.1, 144.9, 144.7, 143.9, 139.9, 132.2, 130.9, 127.2, 124.7, 123.1, 114.6, 80.8, 76.2, 63.8, 54.8, 49.0, 38.2, 34.4, 30.4, 28.9, 26.8, 26.7, 25.2, 24.3, 23.7, 22.5, 16.1, 14.7.

ESI-MS (MeCN): Calc'd for $C_{31}H_{42}Cl_2NO_4SRu^-$ ([M–Na]$^-$), *m/z* 695.1265. Found: *m/z* 695.1270.

Table S3. Chemical shifts and speciation in halide exchange with **HC1s^{Cy}**.

Complex	δ (ppm)	Proportion	
		At 24 h	At 48 h
**HC1s^{Cy}**	17.12	87%	100%
**HC1s^{Cy}-I**	16.52, 16.47 (rotamers)	13%	0%
**HC1s^{Cy}-I$_2$**	15.83	0	0%

S1.2.12. Determining the solubility of the sulfonated catalysts in various solvents. To a 4 mL vial charged with 20 mg solid catalyst, solvent was added via gas-tight syringe in 100 µL increments. The vial was shaken after every portion of solvent was added. The volume was recorded when homogeneity was achieved. Table S4 shows the solubility data.

Table S4. Solubility of the sulfonated catalysts in mg/mL.

Catalyst	Water	MeOH	THF	CH_2Cl_2	Hexanes
**HC1s^{Me}-I$_2$**	5	>100	40	>100	–
**HC1s^{Cy}-I$_2$**	2	>100	50	>100	–
**HC1s^{Me}**	17	>100	33	>100	–
**HC1s^{Cy}**	9	>100	40	>100	–

S1.3. Synthesis of Novel Uridine Substrate 7 and Metathesis Dimer 7'

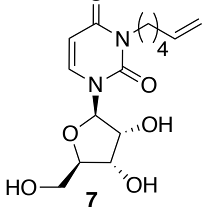
7

S1.3.1. Synthesis of hex-1-enyl-tagged uridine 7. A 25 mL high-pressure vessel was charged with uridine (2.00 g, 8.19 mmol), hex-5-en-1-yl methanesulfonate (1.60 g, 9.01 mmol, 1.1 equiv), K_2CO_3 (2.30 g, 16.4 mmol, 2.0 equiv), and DMF (10 mL) in air, capped and stirred at 80 °C in an oil bath for 24 h. The salts were then filtered off, and the product washed through with EtOAc (2×30 mL). After adding water (2×20 mL), the aqueous layer was extracted with EtOAc (2×30 mL). The combined organic layer was then washed with water (20 mL), dried ($NaSO_4$) and dried under vacuum at 50 °C for a day. The resulting white solid was purified by column chromatography (silica gel, MeOH/EtOAc 5:95) and recrystallized from boiling EtOAc, with hexanes as counter-solvent. Yield of white **7**: 1.51 g, 4.50 mmol (55%).

^{1}H NMR (600 MHz, $CDCl_3$): 7.63 (d, $^3J_{HH}$ = 8.2 Hz, 1H, =C*H*N), 5.78 (d, $^3J_{HH}$ = 8.0 Hz,1H, C*H*=CH_2, overlaps with C*H*C=O), 5.76 (d, $^3J_{HH}$ = 7.6 Hz, 1H, C*H*C=O, overlaps with C*H*=CH_2), 5.67 (d, $^3J_{HH}$ = 4.5 Hz, 1H, C*H*N), 5.03–4.91 (m, 2H, =CH_2), 4.38 (m, 1 H, C*H*OH_C), 4.35 (m, 1H, C*H*OH_B), 4.21 (m, 1H, OC*H*CH_2), 4.06 (d, $^3J_{HH}$ = 3.0 Hz, 1H, OH_C), 3.97 (m, 1H, C*H*HOH_A), 3.91 (dd, $^3J_{HH}$ = 7.8, Hz, $^4J_{HH}$ = 3.2 Hz, 2H, NCH_2), 3.90 (m, 1H, CH*H*OH_A), 3.22 (d, $^3J_{HH}$ = 4.0 Hz, 1H, OH_B), 2.62 (br s, 1H, OH_A), 2.08 (m, 2H, CH_2CH=), 1.62 (q, $^3J_{HH}$ = 8.1 Hz, 2H, NCH_2CH_2), 1.42 (q, $^3J_{HH}$ = 7.6 Hz, 2H, CH_2CH_2CH=). For fully-assigned ^{1}H NMR spectrum, see Figure S14.
^{13}C{^{1}H} NMR (150 MHz, $CDCl_3$): δ 162.7, 151.9, 138.8, 138.5, 114.9, 102.0, 93.7, 85.9, 75.3, 71.0, 62.2, 41.3, 33.5, 27.1, 26.3.
ESI-MS (MeOH): Calc'd for $C_{15}H_{22}N_2O_6Na^+$ ($[M+Na]^+$), *m/z* 349.1478. Found: *m/z* 349.1381.

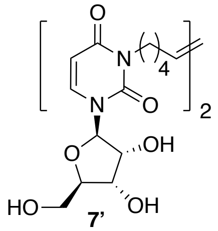
7'

S1.3.2. Synthesis and characterization of uridine dimer 7'. To a colourless solution of **7** (100 mg, 0.306 mmol, 100 equiv) in CH_2Cl_2 (5 mL) was added solid green **nGC1^{Ph}** (10 mg, 0.15 mmol, 5 mol%). The resulting red suspension was stirred at RT for 24 h. After cooling the suspension to RT, the solvent was decanted, and the pink residue was washed with CH_2Cl_2 (3×2 mL) to yield a white solid (a mixture of product with 6% starting material) which was purified by chromatography on silica gel (MeOH/EtOAc 5:95). Yield of white **7'**: 35 mg, 0.11 mmol (37%).

^{1}H NMR (600 MHz, DMSO-d_6): 7.95 (d, $^3J_{HH}$ = 8.1 Hz, 2H, =C*H*N), 5.80 (d, $^3J_{HH}$ = 5.0 Hz, 2H, C*H*N), 5.75 (d, $^3J_{HH}$ = 8.1 Hz, 2H, C*H*C=O), 5.38 (m, 1.2H, *E*-C*H*=), 5.33 (m, 0.4H, *Z*-C*H*=), 4.02 (m, 2H, C*H*OH_C), 3.97 (m, 2H, OC*H*CH_2), 3.85 (m, 2H, C*H*OH_B), 3.77 (m, 4H, NCH_2), 3.64 (m, 2H, C*H*HOH_A), 3.56 (m, 2H CH*H*OH_A), 1.98 (m, 4H, CH_2CH=), 1.50 (m, 4H, NCH_2CH_2), 1.29 (m, 4H, CH_2CH_2CH=). For fully-assigned ^{1}H NMR spectrum, see Figure S15.
^{13}C{^{1}H} NMR (150 MHz, DMSO-d_6): δ 161.9, 150.7, 139.1, 130.0, 129.6, 100.9, 88.8, 84.8, 73.7, 69.6, 60.6, 31.7, 26.6, 26.4.

ESI-MS (MeOH): Calc'd for $C_{28}H_{40}N_4O_{12}Na^+$ ($[M+Na]^+$), *m/z* 647.2540. Found: *m/z* 647.2559.

S1.4. Catalytic Performance of Sulfonated Catalysts.

S1.4.1. Representative RCM reaction with diene 2. Diene **2** (16 mg, 0.10 mmol), NaCl (117 mg, 0.4 mmol, 20 equiv), and dimethyl sulfone, Me_2SO_2 (9 mg, 0.10 mmol, 1 equiv; internal standard) were dissolved in 0.92 mL D_2O; final concentration 100 mM **2**. A 50 μL aliquot was removed for NMR analysis to establish the initial ratio of **2**:Me_2SO_2. To the stirred solution was added 0.1 mol% **HC1s^{Me}** (14 μL of a stock solution of 10.1 mg **HC1s^{Me}** in 2.00 mL D_2O). Aliquots were removed periodically, quenched with KTp in THF (10 mg/mL; 10 equiv vs starting Ru) and analyzed (NMR). Table S5 shows conversions of **2** and yields of **2'** (from the 2H olefinic signal for **2'** at 5.73 ppm; Figure S16, assigned by analogy to the reported signal in $CDCl_3$ at 5.65 ppm).[10]

Table S5. Yields, conversions, and TONs in RCM of diol **2** by water-soluble catalysts.

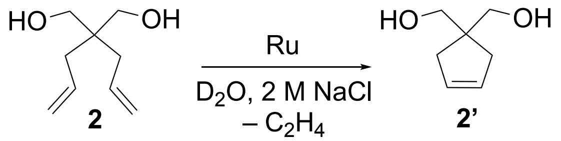

Catalyst	mol%	T (°C)	Atmosphere	Conversion (%)	Yield 2' (%)	TON
**HC1s^{Me}-I$_2$**	0.05	70	N_2	3	3	60
**HC1s^{Cy}**	0.05	70	N_2	2	2	40
**HC1s^{Me}**	0.05	70	N_2	32	32	640
**HC1s^{Me}**	0.05	70	Air	23	23	460
**AM**	0.05	70	N_2	22	22	420
**HC1s^{Me}**	0.5	70	N_2	100	100	200
**HC1s^{Me}**	0.5	70	Air	95	95	190
**HC1s^{Me}**	2[b]	70	N_2	85	80	40
**AM**	2[b]	70	N_2	100	30	15
**HC1s^{Me}**	0.05	RT	N_2	91	9	180

[a]Numerical data for Fig. 3 in main text. Agreement in replicate run averages ±2%. [b]At 4 h, 0 NaCl.

S1.4.2. RCM of 2 in ^{t}BuOH:D_2O. As above, in 0.92 mL ^{t}BuOH:D_2O (1:1). ^{t}BuOH was removed under vacuum prior to NMR analysis. Yields and conversions appear in Table S6.

Table S6. Yields, conversions, and TONs for RCM of **2** in D_2O vs D_2O-^{t}BuOH.[a]

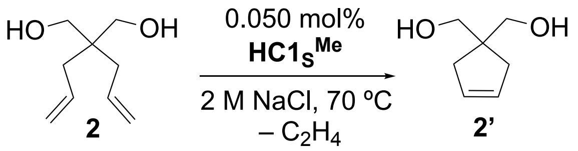

Solvent	[NaCl]	Conversion of 2 (%)	Yield of 2' (%)	TON
D_2O	0 M	99	0.5	10
1:1 D_2O:^{t}BuOH	0	6	6	120
D_2O	2.0	32	32	640
1:1 D_2O:^{t}BuOH	2.0	41	37	740

[a]Agreement in replicate run averages ±2%. Phase separation occurs with added NaCl.

S1.4.3. Representative procedure for metathesis dimerization (exemplified with known[7] 6'). β-D-galactopyranoside **6** (13 mg, 0.05 mmol), NaCl (58 mg, 1.0 mmol, 20 equiv), and dimethyl sulfone (Me_2SO_2; 5 mg, 0.10 mmol, 1 equiv, internal standard) were dissolved in 0.93 mL D_2O; final concentration 50 mM **6**. A 50 µL aliquot was removed for NMR analysis to establish the starting ratio of **6** vs Me_2SO_2. To the stirred solution was added **HC1s^{Me}** (68 µL of a stock solution of 10.0 mg **HC1s^{Me}** in 2.00 mL D_2O) to give a catalyst loading of 1 mol%. Aliquots were removed periodically, quenched with KTp in THF (10 mg/mL; 10 equiv vs starting Ru) and analyzed (NMR). Yield of known dimer **6'** quantified by integration of the olefinic signal at 5.52 ppm (1H; Figure S17).[7] Yields and TONs are given in Table 1 in the main text. Isomerization was assessed at high catalyst loading (1 mol %) to maximize its probability, as C=C migration is known to increase at higher proportions of Ru.[7,11]

ESI-MS (MeOH). *m/z* = 519 (M+Na; **6'**), 505 (M+Na–14; **6"**; 7% vs Σ **6'**+**6"**). The proportion of **6"** is calculated based on the assumption of equal lifetimes for **6'** and **6"** (internal olefins differing by one methylene unit).

S1.5. Monitoring Catalyst Stability by UV-Vis Spectroscopy.

S1.5.1. Stability of HC1s^{Me} in water. To a quartz cuvette was added H_2O (1.97 mL, pH = 7 prior to catalyst addition), and a 30 µL aliquot of a stock solution of **HC1s^{Me}** in water (4.2 mg/mL), to give a final Ru concentration of 30 µM. The cuvette was sealed, wrapped with Parafilm and removed from the glovebox to the spectrometer. The first UV-vis spectrum was taken 5 min after preparing the catalyst stock solution. Subsequent spectra were recorded periodically up to 24 h. UV-vis spectra showing the stability of **HC1s^{Me}** vs **AM** appear in Figure 2 in the main text.

With NaCl: Solid NaCl (234 mg, 4.00 mmol) was added to the cuvette prior to catalyst. UV-vis spectra for **HC1s^{Me}** and **AM** appear in Figure S18.

S2. NMR Spectra.

(a) ^{1}H NMR spectrum of **C1$_S$^{Me}**•HBF_4

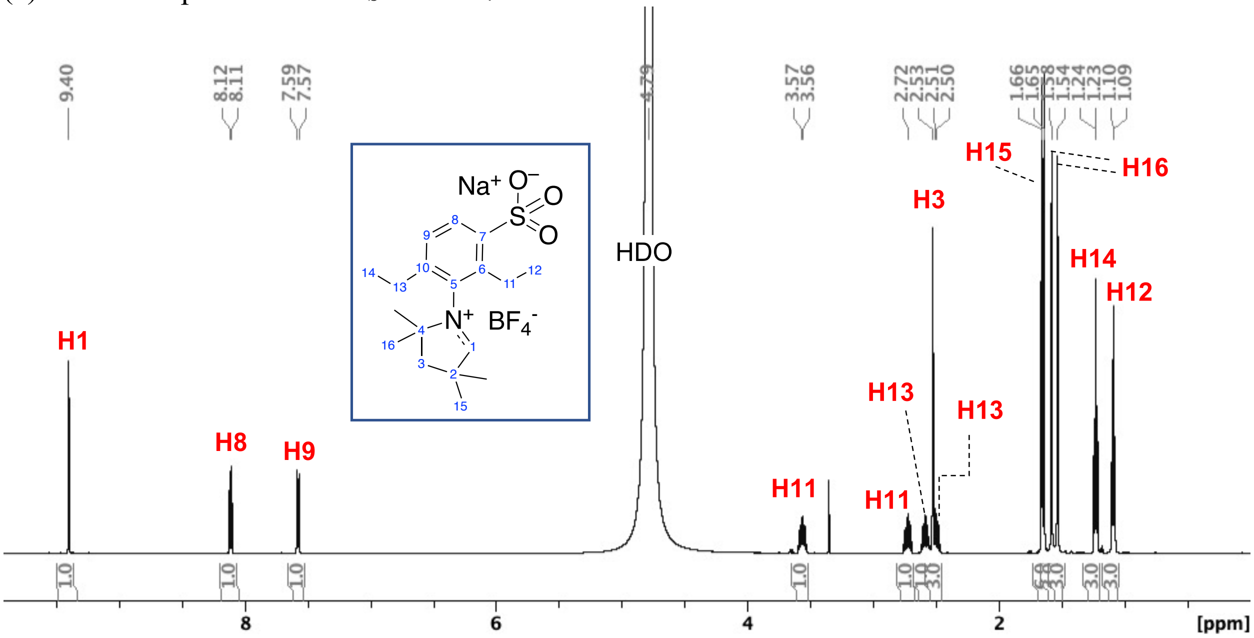

(b) $^{13}C\{^1H\}$ NMR spectrum of **C1$_S$^{Me}**•HBF_4

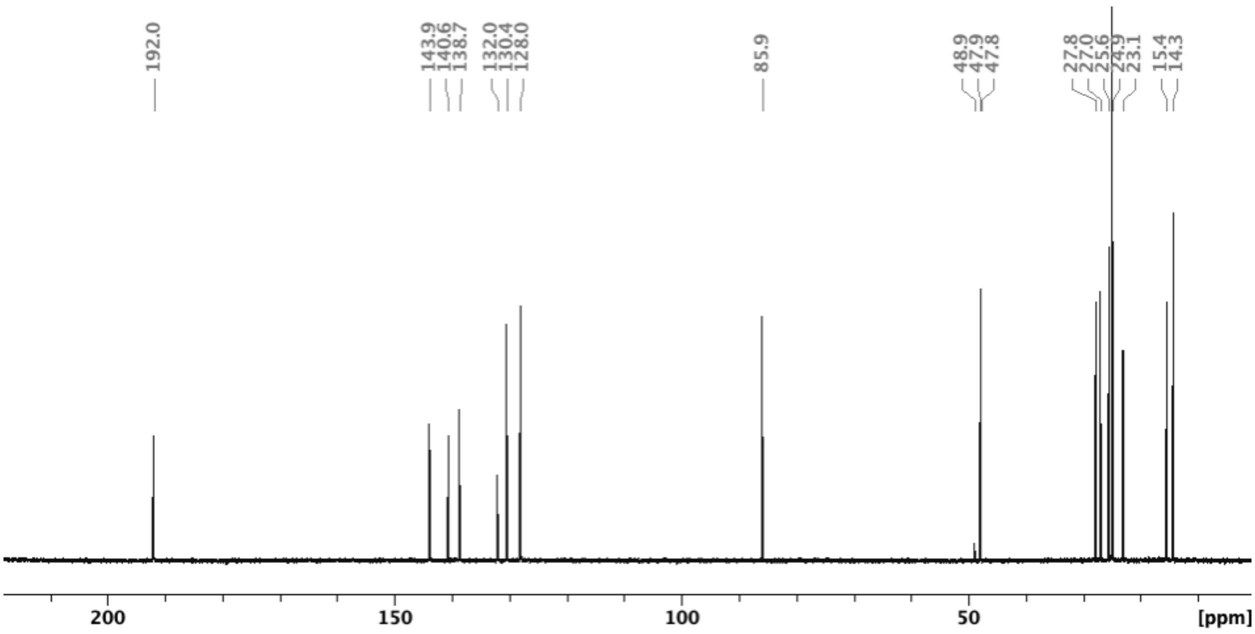

Figure continues next page

(c) ^{1}H-^{1}H COSY NMR spectrum of **C1s^{Me}**•HBF_4

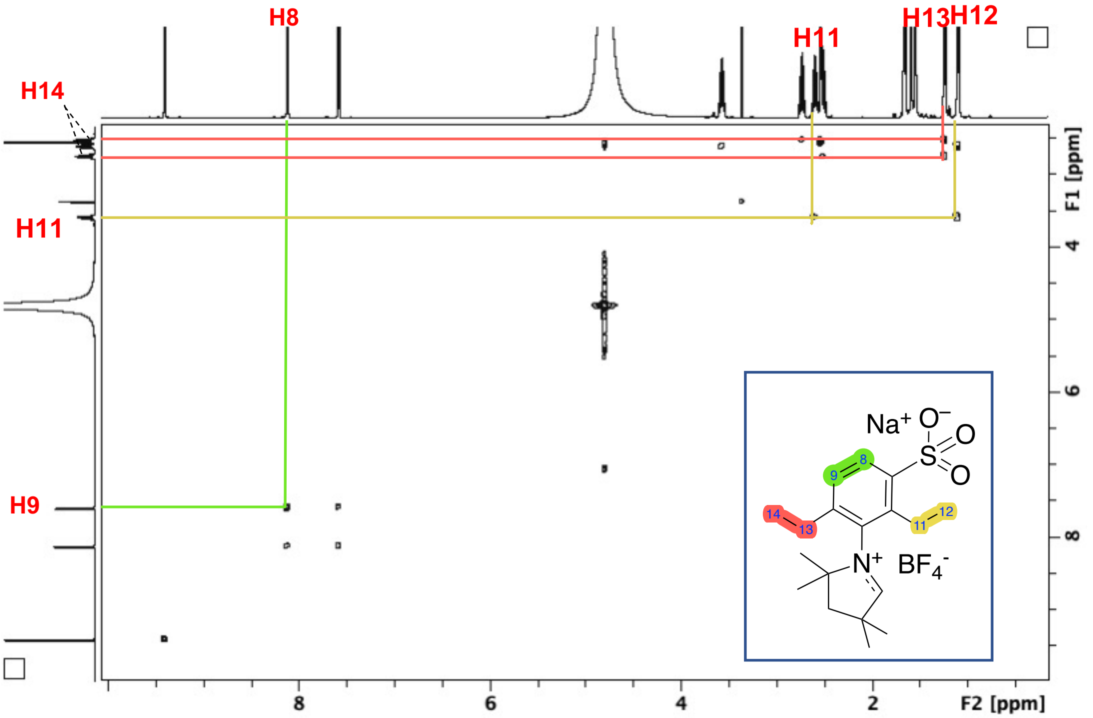

(d) ^{1}H-^{13}C HSQC NMR spectrum of **C1s^{Me}**•HBF_4

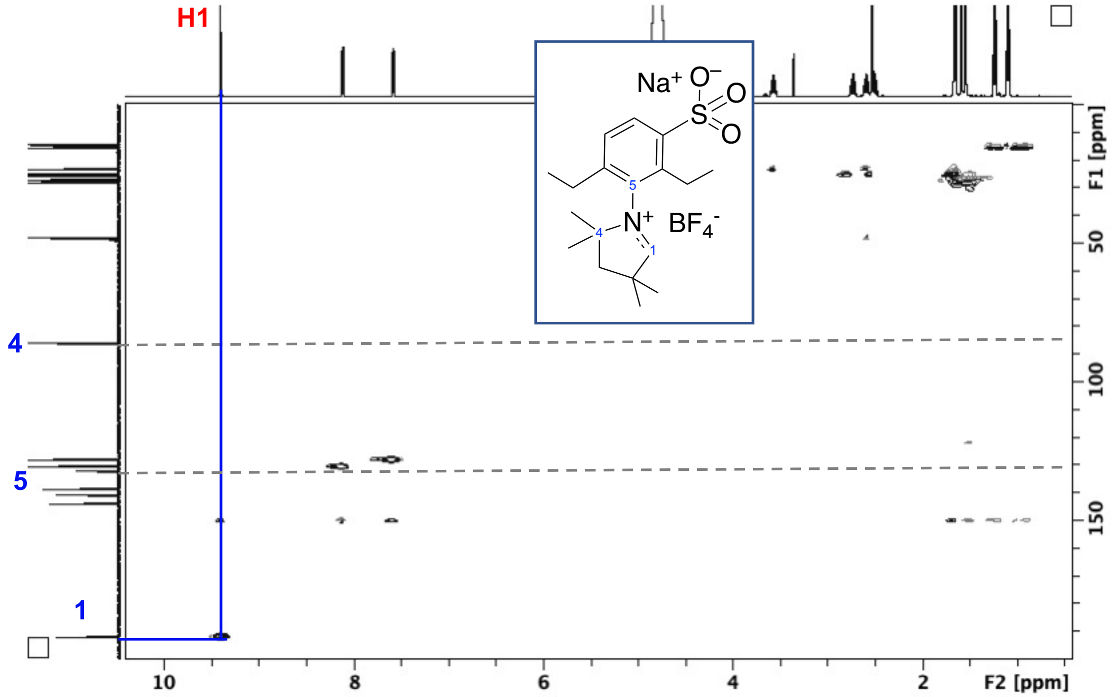

Figure continues next page

(e) ^{1}H-^{13}C HMBC NMR spectrum of **C1**$_{\mathbf{S}}^{\mathbf{Me}}$•$HBF_4$

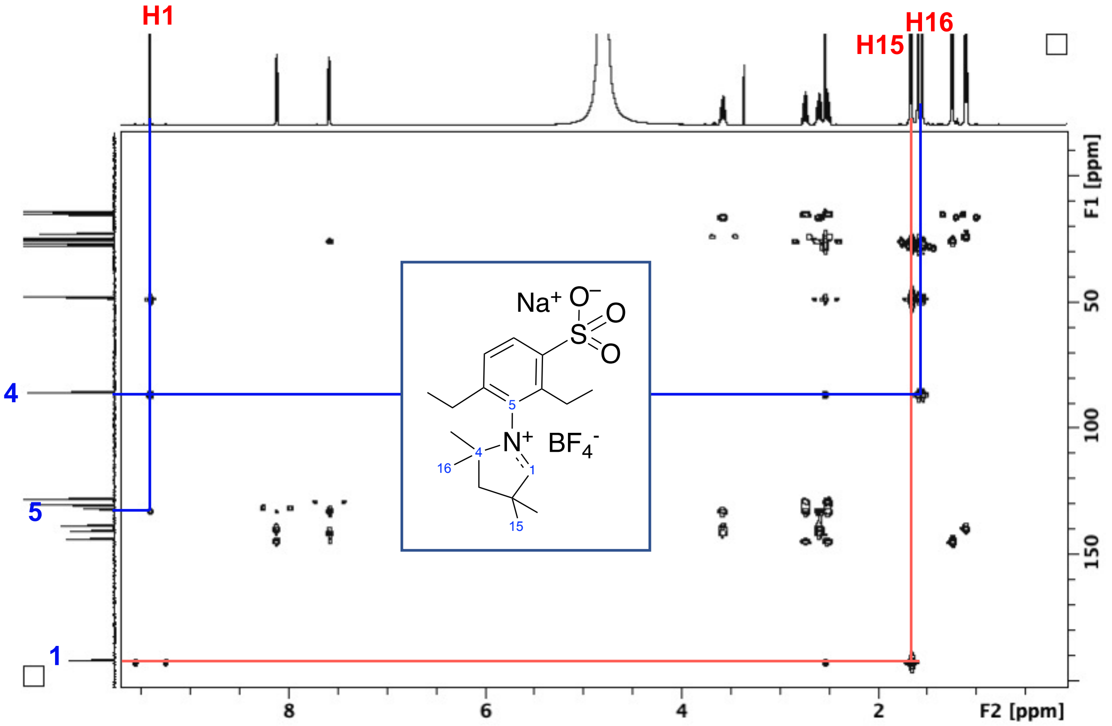

Figure S1. NMR characterization of CAAC salt **C1**$_{\mathbf{S}}^{\mathbf{Me}}$•$HBF_4$ in D_2O.(a) ^{1}H NMR (600 MHz). (b) $^{13}C\{^{1}H\}$ NMR (150 MHz). (c) ^{1}H-^{1}H COSY NMR (600 MHz). (d) ^{1}H-^{13}C HSQC NMR (600/150 MHz). Grey dashed lines indicate key 4° carbon signals (note absence of $^{1}J_{HC}$ correlations). (e) ^{1}H-^{13}C HMBC NMR (600/150 MHz).

(a) ^{1}H NMR spectrum of **C1$_{S}^{Cy}$**•HBF_4

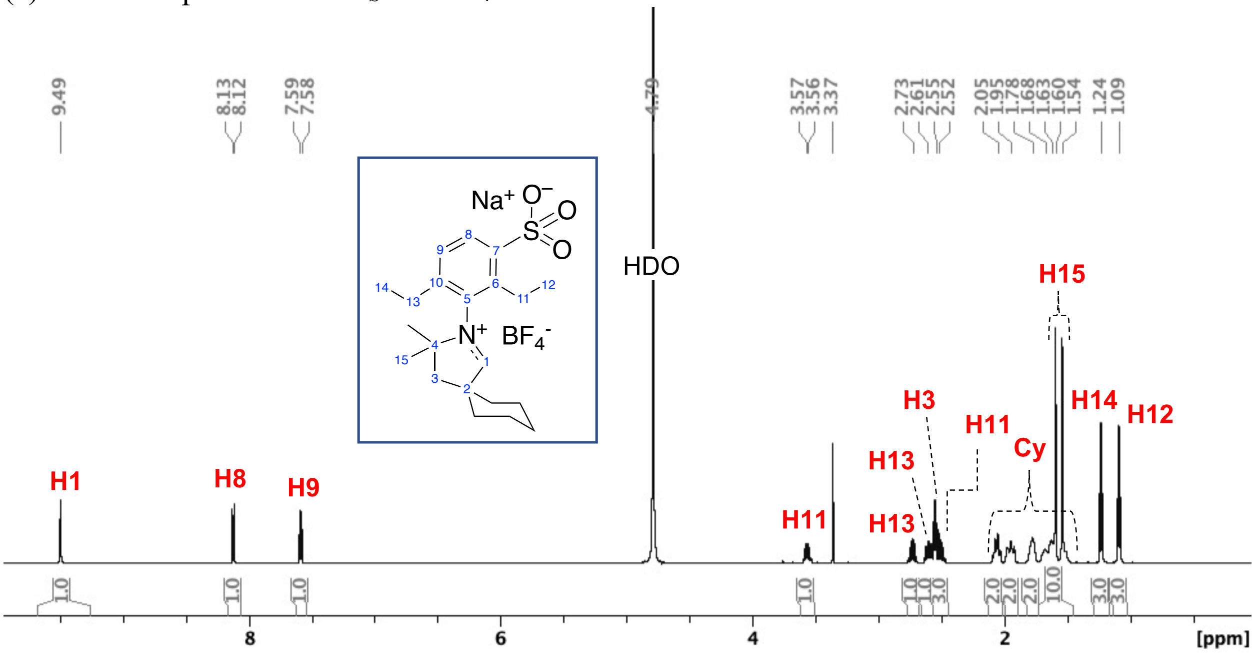

(b) ^{13}C{^{1}H} NMR spectrum of **C1$_{S}^{Cy}$**•HBF_4

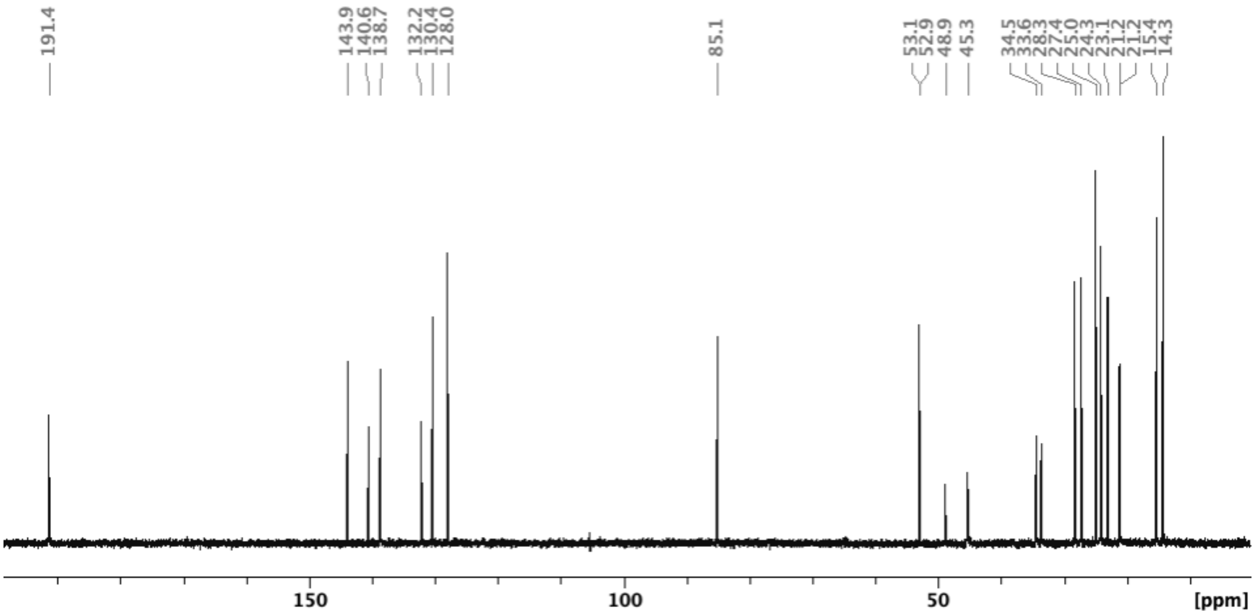

Figure continues next page

(c) ^{1}H-^{1}H COSY NMR spectrum of **C1s**^{Cy}•HBF_4

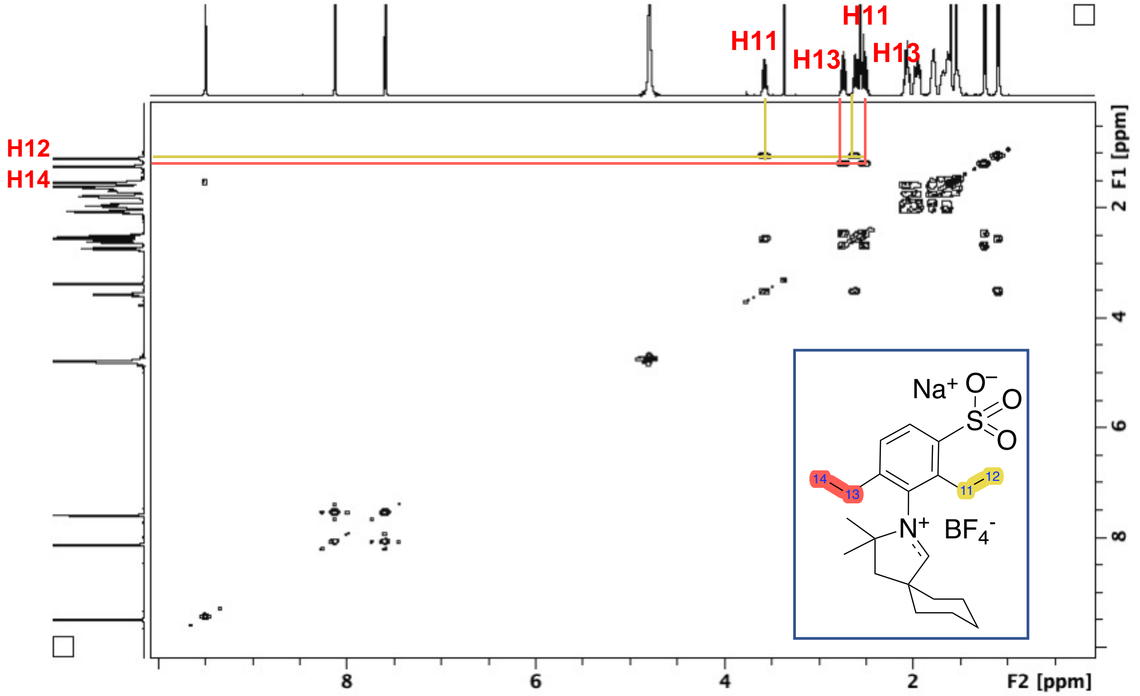

(d) ^{1}H-^{13}C HSQC NMR spectrum of **C1s**^{Cy}•HBF_4

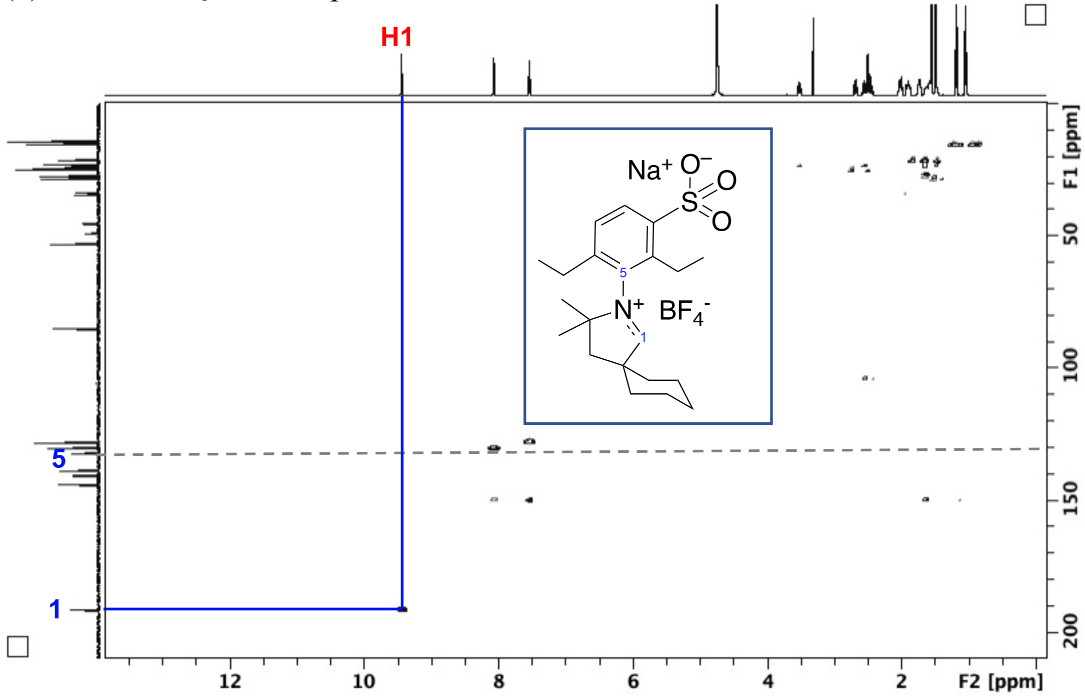

Figure continues next page

(e) ^{1}H-^{13}C HMBC NMR spectrum of **C1s^{Cy}**•HBF$_4$

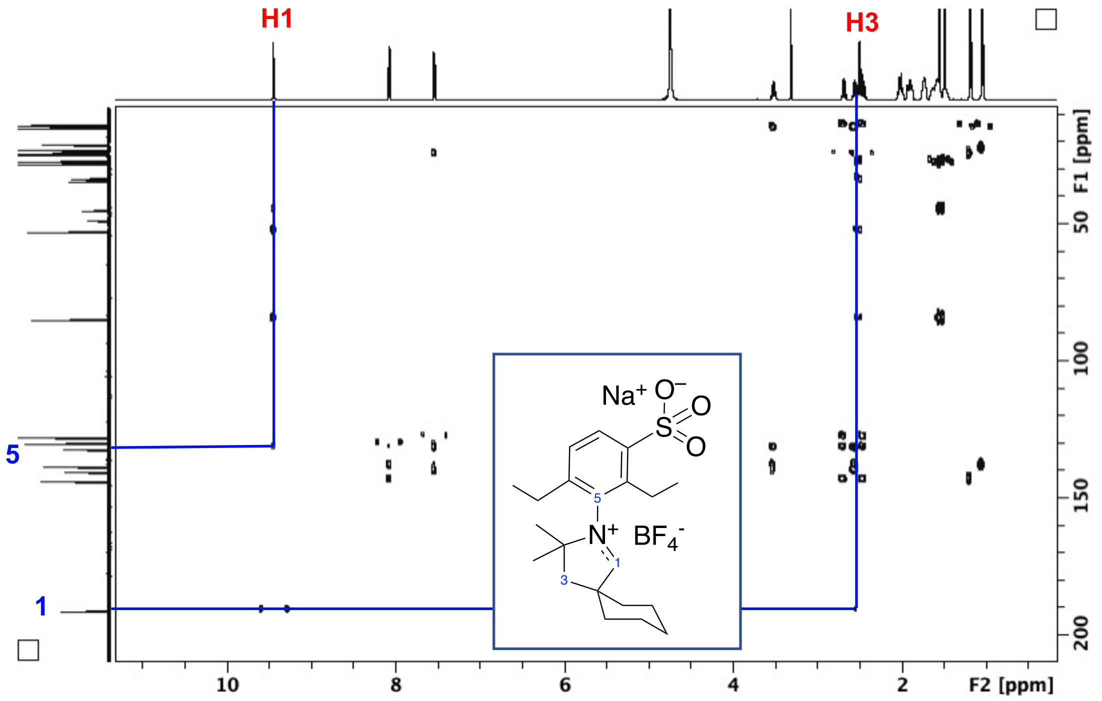

Figure S2. NMR characterization of CAAC salt **C1s^{Cy}**•HBF$_4$ in D$_2$O. (a) ^{1}H NMR (600 MHz). (b) ^{13}C{^{1}H} NMR (150 MHz). (c) ^{1}H-^{1}H COSY NMR (600 MHz). (d) ^{1}H-^{13}C HSQC NMR (600/150 MHz). Grey dashed lines indicate key 4° carbon signals (note absence of $^1J_{HC}$ correlations). (e) ^{1}H-^{13}C HMBC NMR (600/150 MHz).

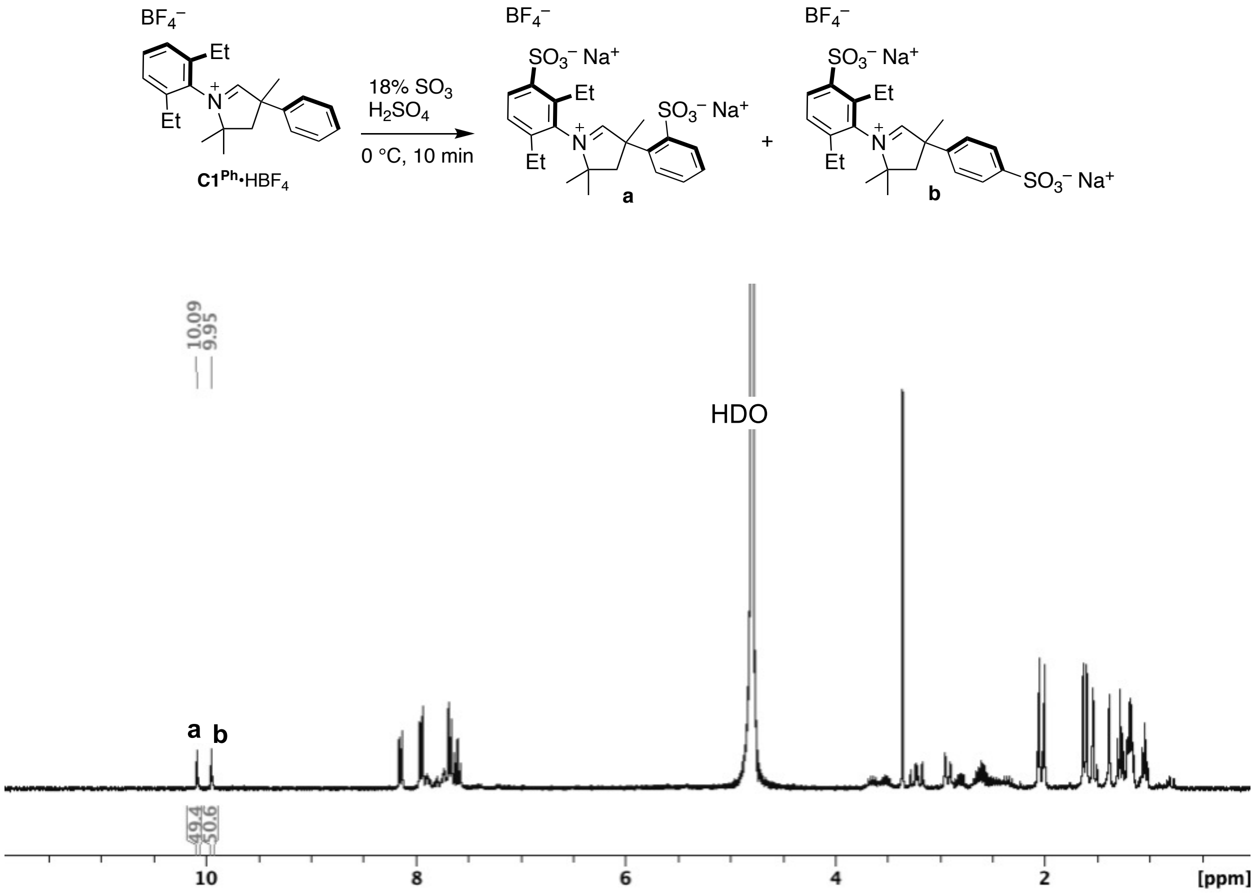

Figure S3. ^{1}H NMR spectrum (300 MHz, D_2O) of **C1s^{Ph}**•HBF_4 after sulfonation, showing mixture of products.

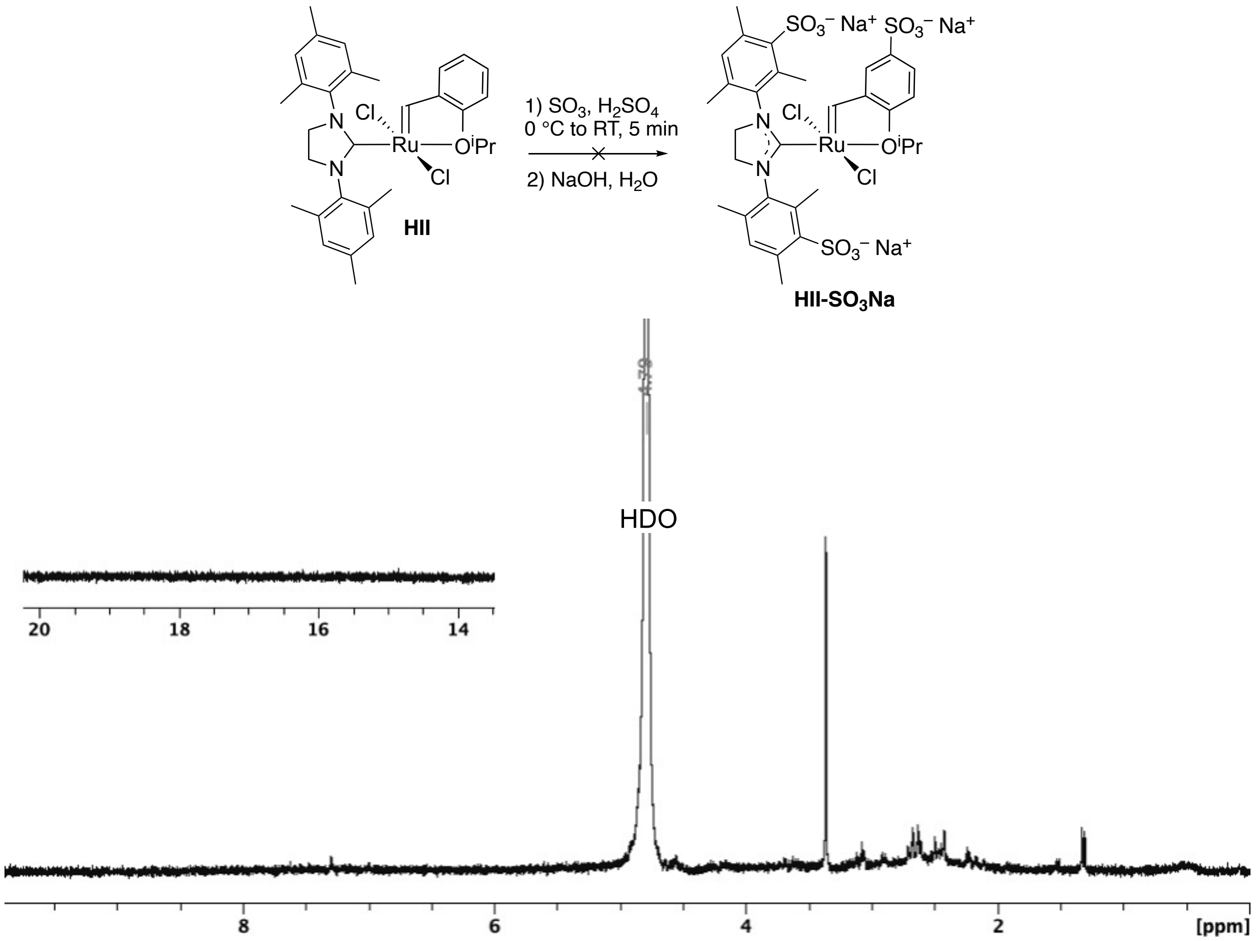

Figure S4. ^{1}H NMR spectrum (300 MHz, D_2O) after attempted sulfonation of **HII**. The inset shows the loss of signals in the alkylidene region.

(a) ^{1}H NMR spectrum of **HC1$_{S}$^{Me} -I$_2$**

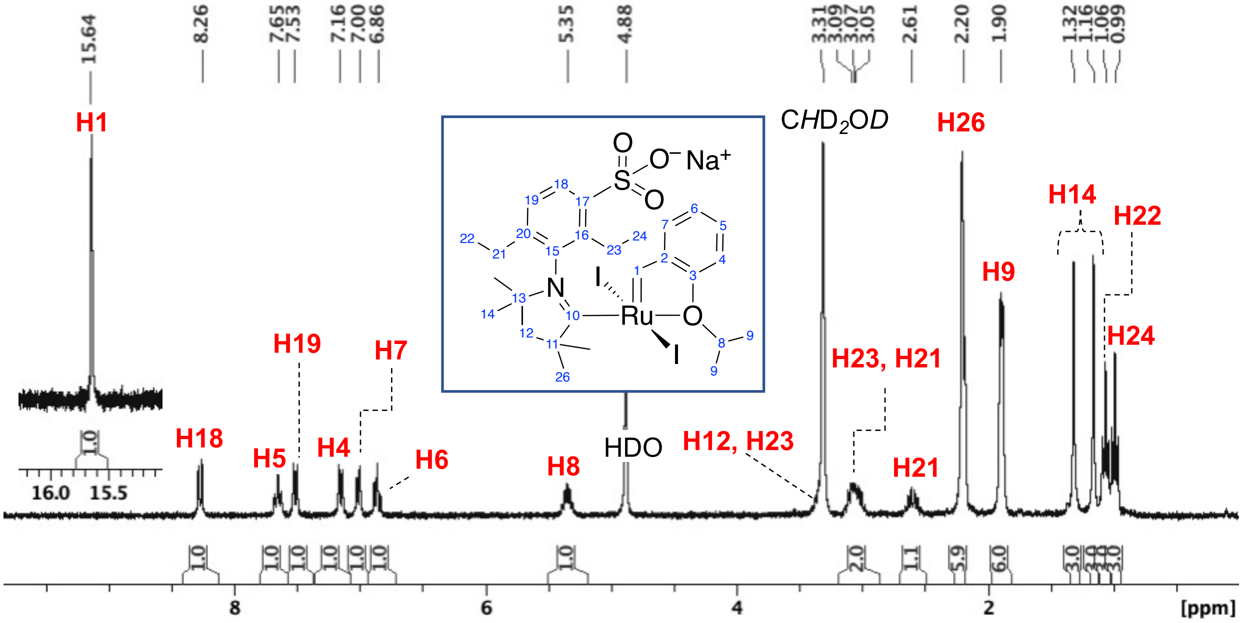

(b) ^{13}C{^{1}H} NMR spectrum of **HC1$_{S}$^{Me} -I$_2$**

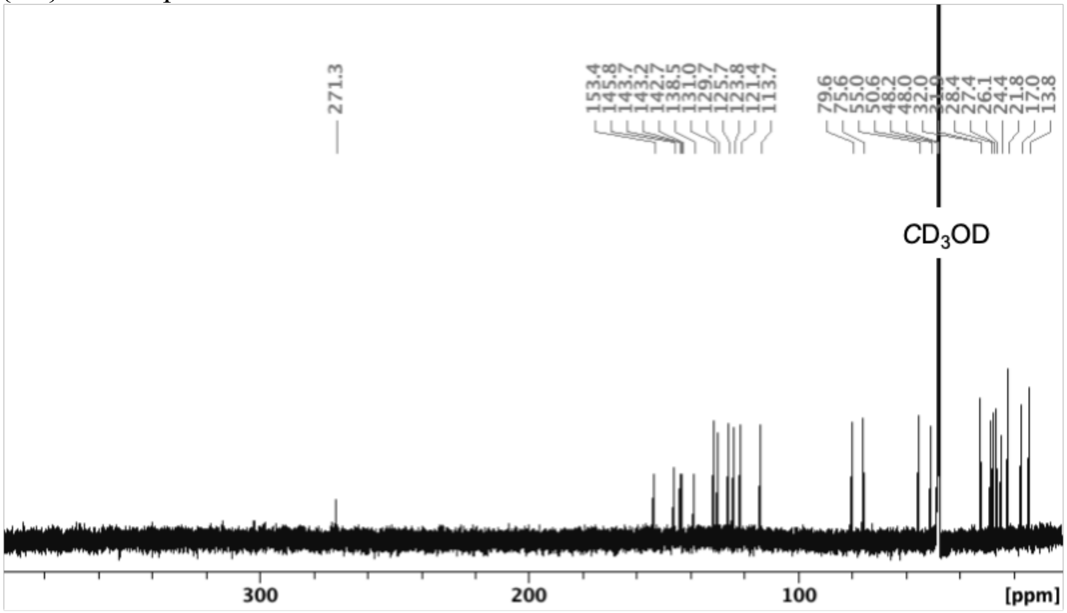

Figure continues next page

(c) ^{1}H-^{1}H COSY NMR spectrum of **HC1$_S^{Me}$ -I$_2$**

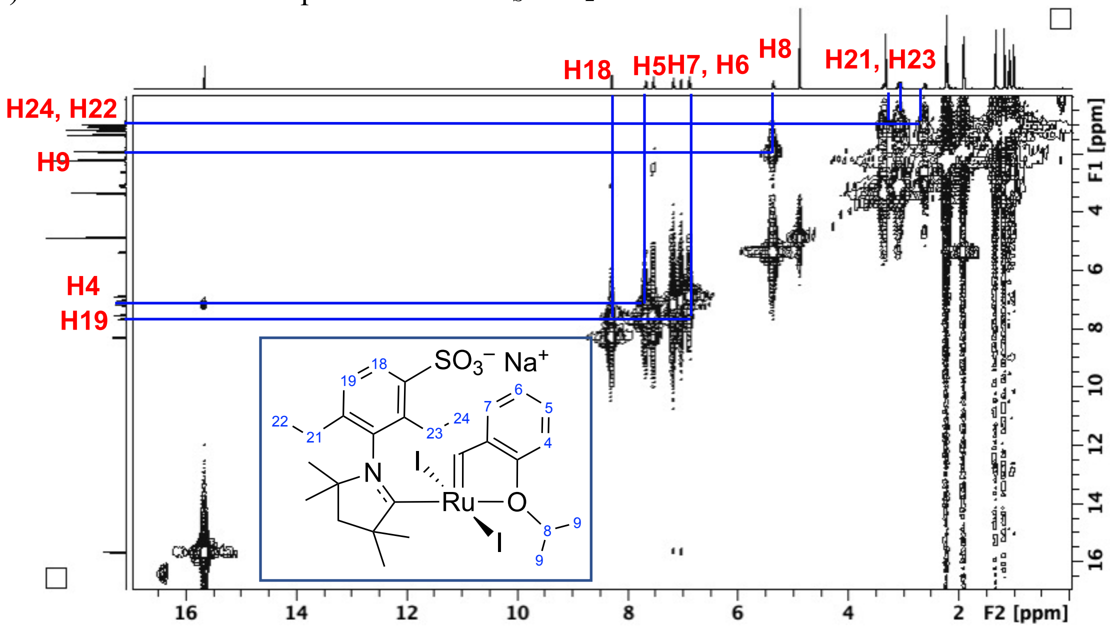

(d) ^{1}H-^{13}C HSQC NMR spectrum of **HC1$_S^{Me}$-I$_2$**

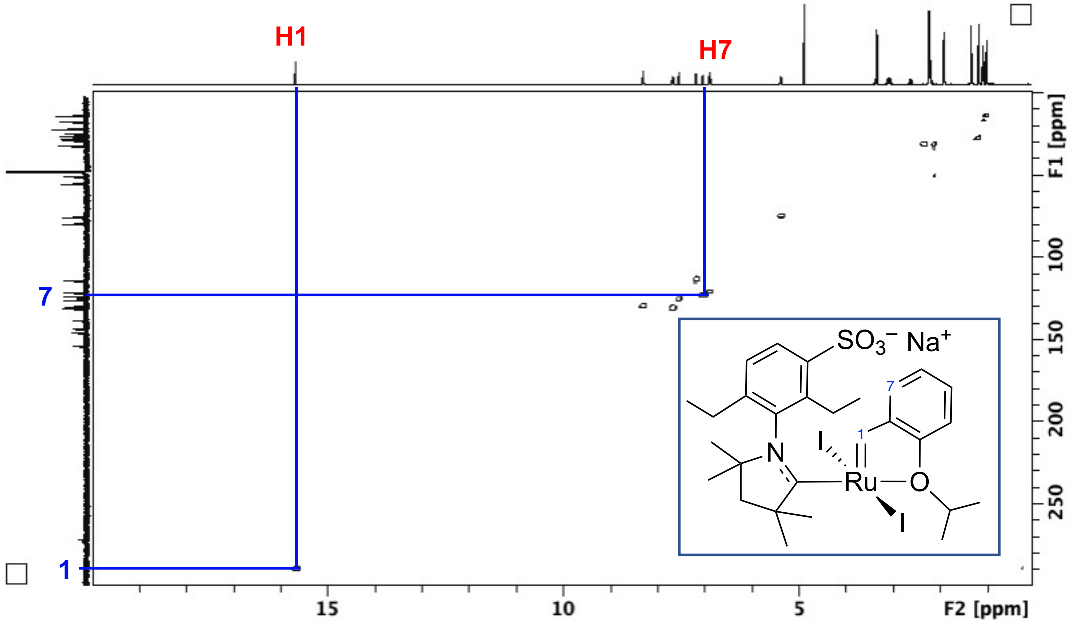

Figure continues next page

(e) ^{1}H-^{13}C HMBC NMR spectrum of **HC1$_S$^{Me}-I$_2$**

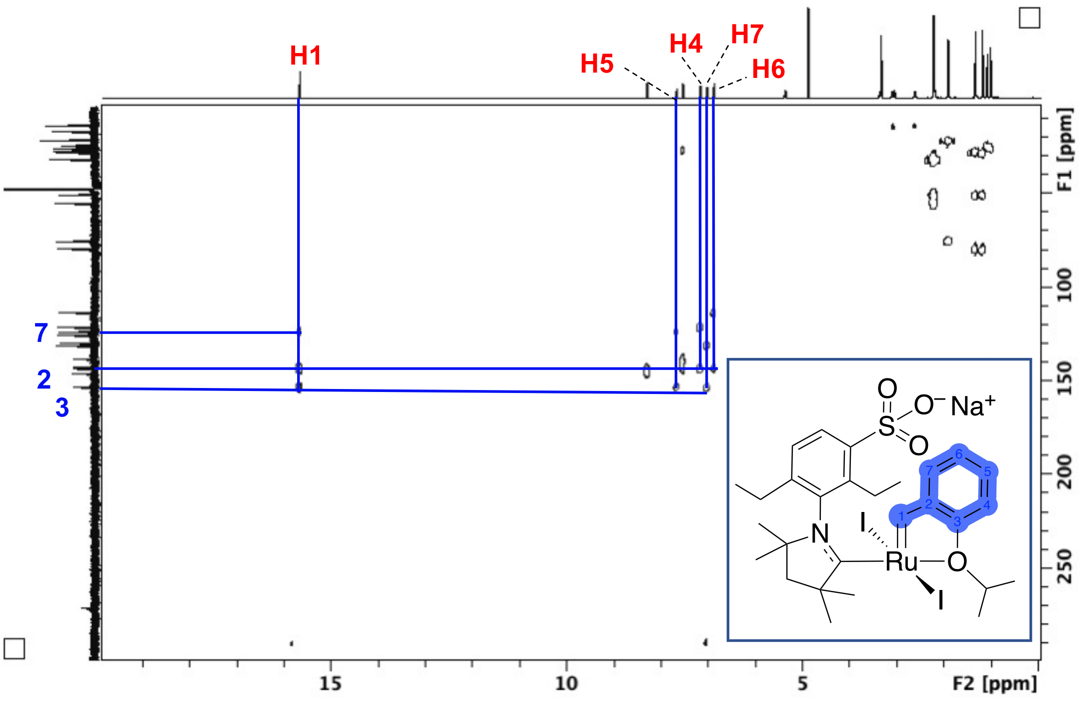

Figure S5. NMR characterization of synthetic intermediate **HC1$_S$^{Me}-I$_2$** in CD_3OD. (a) ^{1}H NMR (300 MHz; inset shows alkylidene signal. (b) ^{13}C{^{1}H} NMR (150 MHz; alkylidene C not observed; located by ^{1}H-^{13}C HSQC). (c) ^{1}H-^{1}H COSY NMR (600 MHz). (d) ^{1}H-^{13}C HSQC NMR spectrum (600/150 MHz). (e) ^{1}H-^{13}C HMBC NMR (600/150 MHz).

(a) ^{1}H NMR spectrum of **HC1$_S$^{Cy}-I$_2$**

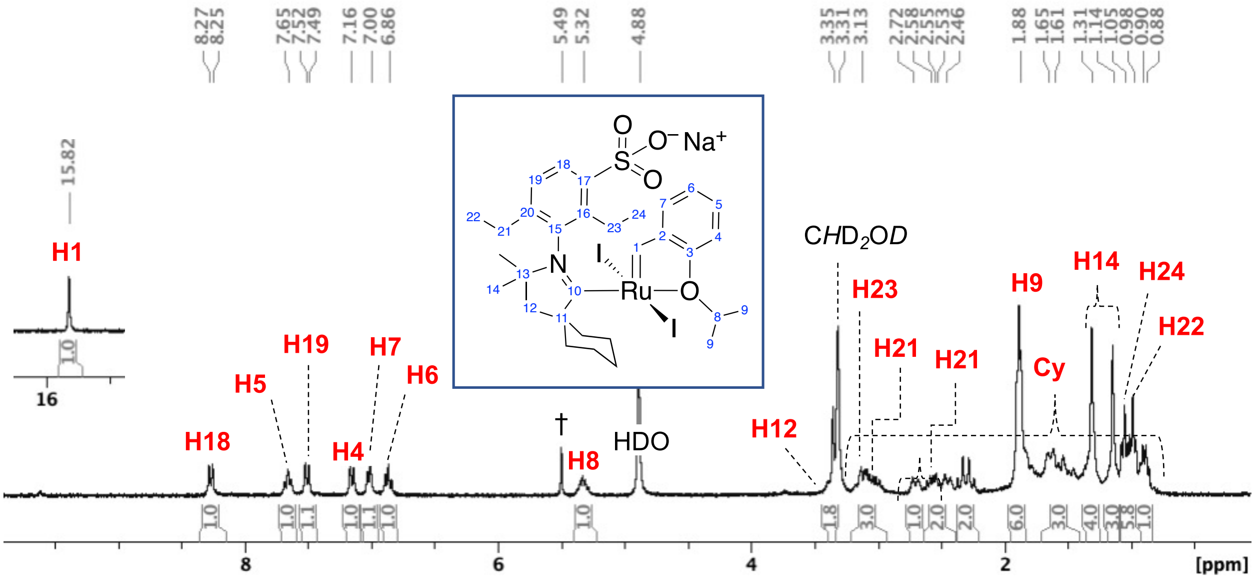

(b) ^{13}C{^{1}H} NMR spectrum of **HC1$_S$^{Cy}-I$_2$**

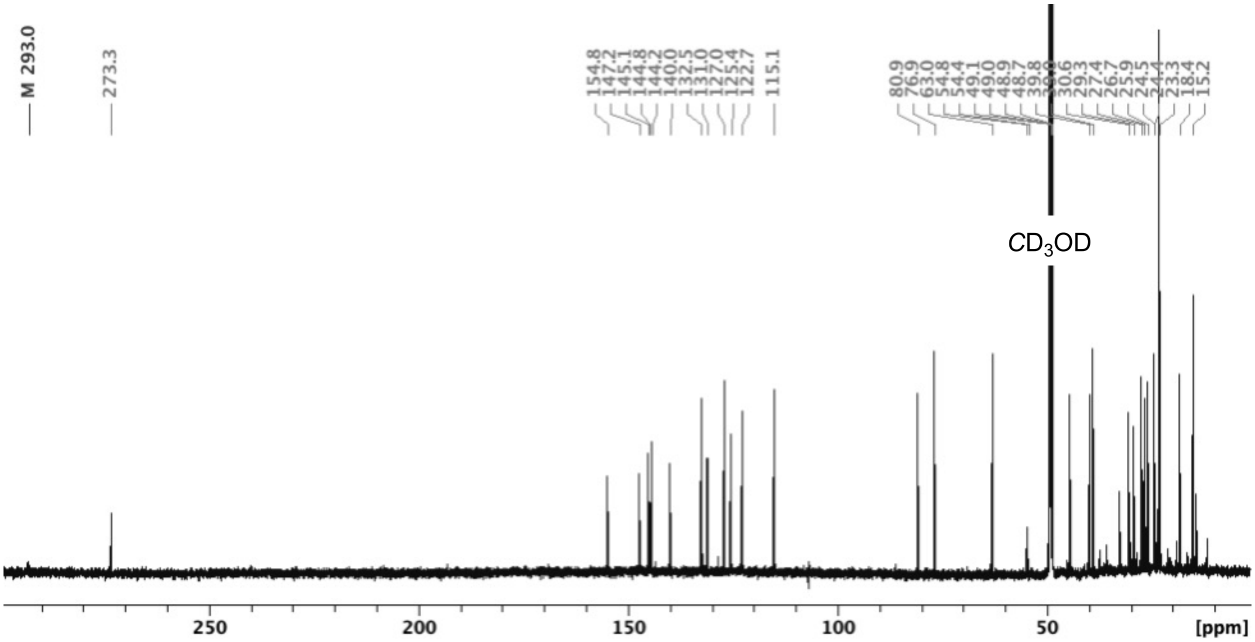

Figure continues next page

(c) ^{1}H-^{1}H COSY NMR spectrum of $\mathbf{HC1_S^{Cy}}$-$\mathbf{I_2}$

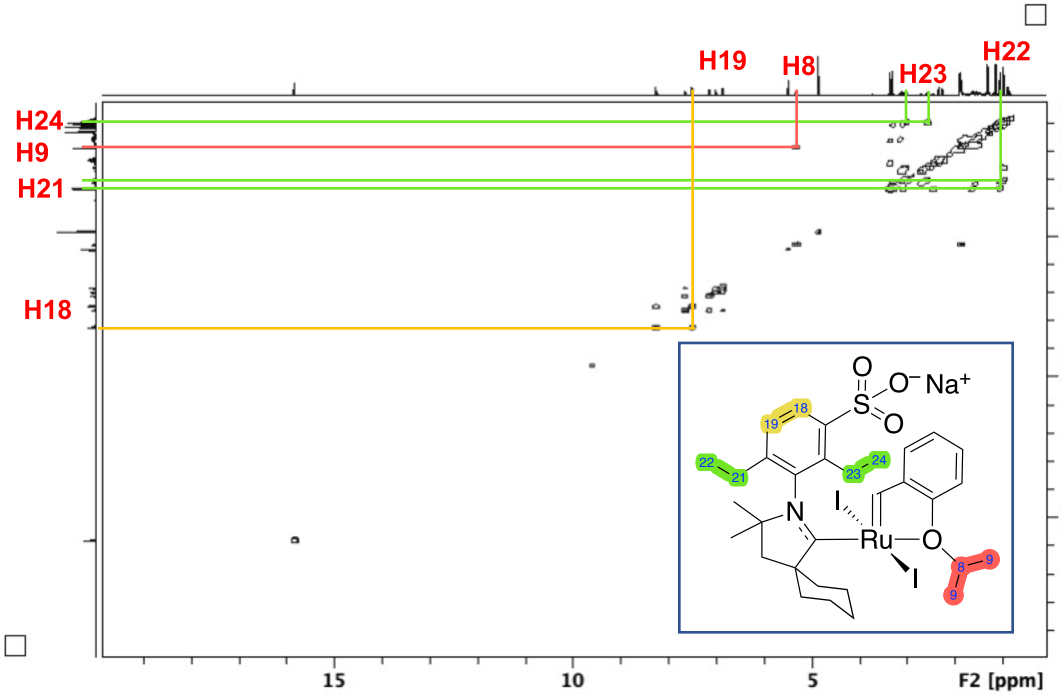

(d) ^{1}H-^{13}C HSQC NMR spectrum of $\mathbf{HC1_S^{Cy}}$-$\mathbf{I_2}$

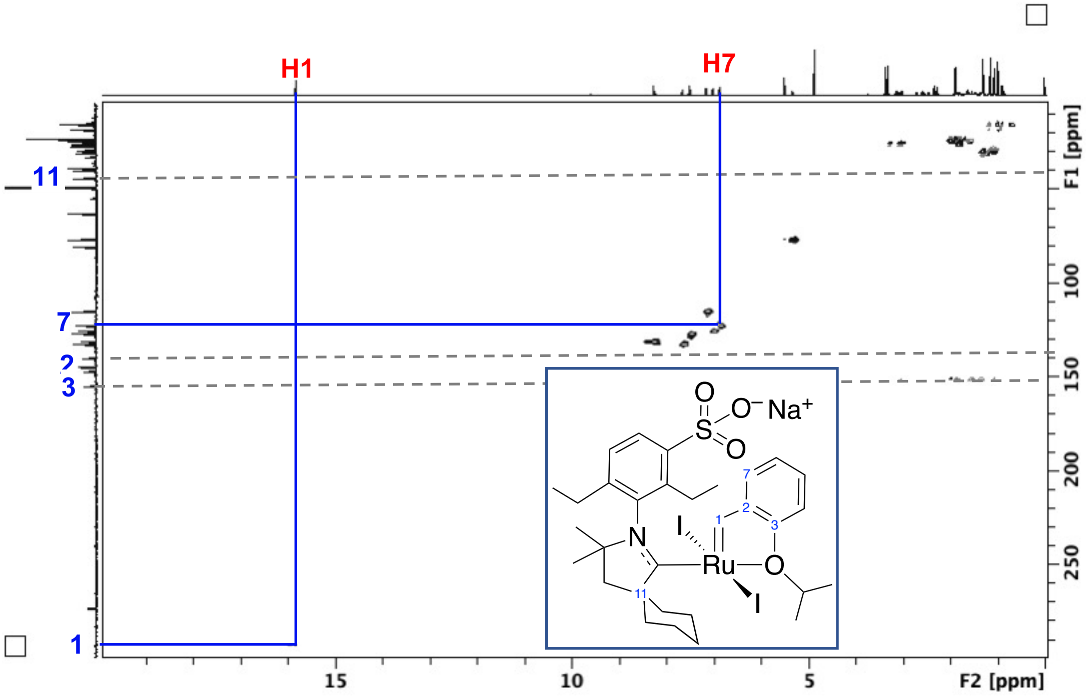

Figure continues next page

(e) ^{1}H-^{13}C HMBC NMR spectrum of **HC1$_{S}^{Cy}$-I$_2$**

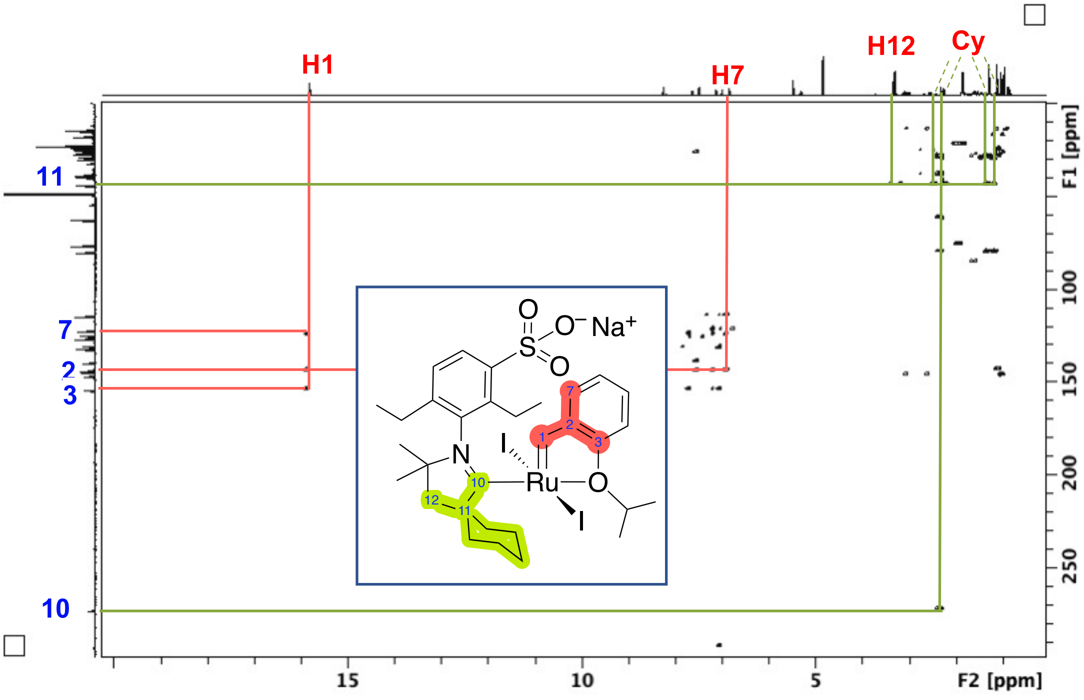

Figure S6. NMR characterization of **HC1$_{S}^{Cy}$-I$_2$** in CD_3OD. (a) ^{1}H NMR (300 MHz; inset shows alkylidene signal, (†) indicates residual CH_2Cl_2). (b) ^{13}C{^{1}H} NMR (150 MHz; alkylidene C not observed; located by ^{1}H-^{13}C HSQC). (c) ^{1}H-^{1}H COSY NMR (600 MHz). (d) ^{1}H-^{13}C HSQC NMR (600/150 MHz). Grey dashed lines indicate key 4° carbon signals (note absence of $^{1}J_{HC}$ correlations). (e) ^{1}H-^{13}C HMBC NMR (600/150 MHz).

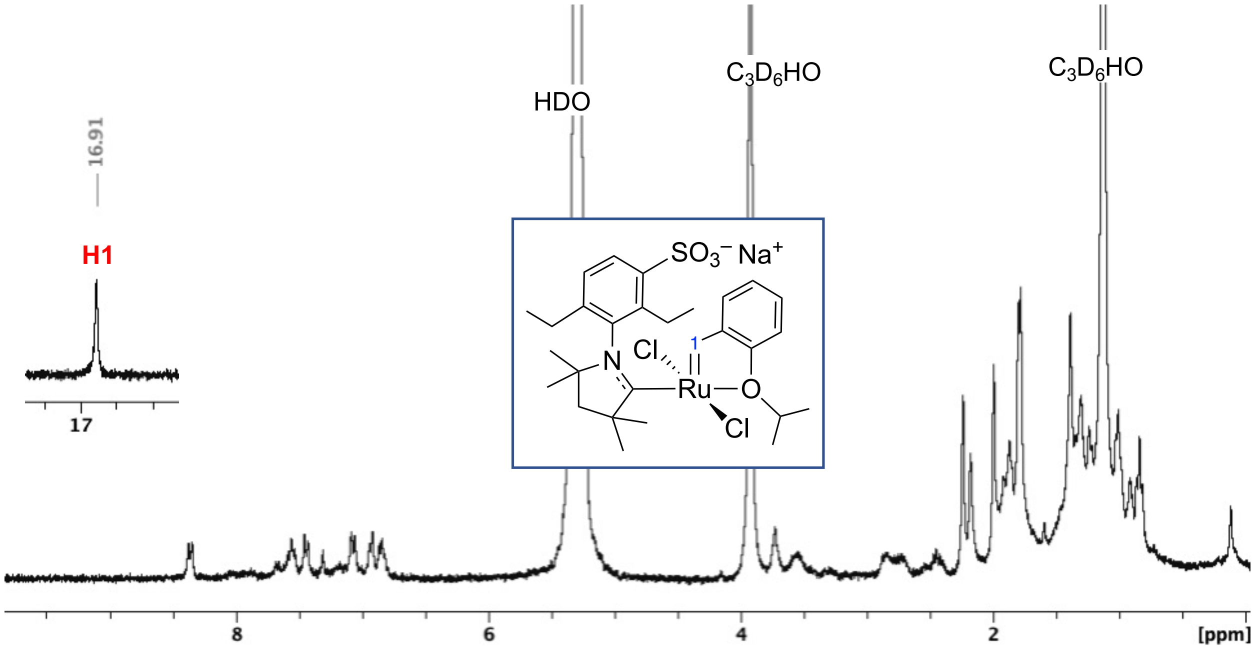

Figure S7. ^{1}H NMR spectrum (300 MHz, isopropanol-d$_7$) of isolated crude **HC1s^{Me}**, prepared from **HI**.

(a) ^{1}H NMR spectrum showing the benzylidene abstraction product **1a**

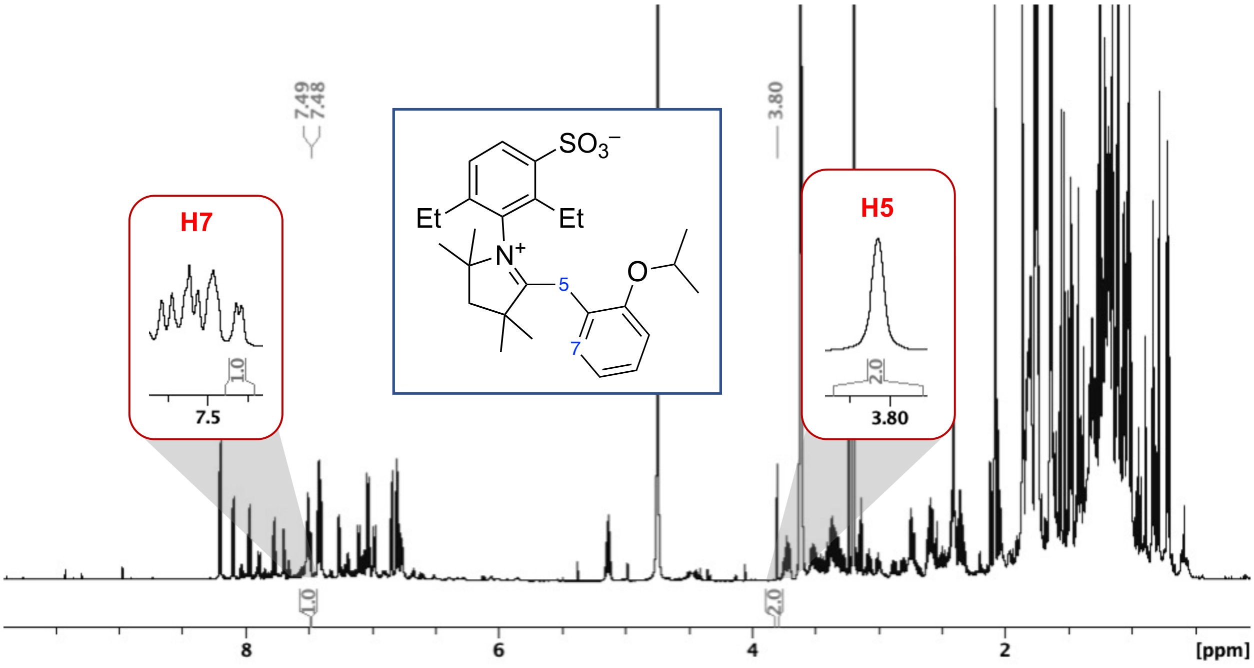

(c) ^{1}H-^{1}H COSY NMR spectrum showing the benzylidene abstraction product **1a**

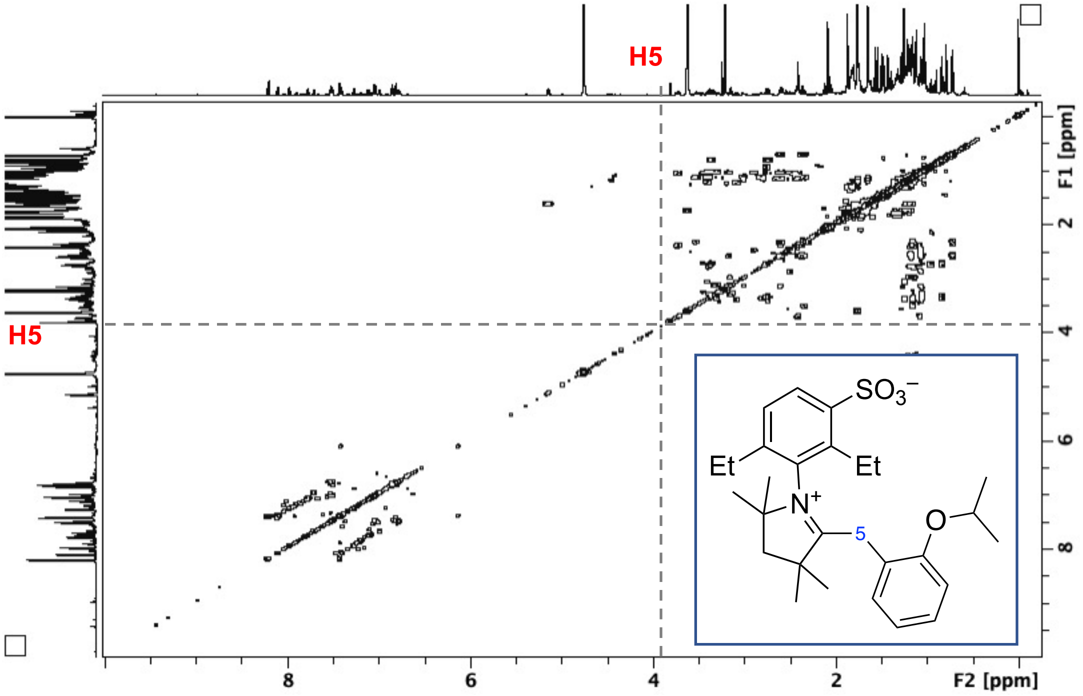

(e) ^{1}H-^{13}C HSQC NMR spectrum showing the benzylidene abstraction product **1a**

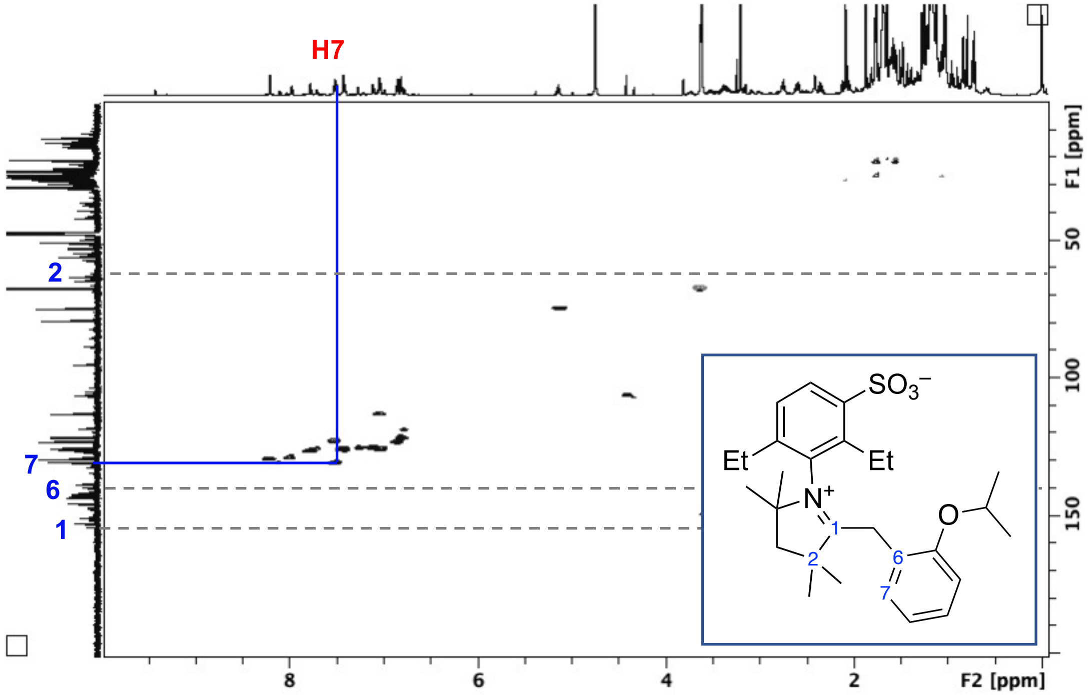

(d) ^{1}H-^{13}C HMBC NMR spectrum showing the benzylidene abstraction product **1a**

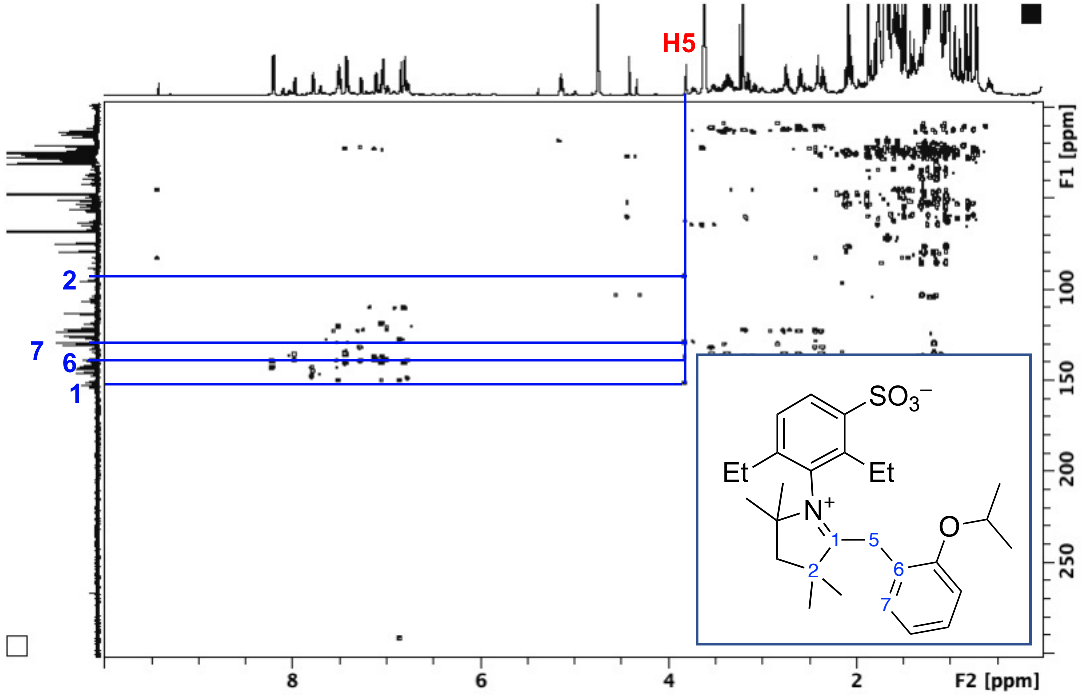

Figure S8. NMR spectra in CD_3OD, showing benzylidene abstraction product **1a** formed in the reaction of **HC1$_S^{Me}$** with **HI**. (a) ^{1}H NMR (600 MHz). (b) ^{1}H-^{1}H COSY NMR (600 MHz). (c) ^{1}H-^{13}C HSQC NMR (600/150 MHz). Grey dashed lines indicate key 4° carbon signals (note absence of $^{1}J_{HC}$ correlations). (d) ^{1}H-^{13}C HMBC NMR (600/150 MHz).

(a) ^{19}F NMR showing the borylation product **1b**.

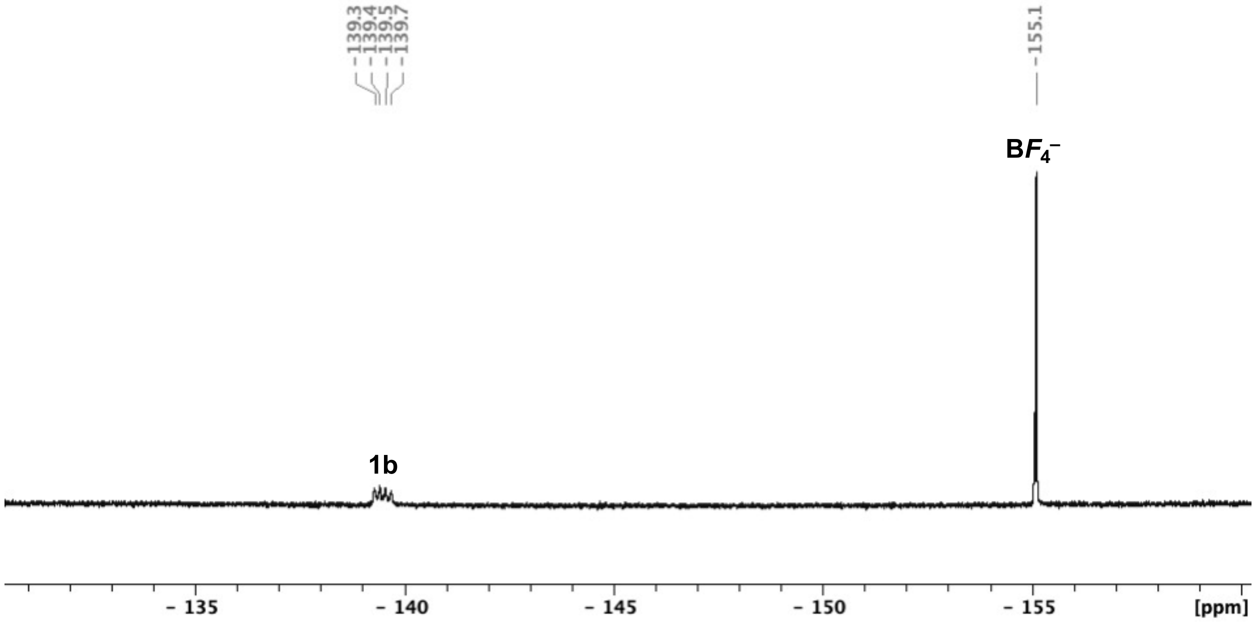

(b) ^{11}B{^{1}H} NMR showing the borylation product **1b**.

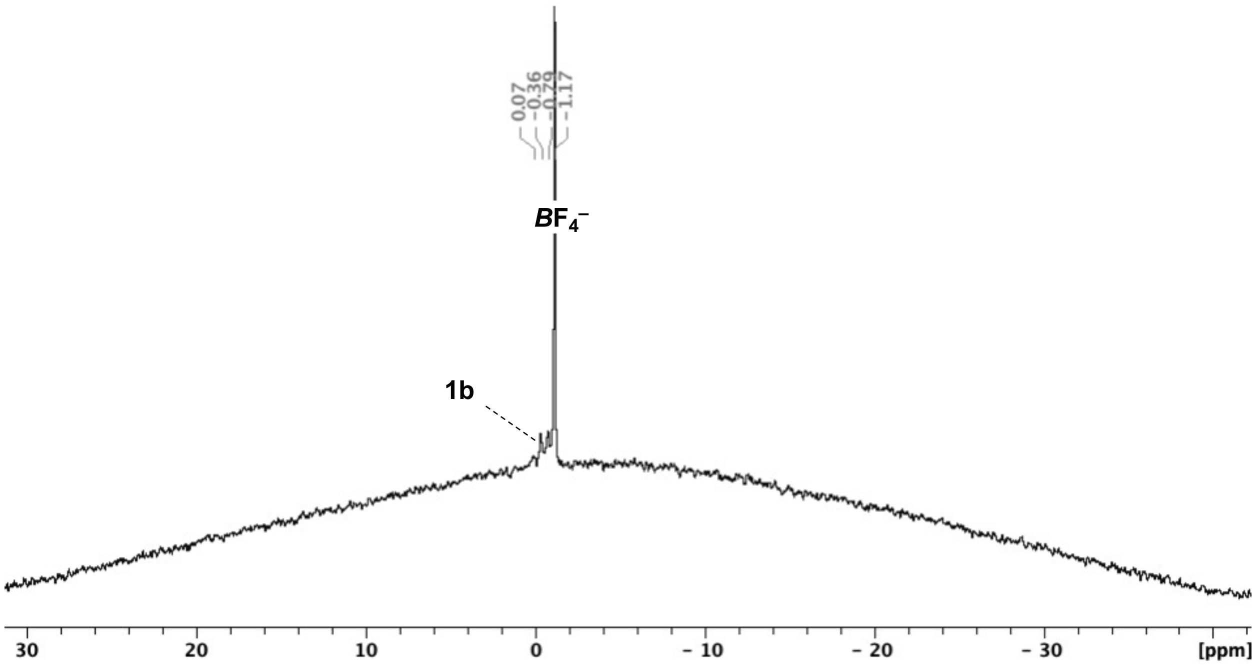

Figure S9. NMR spectra for the crude reaction mixture from the synthesis of $\mathbf{HC1_S^{Me}}$ via **HI**, showing signals for borane adduct $\mathbf{C1_S^{Me}}$-BF_3 (**1b**). (a) ^{19}F NMR (282 MHz, CD_3OD). (b) ^{11}B{^{1}H} NMR (96 MHz, CD_3OD). Note overlap with BF_4^- singlet in (b).

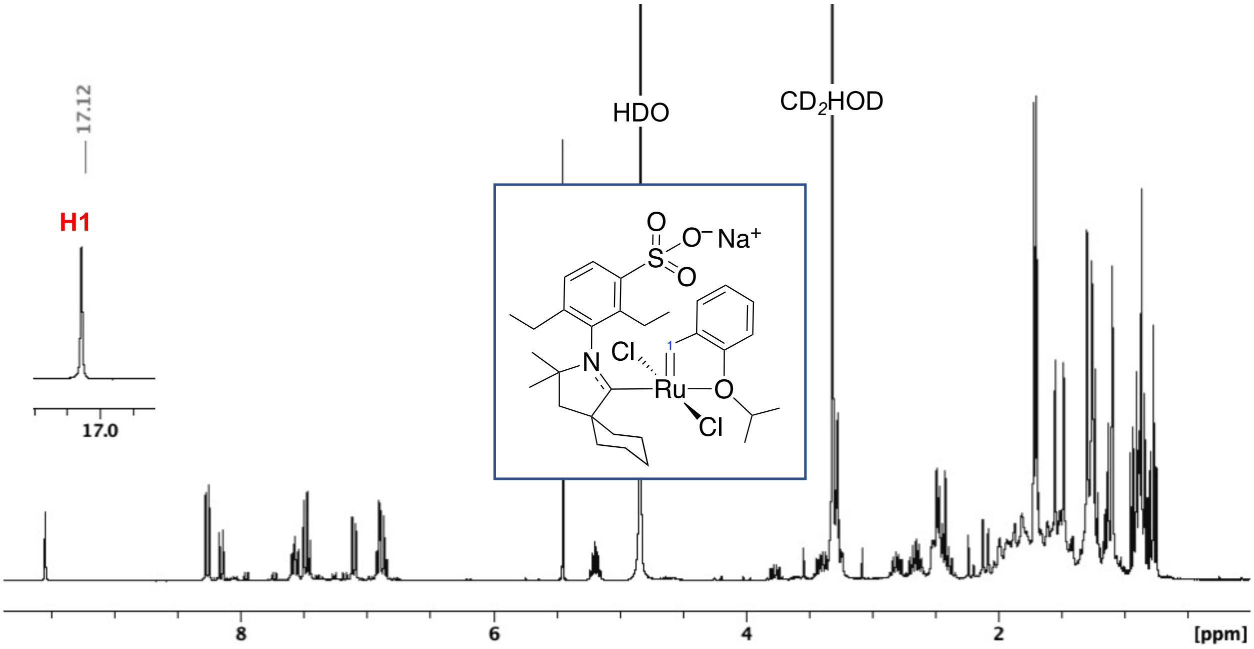

Figure S10. ^{1}H NMR spectrum (300 MHz, CD_3OD) of isolated impure **HC1$_{S}$^{Cy}** prepared by ligand exchange with **HI**. For comparison, see spectra for material prepared via ligand exchange with **HI-I$_2$** in Figure S13.

(a) ^{1}H NMR spectrum of **HC1s^{Me}**

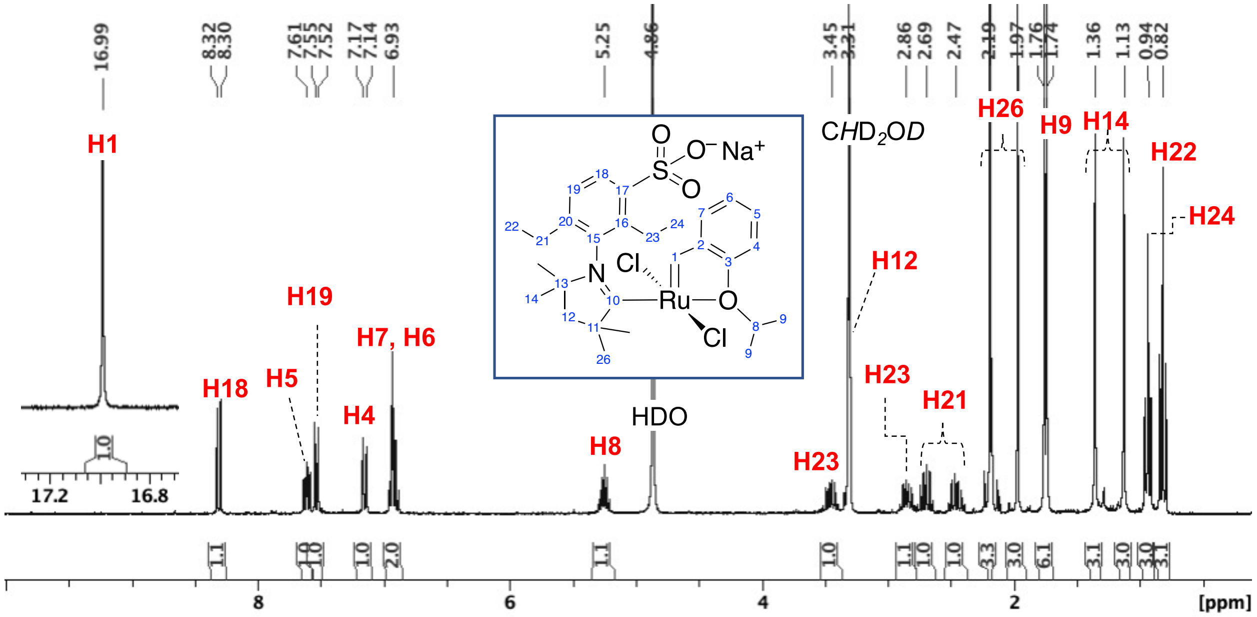

(b) ^{13}C{^{1}H} NMR spectrum of **HC1s^{Me}**

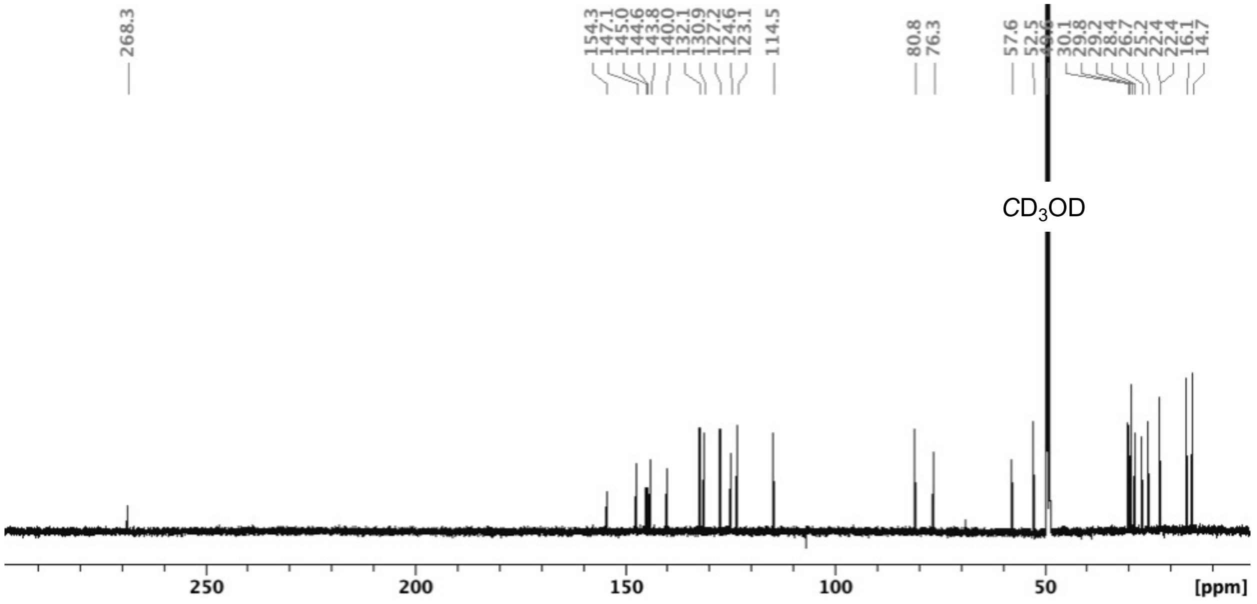

Figure continues next page

(c) ^{1}H-^{1}H COSY NMR spectrum of **HC1s^{Me}**

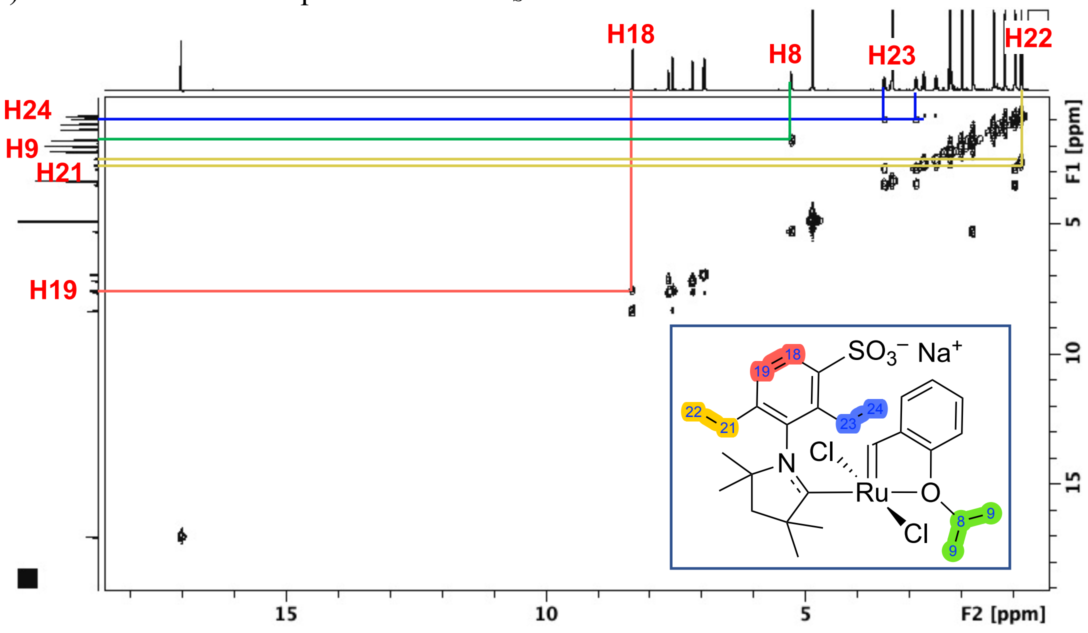

(d) ^{1}H-^{13}C HSQC NMR spectrum of **HC1s^{Me}**

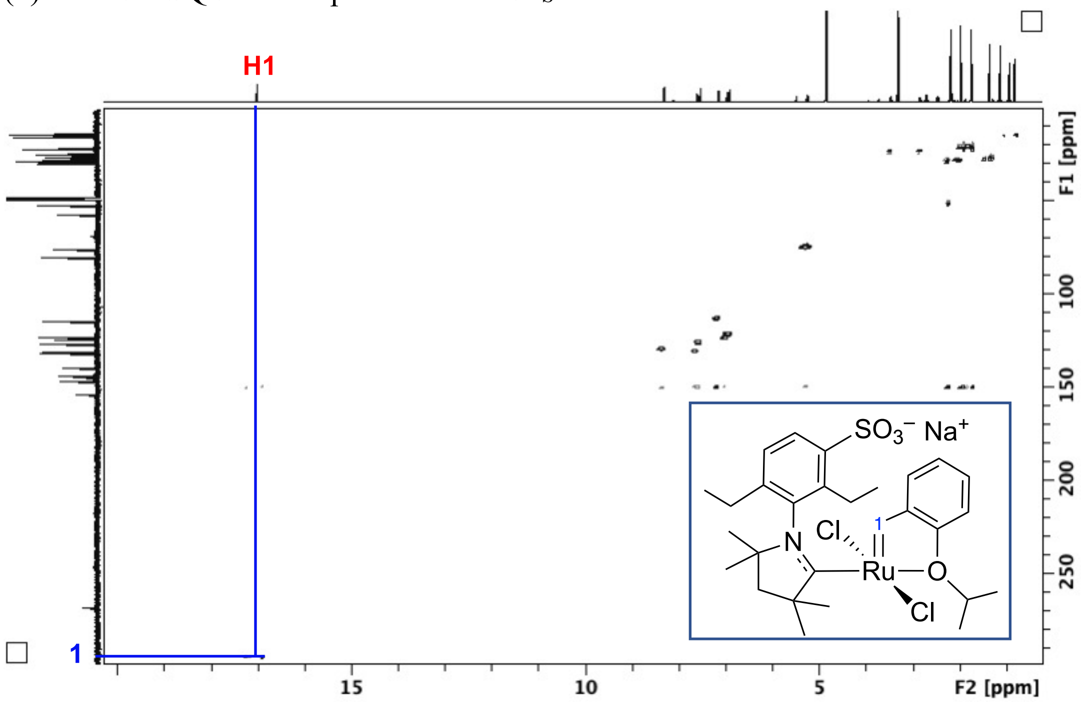

Figure continues next page

(e) ^{1}H-^{13}C HMBC NMR spectrum of **HC1s^{Me}**

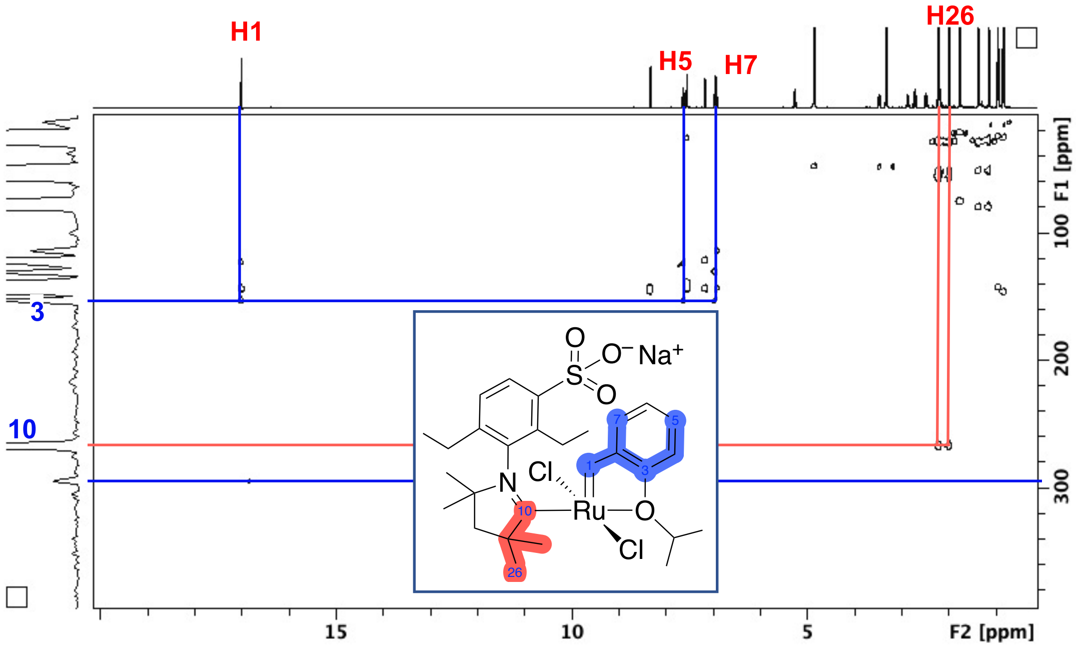

Figure S11. NMR characterization of **HC1s^{Me}** in CD_3OD. (a) ^{1}H NMR (300 MHz, CD_3OD); inset shows alkylidene signal. (b) ^{13}C{^{1}H} NMR (150 MHz). Alkylidene carbon signal not observed. (c) ^{1}H-^{1}H COSY NMR (600 MHz). (d) ^{1}H-^{13}C HSQC NMR (600/150 MHz) showing correlation for alkylidene carbon. (e) ^{1}H-^{13}C HMBC NMR (600/150 MHz).

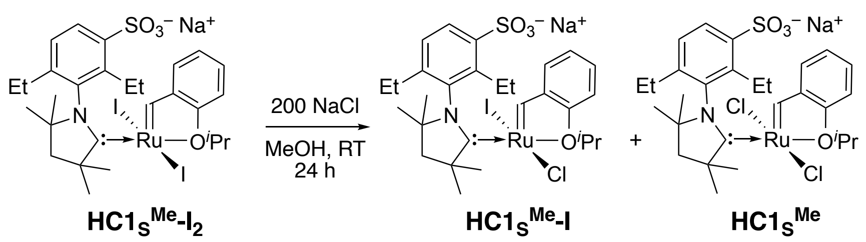

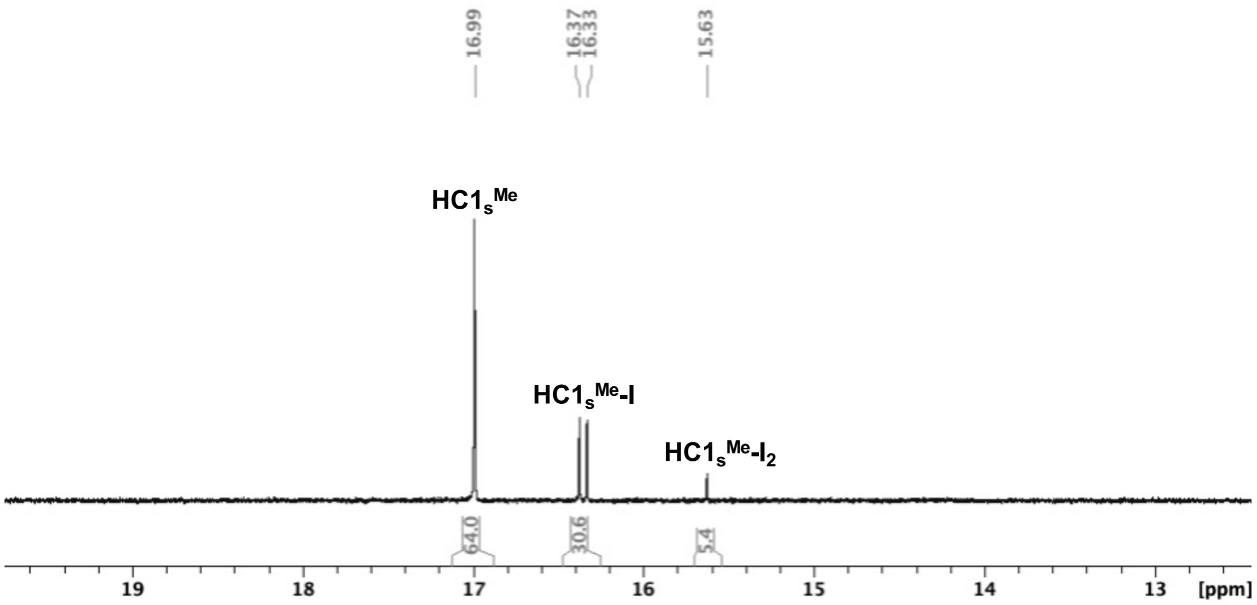

Figure S12. ^{1}H NMR (300 MHz, CD_3OD) spectrum for the reaction of **HC1$_S$^{Me}-I$_2$** + 200 equiv NaCl, after 24 h.

(a) ^{1}H NMR spectrum of **HC1s^{Cy}**

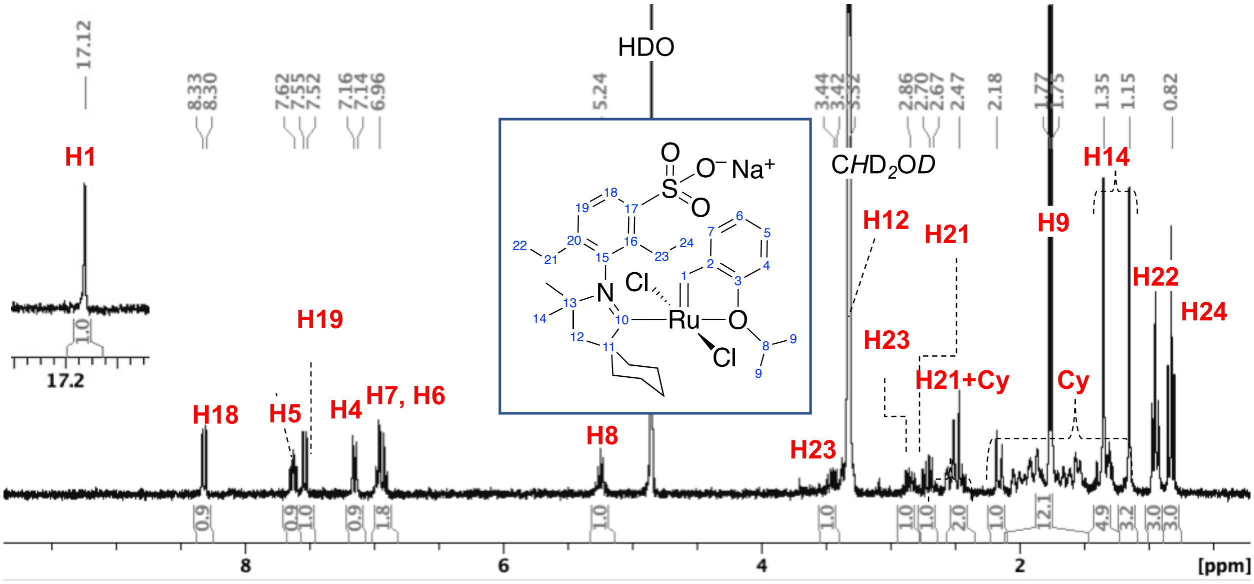

(b) ^{13}C{^{1}H} NMR spectrum of **HC1s^{Cy}**

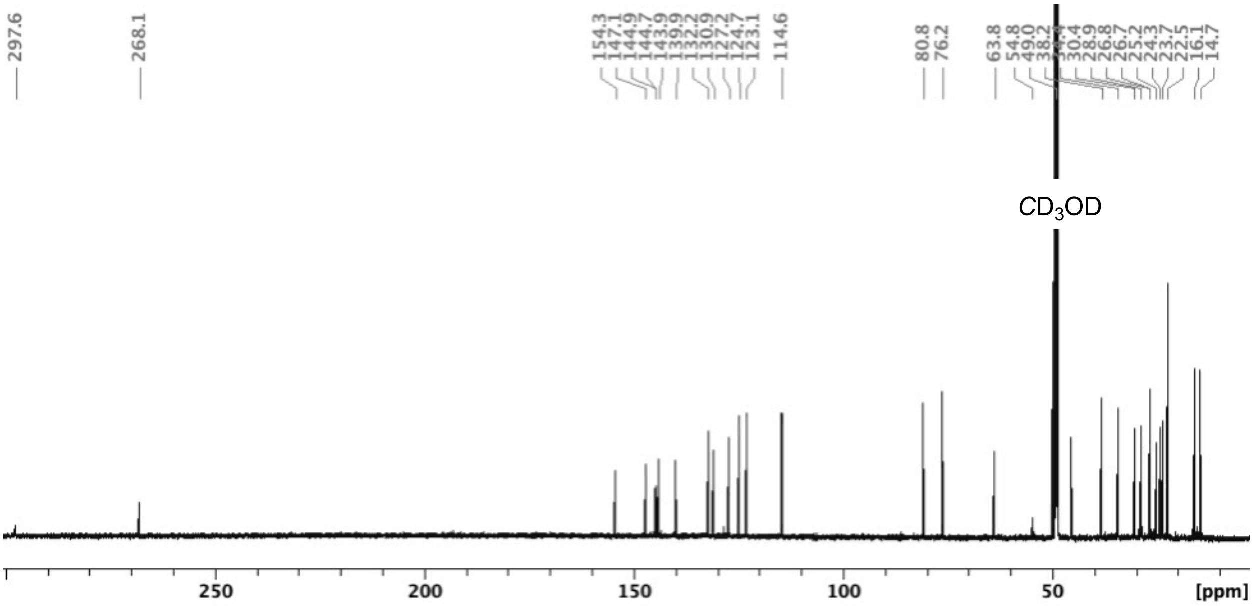

Figure continues next page

(c) ^{1}H-^{1}H COSY NMR spectrum of **HC1s^{Cy}**

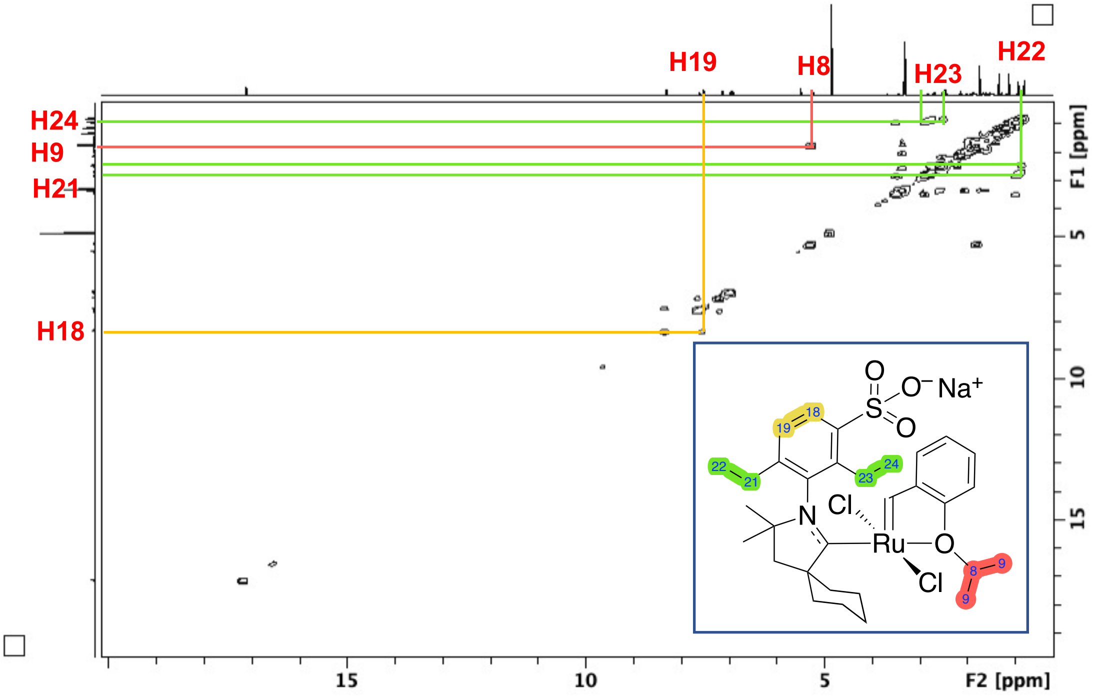

(d) ^{1}H-^{13}C HSQC NMR spectrum of **HC1s^{Cy}**

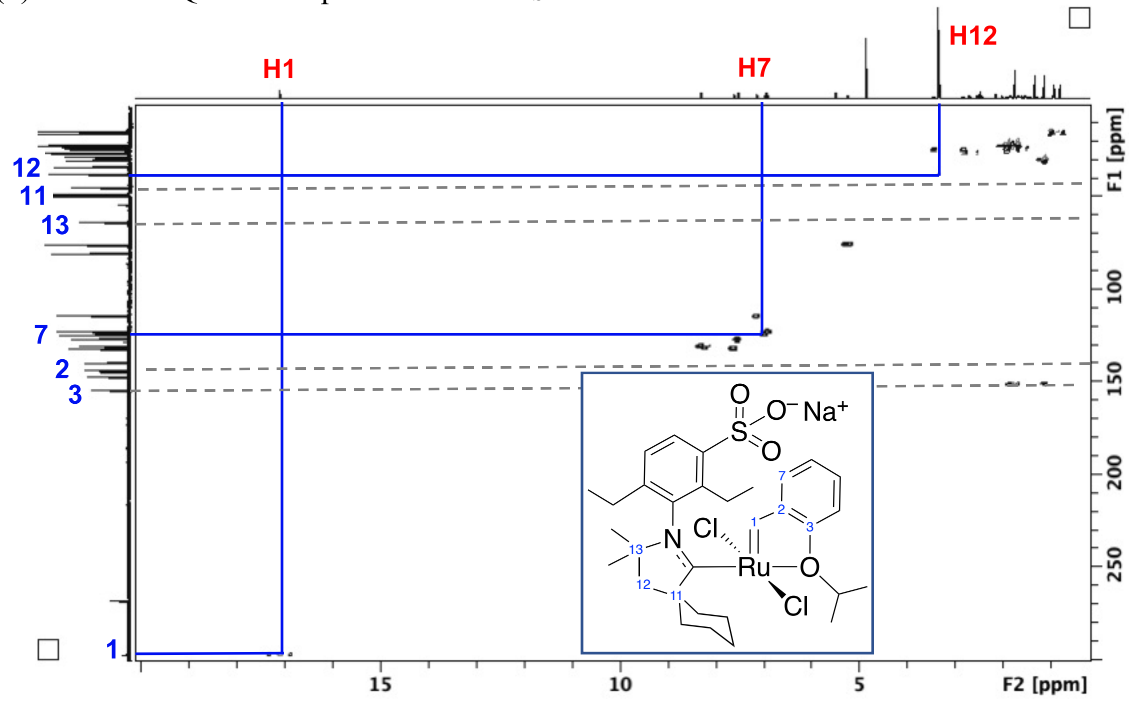

Figure continues next page

(e) ^{1}H-^{13}C HMBC NMR spectrum of **HC1s^{Cy}**

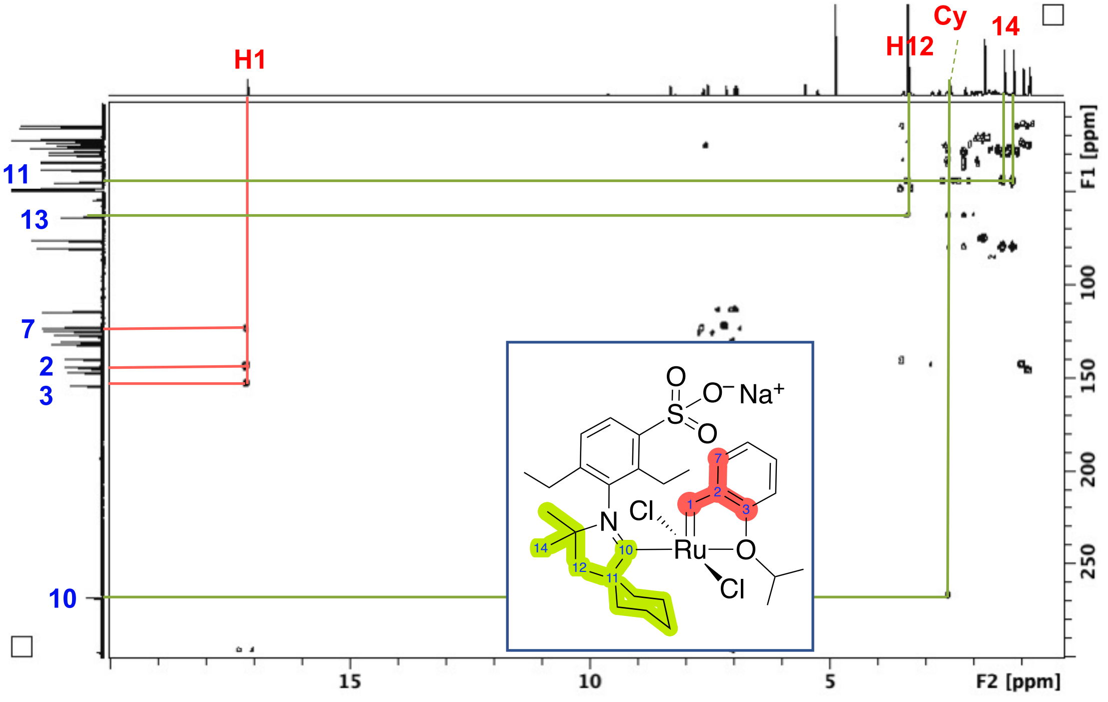

Figure S13. NMR characterization of **HC1s^{Cy}** in CD_3OD. (a) ^{1}H NMR (300 MHz; inset shows alkylidene signal. (b) ^{13}C{^{1}H} NMR (150 MHz). (c) ^{1}H-^{1}H COSY NMR (600 MHz). (d) ^{1}H-^{13}C HSQC NMR spectrum (600/150 MHz) (e) ^{1}H-^{13}C HMBC NMR (600/150 MHz).

(a) ^{1}H NMR spectrum of novel uridine substrate **7**

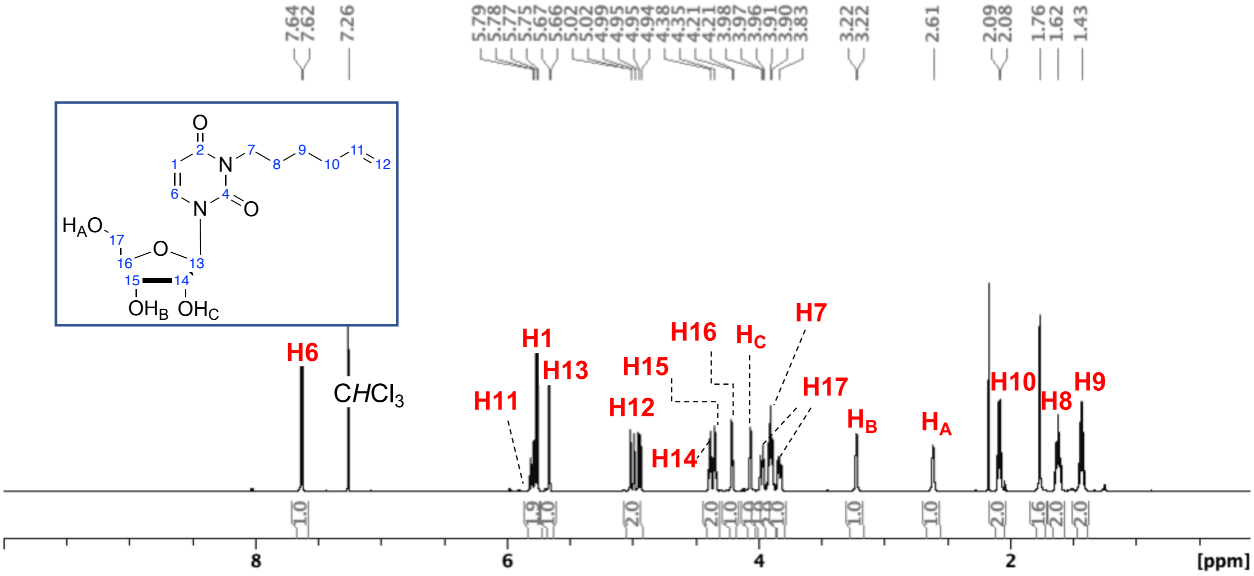

(b) ^{13}C{^{1}H} NMR spectrum of **7**

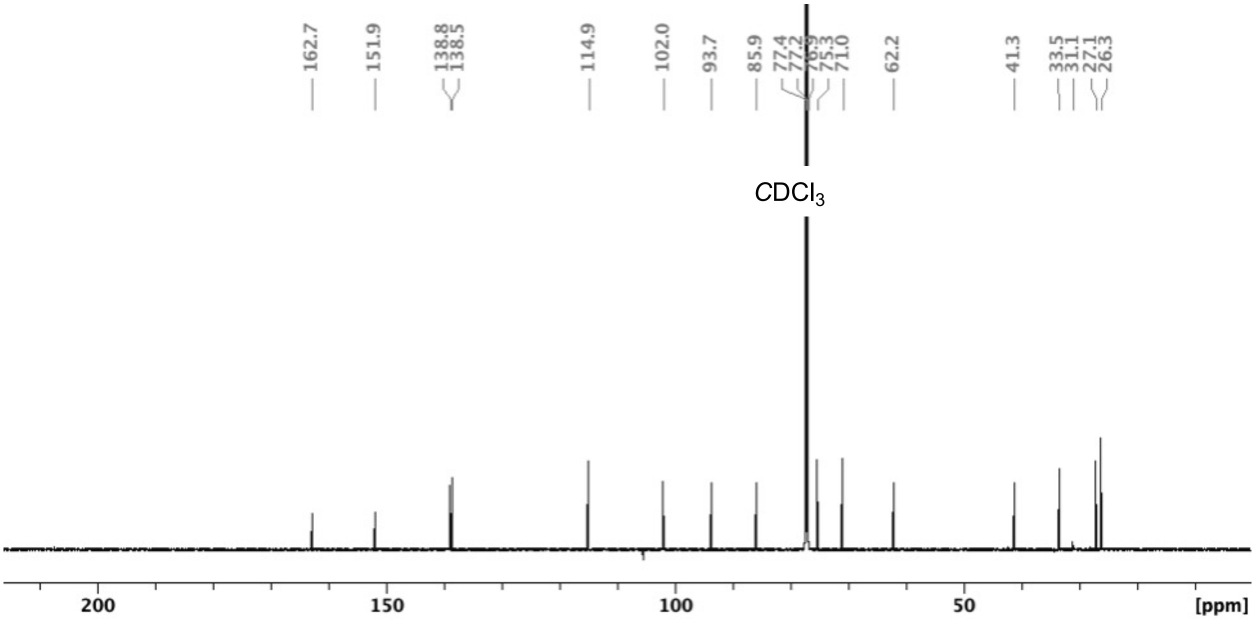

Figure continues next page

(c) ^{1}H-^{1}H COSY NMR spectrum of novel uridine substrate **7**

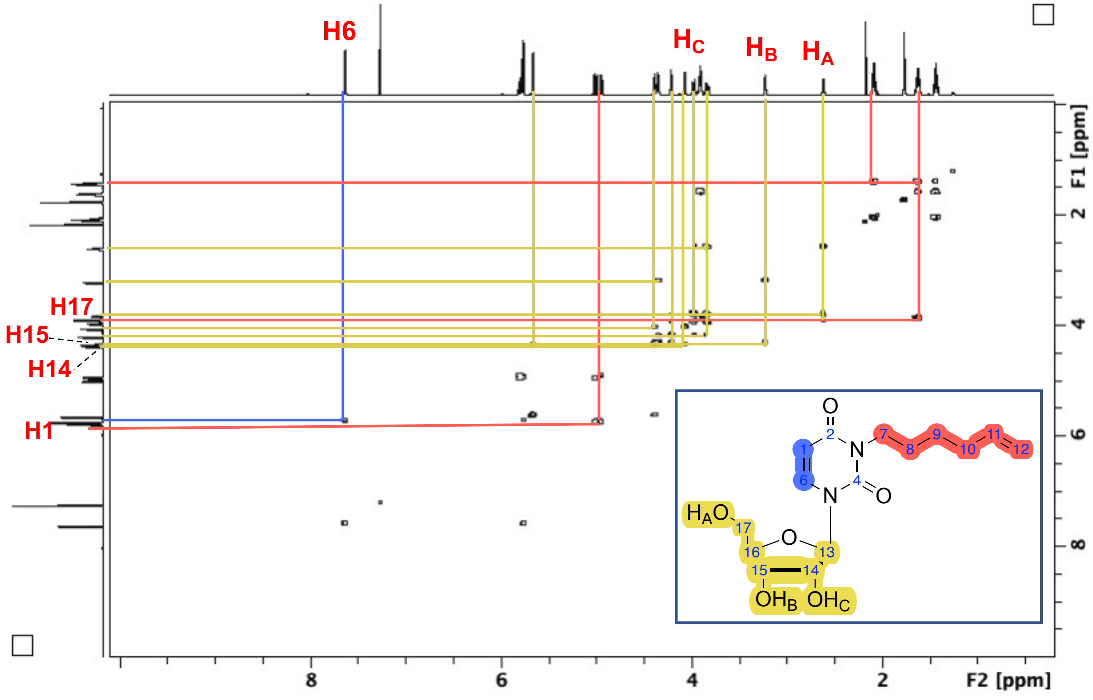

(d) ^{1}H-^{13}C HSQC NMR spectrum of **7**

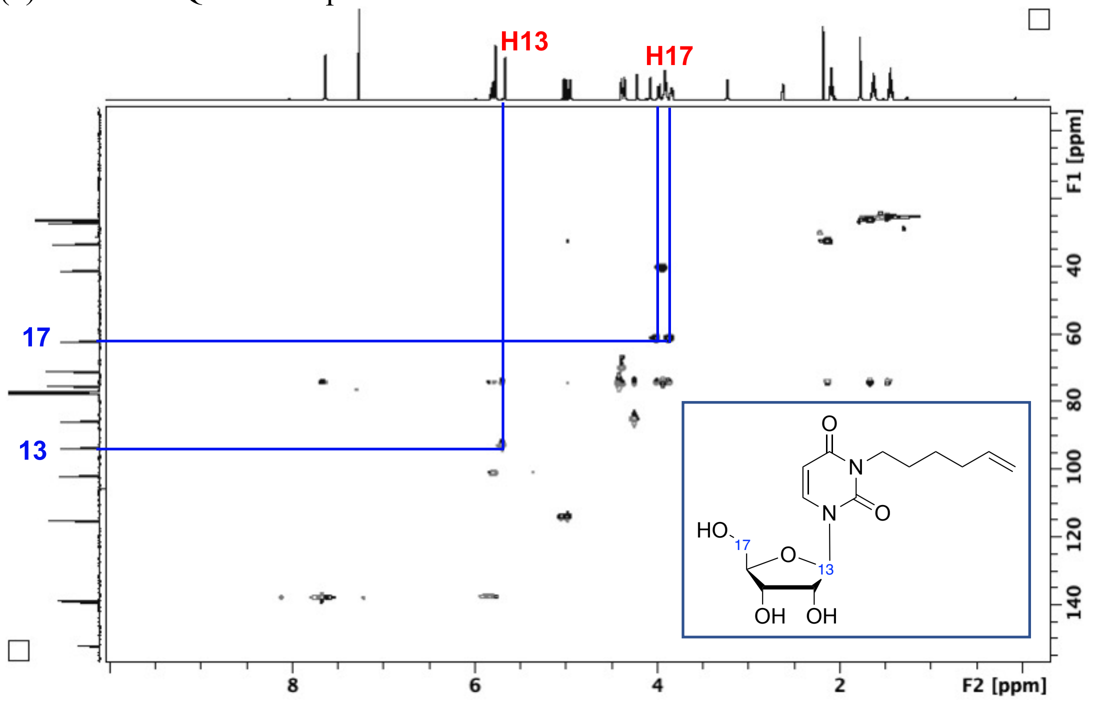

Figure continues next page

(e) ^{1}H-^{13}C HMBC NMR spectrum of novel uridine substrate **7**

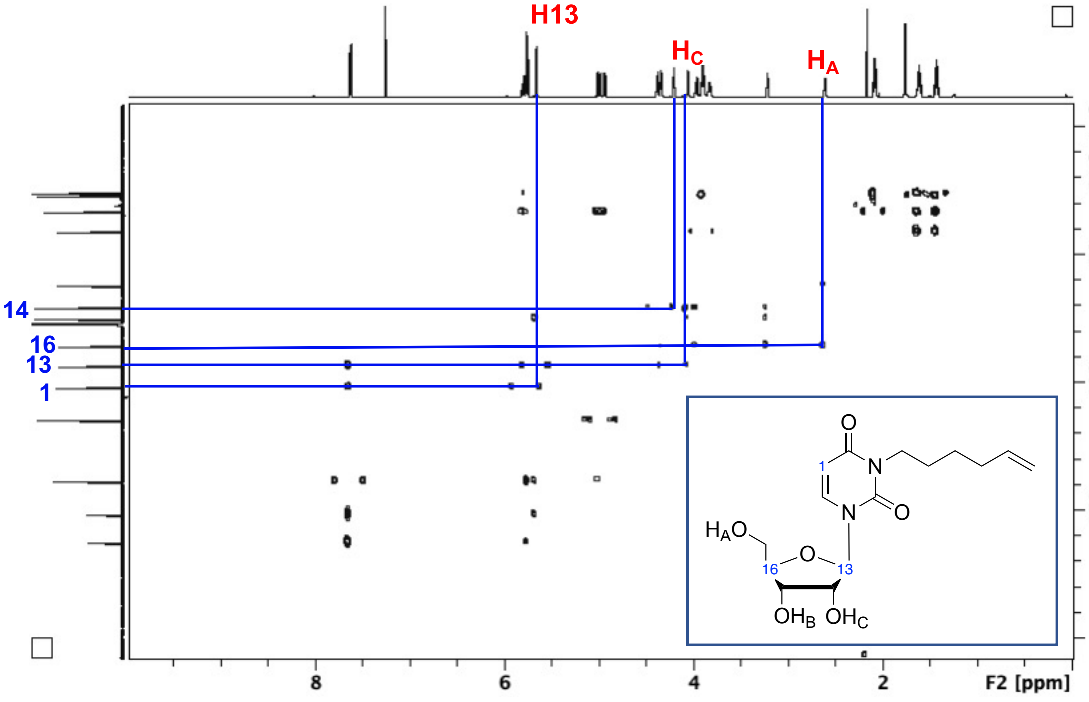

Figure S14. NMR characterization of uridine-tagged substrate **7** in $CDCl_3$. (a) ^{1}H NMR (600 MHz). (b) $^{13}C\{^{1}H\}$ NMR (150 MHz). (c) ^{1}H-^{1}H COSY NMR (600 MHz). (d) ^{1}H-^{13}C HSQC NMR spectrum (600/150 MHz). (e) ^{1}H-^{13}C HMBC NMR (600/150 MHz).

(a) ^{1}H NMR spectrum of novel uridine-tagged dimer **7'**

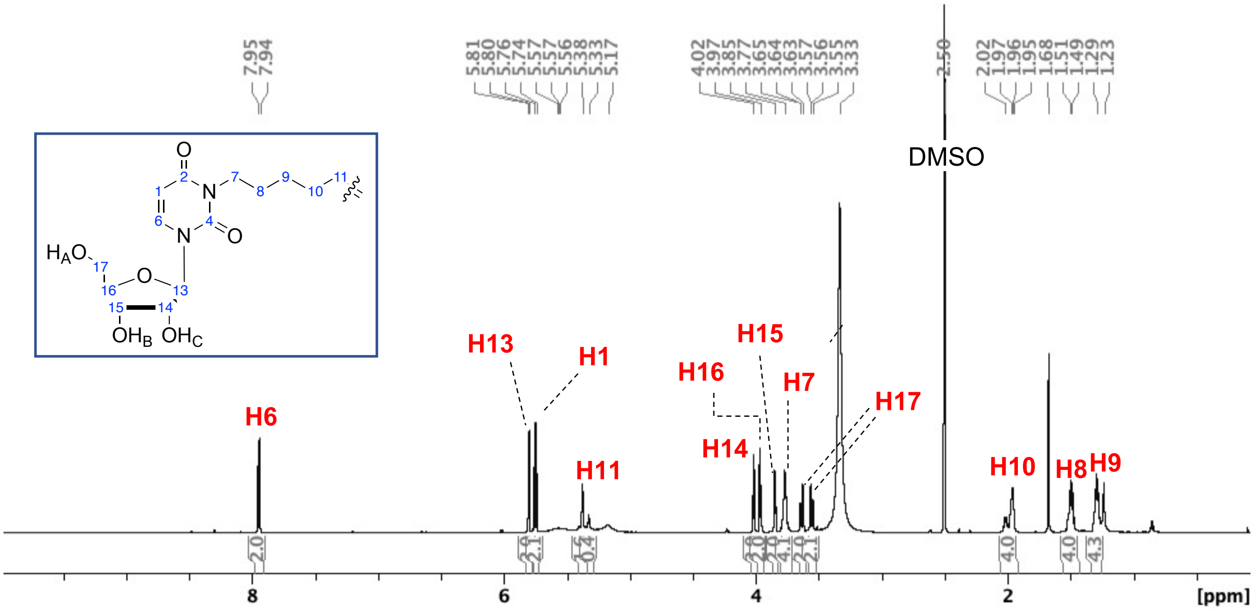

(b) $^{13}C\{^{1}H\}$ NMR spectrum of uridine-tagged dimer **7'**

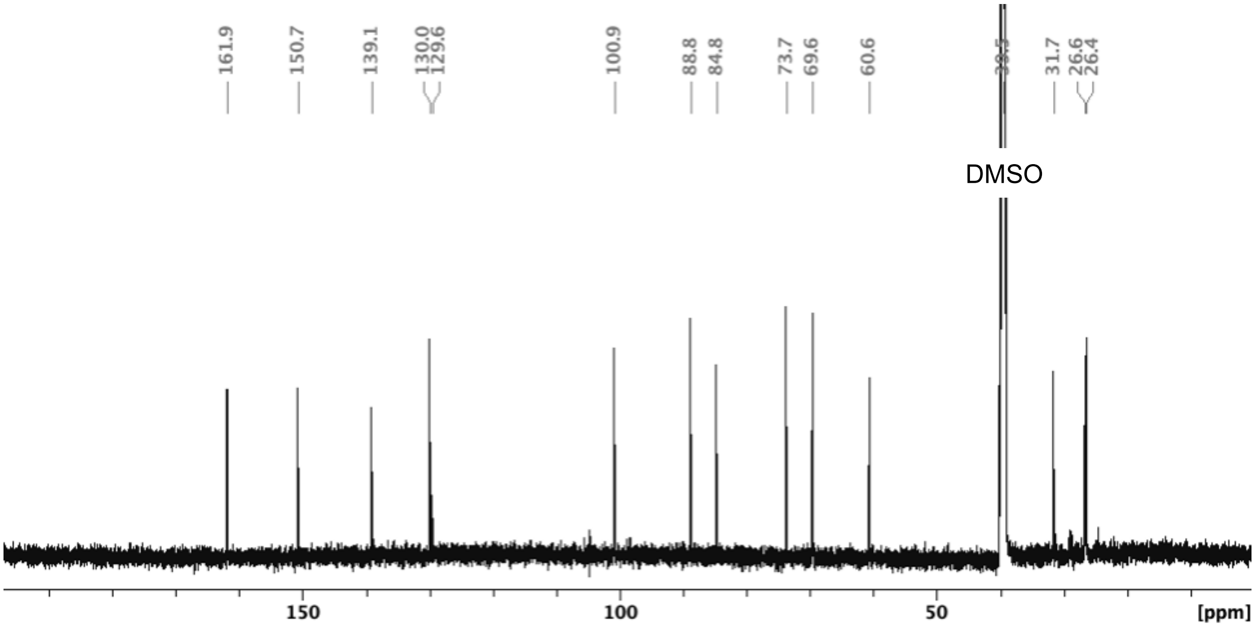

Figure continues next page

(c) ^{1}H-^{1}H COSY NMR spectrum of uridine-tagged dimer **7'**

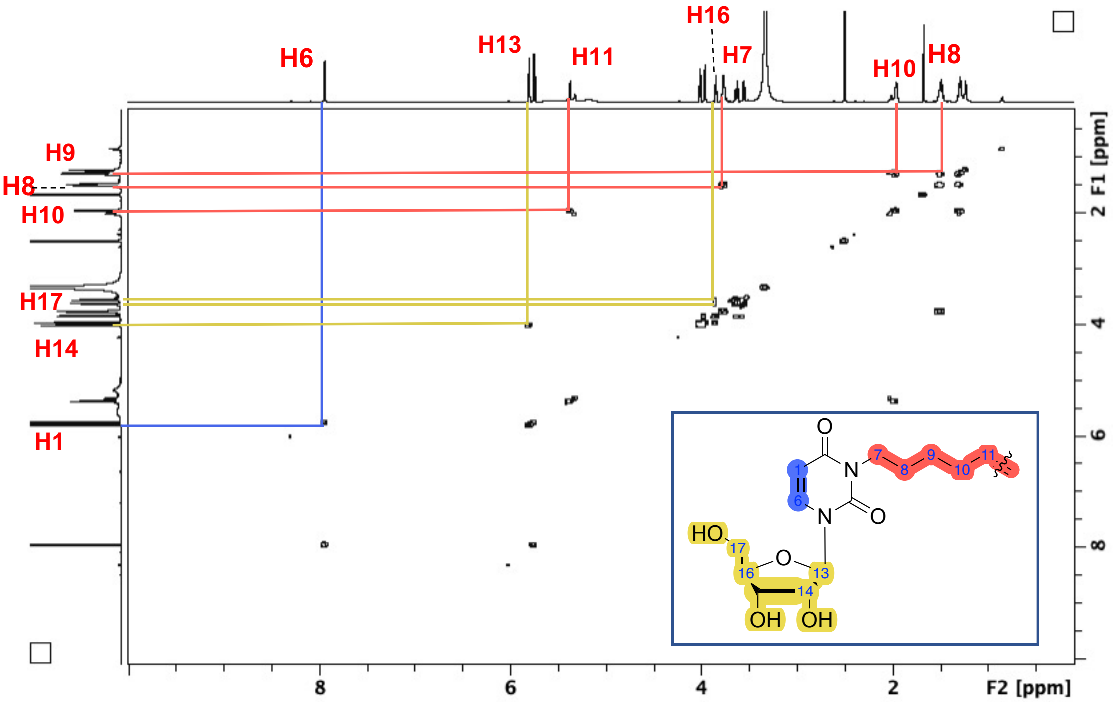

(d) ^{1}H-^{13}C HSQC NMR spectrum of uridine-tagged dimer **7'**

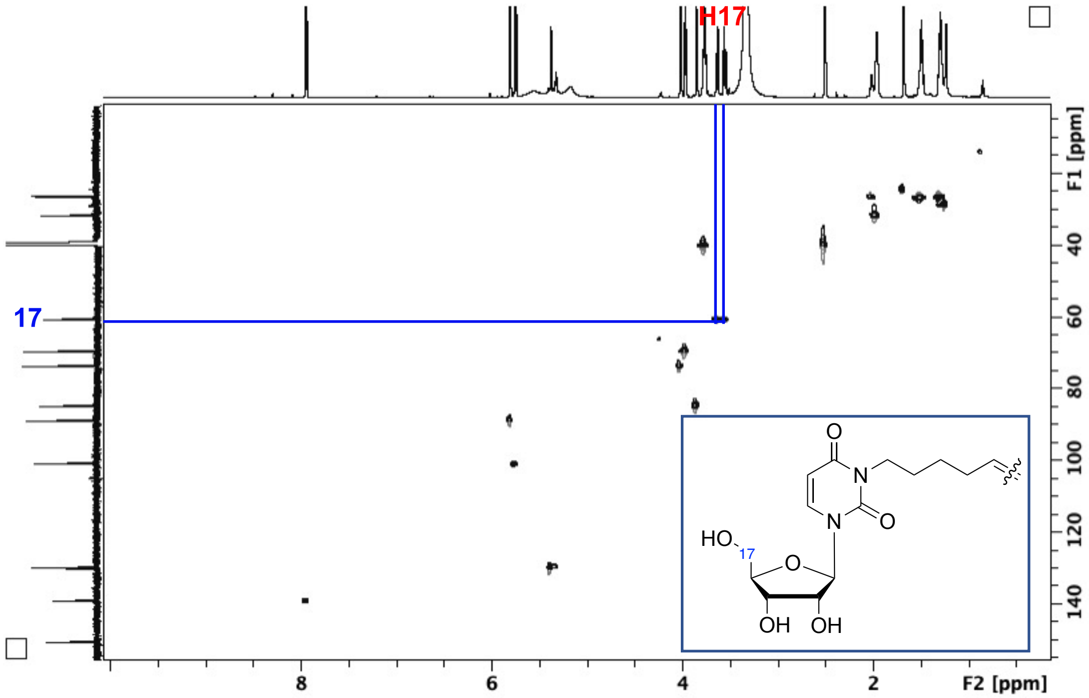

Figure continues next page

(e) ^{1}H-^{13}C HMBC NMR spectrum of uridine-tagged dimer **7'**

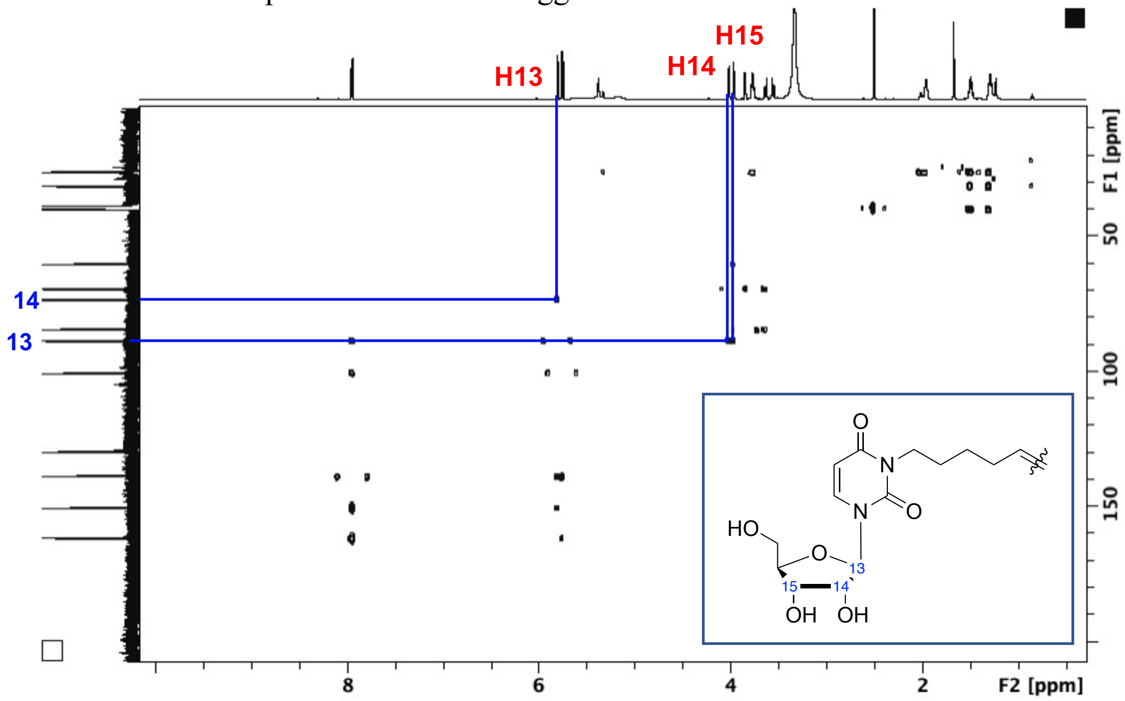

Figure S15. NMR characterization of novel uridine dimer **7'**, prepared via self-metathesis, in DMSO-d_6. (a) ^{1}H NMR (600 MHz). (b) $^{13}C\{^{1}H\}$ NMR (150 MHz). (c) ^{1}H-^{1}H COSY NMR (600 MHz). (d) ^{1}H-^{13}C HSQC NMR (600/150 MHz). (e) ^{1}H-^{13}C HMBC NMR (600/150 MHz).

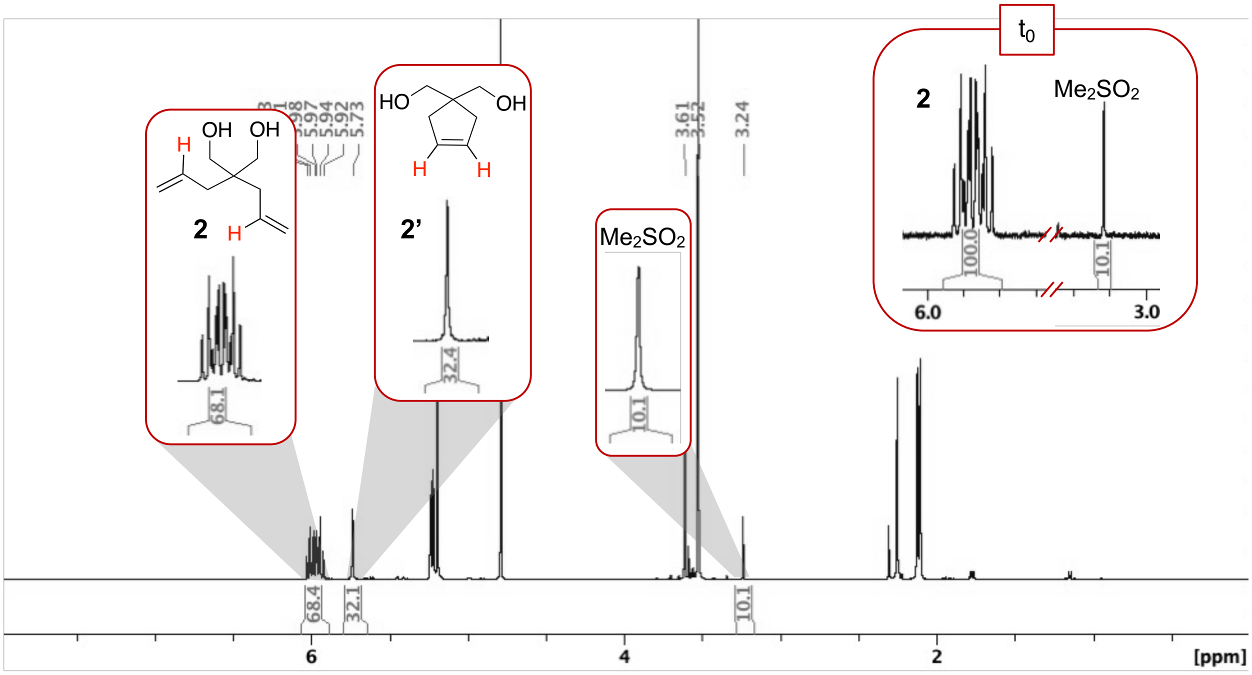

Figure S16. Quantifying RCM of **2** by **HC1s^{Me}** (0.05 mol%). Representative ^{1}H NMR spectrum (400 MHz, D_2O) at 32% conversion. Internal standard (IS) = dimethylsulfone, Me_2SO_2.

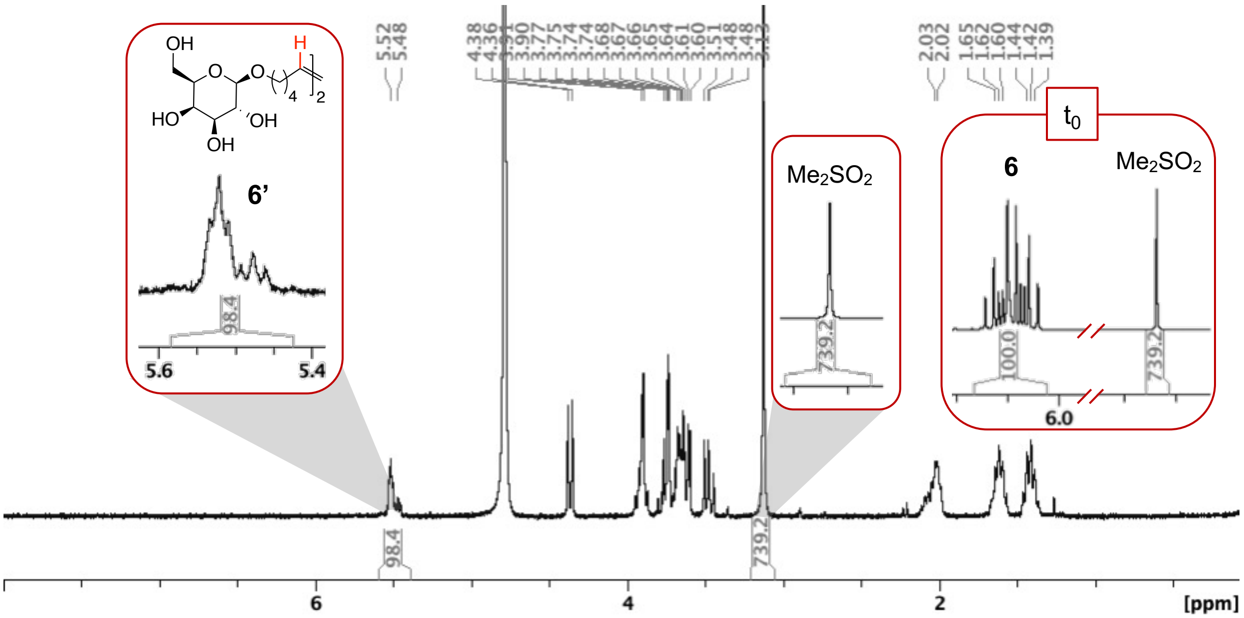

Figure S17. Quantifying dimerization of **6** by **HC1s^{Me}** (1 mol%). Representative ^{1}H NMR spectrum (300 MHz, D_2O) at 100% conversion. Internal standard (IS) = dimethylsulfone, Me_2SO_2.

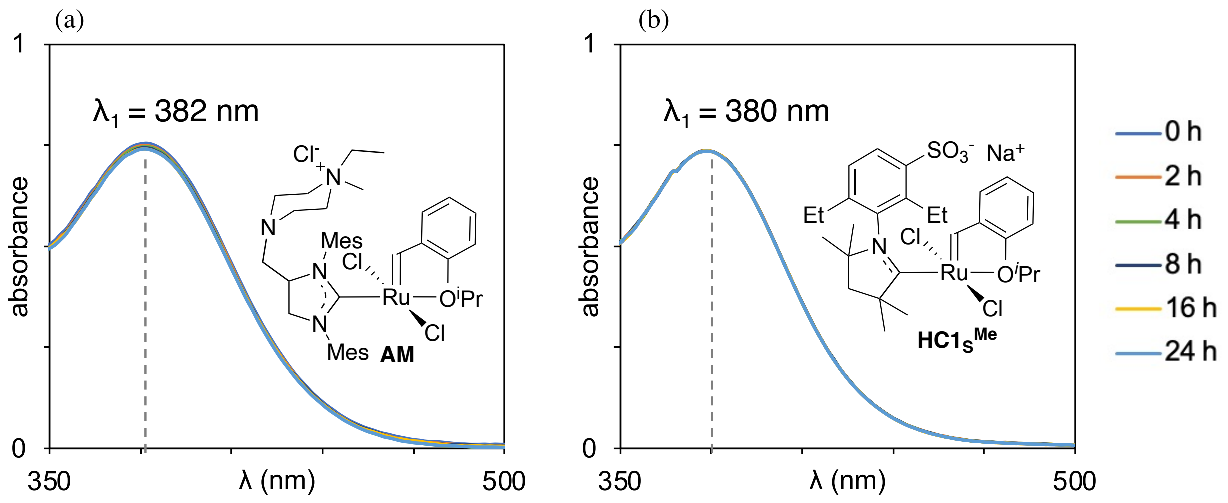

Figure S18. UV-vis spectra in H_2O + 2 M NaCl, showing λmax for dichlororuthenium complexes. (a) **AM**. (b) **HC1$_S$^{Me}**.

S3. References.

(1) Kingsbury, J. S.; Harrity, J. P. A.; Bonitatebus, P. J.; Hoveyda, A. H., A Recyclable Ru-Based Metathesis Catalyst. *J. Am. Chem. Soc.* **1999**, *121*, 791–799.

(2) Blanco, C.; Nascimento, D. L.; Fogg, D. E., Routes to High-Performing Ruthenium-Iodide Catalysts for Olefin Metathesis: Phosphine Lability Is Key to Efficient Halide Exchange. *Organometallics* **2021**, *40*, 1811–1816.

(3) Morvan, J.; Vermersch, F.; Zhang, Z.; Falivene, L.; Vives, T.; Dorcet, V.; Roisnel, T.; Crévisy, C.; Cavallo, L.; Vanthuyne, N.; Bertrand, G.; Jazzar, R.; Mauduit, M., Optically Pure C_1-Symmetric Cyclic(alkyl)(amino)carbene Ruthenium Complexes for Asymmetric Olefin Metathesis. *J. Am. Chem. Soc.* **2020**, *142*, 19895–19901.

(4) Escudero, J.; Bellosta, V.; Cossy, J., Rhodium-Catalyzed Cyclization of *O*,ω-Unsaturated Alkoxyamines: Formation of Oxygen-Containing Heterocycles. *Angew. Chem., Int. Ed.* **2018**, *57*, 574–578.

(5) Schmidt, V. A.; Alexanian, E. J., Metal-Free Oxyaminations of Alkenes Using Hydroxamic Acids. *J. Am. Chem. Soc.* **2011**, *133*, 11402–11405.

(6) Matsuo, T.; Yoshida, T.; Fujii, A.; Kawahara, K.; Hirota, S., Effect of Added Salt on Ring-Closing Metathesis Catalyzed by a Water-Soluble Hoveyda−Grubbs Type Complex To Form N-Containing Heterocycles in Aqueous Media. *Organometallics* **2013**, *32*, 5313–5319.

(7) Timmer, B. J. J.; Ramström, O., Acid-Assisted Direct Olefin Metathesis of Unprotected Carbohydrates in Water. *Chem. Eur. J.* **2019**, *25*, 14408–14413.

(8) Blacquiere, J. M.; Jurca, T.; Weiss, J.; Fogg, D. E., Time as a Dimension in High-Throughput Homogeneous Catalysis. *Adv. Synth. Catal.* **2008**, *350*, 2849–2855.

(9) Boisvert, E.-J. Y.; Ramos Castellanos, R.; Ferguson, M. J.; Fogg, D. E., Abstraction of Trifluoroborane from Tetrafluoroborate: Li^+-Assisted Borylation of Nucleophilic Carbenes. *ChemCatChem* **2024**, e202401003.

(10) Garakani, T. M.; Sauer, D. F.; Mertens, M. A. S.; Lazar, J.; Gehrmann, J.; Arlt, M.; Schiffels, J.; Schnakenberg, U.; Okuda, J.; Schwaneberg, U., FhuA-Grubbs-Hoveyda Biohybrid Catalyst Embedded in a Polymer Film Enables Catalysis in Neat Substrates. *ACS Catal.* **2020**, *10*, 10946–10953.

(11) Blanco, C.; Sims, J.; Nascimento, D. L.; Goudreault, A. Y.; Steinmann, S. N.; Michel, C.; Fogg, D. E., The Impact of Water on Ru-Catalyzed Olefin Metathesis: Potent Deactivating Effects Even at Low Water Concentrations. *ACS Catal.* **2021**, *11*, 893−899.
